# Engineering PHL7 for improved poly(ethylene terephthalate) depolymerization via rational design and directed evolution

**DOI:** 10.1016/j.checat.2025.101399

**Published:** 2025-08-21

**Authors:** Thomas M. Groseclose, Erin Kober, Matilda Clark, Benjamin Moore, Ramesh K. Jha, Zoe K. Taylor, Lexy A. Lujan, Gregg T. Beckham, Andrew R. Pickford, Taraka Dale, Hau B. Nguyen

**Affiliations:** 1 Bioscience Division, Los Alamos National Laboratory, Los Alamos, NM 87545, USA; 2 BOTTLE Consortium, Golden, CO 80401, USA; 3 Centre for Enzyme Innovation, School of the Environmental and Life Sciences, University of Portsmouth, Portsmouth PO1 2DT, UK; 4 Renewable Resources and Enabling Sciences Center, National Renewable Energy Laboratory, Golden, CO 80401, USA

**Keywords:** poly(ethylene terephthalate), PET hydrolase, PHL7, Protein engineering, high-throughput screening, directed evolution, enzymatic plastic degradation, plastic recycling, PETase modeling, split GFP

## Abstract

Enzymatic depolymerization of poly(ethylene terephthalate) (PET) has emerged as a promising approach for polyester recycling, and, to date, many natural and engineered PET hydrolase enzymes have been reported. For industrial use, PET hydrolases must achieve high depolymerization extent and exhibit excellent thermostability. Here, we engineered a natural PET hydrolase, Polyester Hydrolase Leipzig #7 (PHL7), through rational design and directed evolution using a high-throughput screening platform. Four new enzymes were engineered with enhanced properties compared with the parent enzyme, wild-type PHL7 (PHL7-WT), and other benchmark PET hydrolases, under the tested conditions. In bioreactors, the exemplary engineered enzyme, PHL7-Jemez, exhibited improved ability to depolymerize amorphous PET film compared with PHL7-WT at 2.9% and 20% substrate loadings, with 37% and 270% higher hydrolysis, respectively, after 48 h. This study develops several state-of-the-art PET hydrolases and demonstrates a directed evolution platform to engineer high-performance enzymes, which can accelerate enzyme discovery toward improved biocatalytic recycling.

## Highlights

• An adaptable high-throughput screening platform enables directed evolution of PHL7
• Four engineered PHL7 variants have higher activity and expression levels
• The top variant breaks down PET more efficiently than wild-type PHL7 in bioreactors
• Future work must address PHL7’s buffer needs with protein or process engineering

## The bigger picture

Since the discovery of natural enzymes that can break down poly(ethylene terephthalate) (PET) plastic to its chemical building blocks, researchers have envisioned using large-scale, industrial enzymatic recycling to deal with this waste plastic. However, this technology is not yet cost competitive, in part due to suboptimal efficiency of the enzymes, particularly in breaking down post-consumer PET plastic. This study demonstrates a powerful engineering platform that discovers enhanced PET-degrading enzyme variants by sorting through large numbers of candidate enzymes, evaluating them for the properties that are most critical for their successful deployment in industrial enzymatic PET recycling. This platform can accelerate the discovery of enzymes that can act as solutions to existing bottlenecks in enzymatic PET recycling and can be readily adapted to develop enzymes designed to address future challenges, promoting a technology that has the potential to mitigate plastic pollution.

## Graphical abstract

**Figure:**
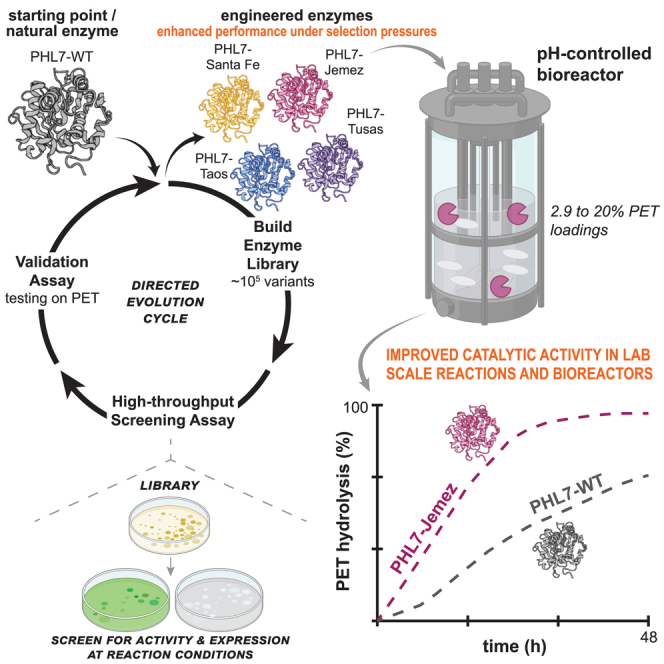

## Teaser

Enzymatic recycling is a potential route to mitigate accumulating PET plastic waste; however, to be cost competitive, critical properties of current PET-degrading enzymes must be improved. Here, we create four new variants of the PHL7 enzyme using rational design using a high-throughput screening platform for engineering PET-degrading enzymes. The variants have higher protein expression and enhanced PET degradation efficiency compared with benchmark enzymes. This platform facilitates the development of new enzymes that could make enzymatic PET recycling more competitive.

## Introduction

Polyethylene terephthalate (PET), prevalent in packaging and textiles, is one of the most-produced plastics globally, with an estimated worldwide production of 25 million metric tons per year.^1^ In recent years, enzymes that are able to break down PET into its terephthalic acid (TPA) and ethylene glycol (EG) monomers have gained increasing attention, with promise for industrial recycling of PET waste streams.^2^ However, enzymes sourced from natural diversity are often not yet optimized for use in industrial chemical recycling processes,^3^^,^^4^ but do provide footholds for engineering improved function. Tournier et al. reported the successful engineering of one of these enzymes, leaf-branch compost cutinase (LCC),^5^ to create the optimized variant LCC-ICCG,^6^ which is one of the most efficient PET-degrading enzymes reported to date.^7^ Other, recent successes in engineering high-performance PET hydrolases include LCC-A2,^8^ Kubu-P^M12^,^9^ and LCC-LANL.^10^

Enzymatic PET depolymerization has been reported to be most efficient at around 70°C for thermostable PET hydrolases.^2^^,^^6^^,^^11^^,^^12^ A major goal of PET hydrolase engineering, therefore, has been to increase enzyme thermostability,^2^^,^^6^^,^^13^^,^^14^^,^^15^^,^^16^^,^^17^ allowing the enzymes to retain activity at elevated temperatures throughout long reaction times. Previous work to increase thermal stability has been achieved for LCC and *Ideonella sakaiensis* PETase (*Is*PETase) through the addition of disulfide bonds^6^^,^^18^ or salt bridges.^13^ Richter et al. recently reported the engineering of a salt bridge in Polyester Hydrolase Leipzig #7 (PHL7), a recently reported thermophilic polyester hydrolase,^16^^,^^19^ via residues coordinating a metal binding site in the crystal structure, E148 and D233K, which increased the melting temperature (T_m_) by 0.9°C.^16^ This metal binding site, however, is distinct from the previously engineered sites in both LCC and *Is*PETase.^5^^,^^6^^,^^17^^,^^18^

In this work, we used rational design to explore the best mutations for thermostability improvement and directed evolution with a recently reported high-throughput (HT) screening platform^10^ to engineer high-performance PET hydrolases using PHL7 as the template. The engineered enzyme variants reported here were conferred with improved catalytic activity, enhanced stability, higher expression, and were able to depolymerize PET more efficiently compared with the wild-type enzyme, demonstrating the efficiency of our HT screening platform to evolve diverse PET hydrolases for a range of engineering goals. In small-scale reactions, at low substrate loadings and under some tested conditions, the engineered PHL7 enzyme variants surpassed the catalytic activity of two benchmark enzymes, LCC-ICCG^6^ and PHL7-L93F/Q95Y.^17^ We note here that the enzyme previously reported in the literature as PHL7 by Sonnendecker and colleagues^16^^,^^19^ has also been called PES-H1, based on an original patent application,^20^ and is referred to by that name by several publications, including Pfaff et al.^17^ and several others.^2^^,^^7^^,^^21^^,^^22^ In this work, we chose to refer to the enzyme as PHL7, as previously reported by Sonnendecker and colleagues, and maintain their protein sequence numbering scheme. We also want to point out that, due to the inconsistency in the numbering of this enzyme protein sequence from previous publications, the PES-H1 L92F/Q94Y variant reported by Pfaff et al.^17^ corresponds in this work to PHL7-L93F/Q95Y.

## Results

### Rational design to improve PHL7 thermostability and catalytic activity and identify potential templates for directed evolution

To improve enzyme thermostability, we first examined disulfide bonds and salt bridges in PHL7, focusing on the analogous sites to D238C/S283C from LCC-ICCG,^6^ positing that at least one could confer increased thermostability, ideally not at the sacrifice of enzyme activity. Testing the disulfide bond variant, PHL7-R205C/S251C, previously reported by Pfaff et al.,^17^ as R204C/S250C, we observed a decrease in activity, but did observe an increase in thermostability, with approximately 20% less activity by 72 h, but 19% more protein retained after heat treatment at 73°C for 1 h (Figure S1). Due to the loss in activity observed with a disulfide bond at position R205C/S251C, we therefore, constructed two salt bridge variants based on the analogous sites to E204/N233K from *Is*PETase: PHL7-Q175E (bridging to R205) and PHL7-Q175E/R205K, hypothesizing that different sidechains of positively charged residues at position 205 may better facilitate intramolecular electrostatics, and mutating glutamine to glutamate would be amenable to constructing a salt bridge without disrupting the enzyme structure. We determined that both the Q175E (bridging to R205) and Q175E/R205K salt bridge constructs conferred increased thermostability to PHL7 through a crude cell lysate-based screen via split GFP complementation,^10^ though the double-mutation construct (Q175E/R205K) increased the thermostability more than Q175E alone. Compared with wild-type PHL7 (PHL7-WT), after a 72-h reaction at 70°C, PHL7-Q175E showed 11.5% higher activity, and, separately, 23.5% more protein retained after being heat treated at 73°C for 1 h (to test thermostability), while PHL7-Q175E/R205K showed 44% higher activity and 45% more protein retained under the same conditions (Figure S1). The PHL7-Q175E/R205K variant was, therefore, selected as one of the templates for our directed evolution of PHL7 enzyme.

**Figure 1:**
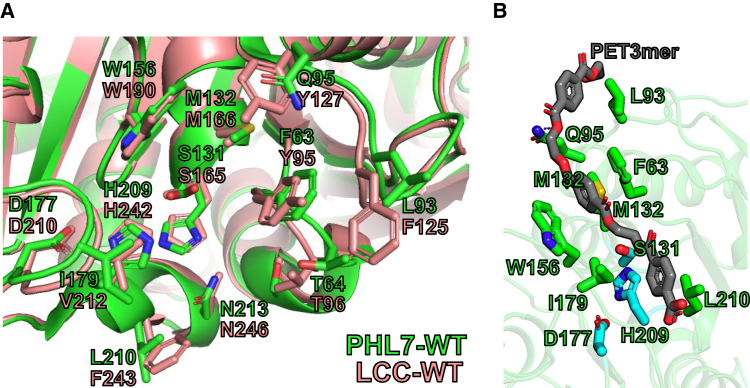
Structural modeling of PET hydrolases (A) Comparison of the active site structures of PHL7-WT (chain A, green color) and LCC-WT (salmon color). Site-saturation mutagenesis was performed for the five positions at which the sequence of PHL7 and LCC differed: F63, L93, Q95, I179, and L210. (B) Computational docking with PET3mer substrate shows how the five residues of PHL7-WT selected for site-saturation mutagenesis interact with the substrate.

In addition to testing disulfide bonds and salt bridges, site-saturation mutagenesis was explored for the PHL7 active site, as previous rational and semi-rational design efforts of an enzyme’s active site have success in increasing PET hydrolase activity.^2^^,^^6^^,^^13^^,^^14^^,^^17^^,^^23^ Site-saturation mutagenesis was performed for the active site of PHL7, at five positions at which the sequence of PHL7 and LCC differ: F63, L93, Q95, I179, and L210 (Figure 1). These positions span regions of the protein that are responsible for both TPA binding (subsite I), and regions that are hypothesized to facilitate initial binding of substrate and guidance of the scissile bond toward the active site (subsite II).^16^ The five site-saturation mutant libraries generated were screened for protein expression/solubility and catalytic activity using our HT screening assay with bis(2-hydroxyethyl) terephthalate (BHET) as a model substrate.^10^

As reported elsewhere^16^^,^^17^ and confirmed using our HT screening assay, several enzyme variants in these five site-saturation mutant libraries showed increased activity. These variants—F63Y, L93F, L93Q, L93A, Q95S, Q95Y, Q95G, I179V, L210V, L210T, and L210F—were selected as templates for conducting directed evolution of PHL7. The performance of these variants is shown in Table 1, including their positions mapped to the protein structure and comparison with the performance values reported in previous studies, if any. Among these single-point mutations, a top-performing enzyme mutant, Q95Y, showed both increased activity and thermostability, with 50% higher activity in 72 h toward amorphous PET coupons and 19.5% more protein retained after heat treatment at 73°C for 1 h compared with PHL7-WT (Figure S1).

**Table 1:** Performance of site-saturation mutants

Mutant	Average fold change activity over PHL7-WT	Reported value (vs. PHL7-WT) from previous studies	Protein region
F63Y	1.18 ± 0.057	0.65-fold activity; ΔT_m_ = 0°C (Pfaff et al.^17^); 0.99-fold activity (Richter et al.^16^)	subsite I
L93F	1.09 ± 0.011	1.22-fold activity (Richter et al.^16^)	subsite I
L93Q	1.06 ± 0.02	*not previously reported*	subsite I
L93A	1.04 ± 0.015	1.06-fold activity (Richter et al.^16^)	subsite I
Q95S	1.42 ± 0.057	*not previously reported*	subsite I
Q95G	1.07 ± 0.02	0.88-fold activity (Richter et al.^16^)	subsite I
Q95Y	1.97 ± 0.069	1.15-fold activity (Richter et al.^16^)	subsite I
I179V	1.17 ± 0.01	*not previously reported*	subsite I
L210V	1.4 ± 0.032	0.65-fold activity; ΔT_m_ = 0°C (Pfaff et al.^17^); 1.38-fold activity (Richter et al.^16^)	subsite II
L210T	1.04 ± 0.039	0.48-fold activity; ΔT_m_ = 1°C (Pfaff et al.^17^); 1.42-fold (Richter et al.^16^)	subsite II
L210F	1.07 ± 0.03	0.60-fold activity; ΔT_m_ = 3°C (Pfaff et al.^17^); 0.69-fold activity (Richter et al.^16^)	subsite II
R205C/S251C	0.8 ± 0.066	1.00-fold activity; ΔT_m_ = 7°C (Pfaff et al.^17^)	N/A

Activity was measured by UV absorbance and expressed as equivalents of BHET, standardized to the activity of PHL7-WT. Enzymes were reacted as cell lysates at a final concentration of 0.05 μM enzyme with 0.92% (w/v) amorphous PET coupons, at 70°C in 1 M potassium phosphate buffer, pH 8, with the activity measurement taken after 6 h. The average is from *n* = 2 reactions, with the error ±1 SD. The reported value was taken from data from Pfaff et al.^17^ or Richter et al.^16^ at the 4-h timepoint. Protein region corresponds to the classification by Richter et al.^16^ The double-mutant L93F/Q95Y engineered by Pfaff et al.^17^ combines the mutations L93F and Q95Y, but individual mutations were not explicitly tested.

### Directed evolution of PHL7

**Figure 2:**
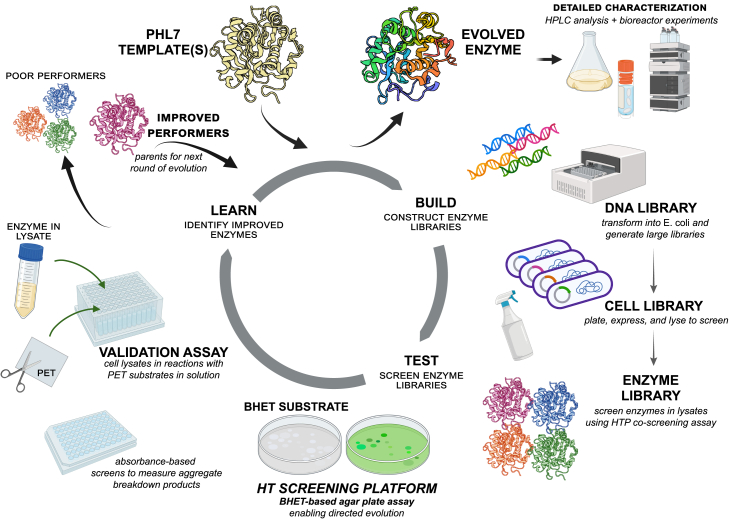
Workflow for engineering PHL7 in this study In each round of directed evolution, PHL7 templates (from rational/semi-rational design or previous rounds of evolution) were chosen as parents for mutagenesis via DNA shuffling and screening. Enzyme libraries resulting from DNA library construction and transformation into *E. coli* were screened using the BHET/split GFP HT co-screening assay. Improved variants were then validated in assays with PET substrates, in solutions, via monitoring soluble aromatic products release over time. Confirmed “hits” were isolated and sequenced, then were used as parents for the next round of directed evolution. Once significantly improved enzymes were identified, they were chosen for large-scale production and thorough characterization, including monomer quantification with high-performance liquid chromatography (HPLC), evaluation in bioreactors, and differential scanning calorimetry (DSC).

We recently developed an HT screening platform for engineering PET hydrolases, which is capable of simultaneously screening large, random mutagenesis enzyme libraries for improved protein solubility, activity, and thermostability by coupling a split GFP assay and a BHET hydrolysis assay.^10^ Specifically, enzyme variants were expressed with C-terminal GFP11 tags using the vector indicated in Table S1, which allows their quantification in crude cell lysates, in solutions or on agar plates, via green fluorescent readout when complemented with GFP1-10,^24^ thus eliminating the need for protein purification during the screening steps, which ultimately increases the throughput and reduces the cost. Activity was evaluated stepwise, first on a model substrate using BHET, then on PET substrates. Enzyme activity was first screened on BHET agar plates based on clearing zones (halos) generated from BHET hydrolysis. Briefly, enzyme libraries were first grown on Durapore membranes on LB agar plates overnight, then protein expression was achieved by transferring the membranes to plates containing isopropyl β-ᴅ-1-thiogalactopyranoside (IPTG) for induction. Durapore membranes containing bacterial colonies were then moved to BHET agar screening plates, colonies were partially lysed by spraying with BugBuster, allowing the cell lysates to diffuse through the membranes onto the BHET agar plates, and membranes were finally returned to LB agar plates and stored at 4°C for later colony picking. The BHET agar plates were then incubated at the reaction temperature and colonies were selected based on higher enzyme activity (larger clearing zones) and/or greater expression levels (higher fluorescence intensities) after aligning BHET screening plates to the original colonies grown on the membranes. Coupling the fluorescence and colorimetric assays, we were able to quickly and precisely select enzyme variants with improved both activity and expression/thermostability concurrently.^10^ Selected enzymes were then expressed in small scale (2–25 mL) and validated on reactions with PET substrates, in solution, with soluble aromatic products measured in microwell plates using established, plate-based absorbance protocols.^10^^,^^25^^,^^26^ Enzyme variants displaying higher expression/solubility, activity, and/or thermostability compared with the parent enzymes from the previous round were sequenced and selected as parents for the next round of evolution. The workflow for directed evolution of PHL7 enzyme is illustrated in Figure 2.

Throughout the directed evolution process of PHL7, increasing magnitudes of selection pressures were implemented after each round of evolution including (1) increasing BHET concentrations (starting at 20 mM, and gradually increasing up to 120 mM), (2) increasing reaction durations (starting at 6 h, and gradually increasing up to 48 h), (3) increasing heat treatment time at 75°C before enzyme reactions on BHET plates (starting at 1 h then increasing to 2 h), and (4) increasing PET solids loadings in reactions (from 0.92% [w/v] to 2.9% [w/v]) in validation assays with PET substrates.

**Figure 3:**
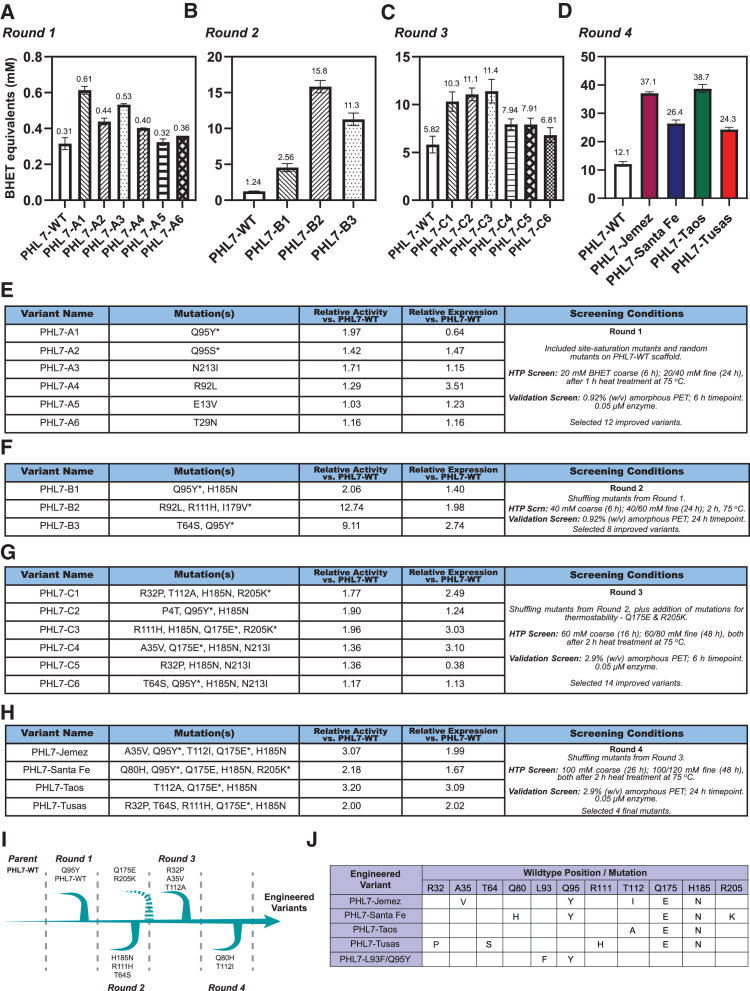
Screening and evolutionary trajectory of evolved PHL7 variants (A–D) Performance of evolved PHL7 variants across rounds of directed evolution. Activity is expressed in terms of equivalents of BHET, measuring aromatic products via UV absorbance. (A) Round 1. (B) Round 2. (C) Round 3. (D) Round 4. Reactions consisted of 0.05 or 0.1 μM enzyme in cell lysates with either 0.92% or 2.9% (w/v) PET amorphous coupons at 70°C in 1 M potassium phosphate buffer, pH 8. Activity was measured either after 6 or 24 h. Variants are shown across the bottom axes, with bars showing mean of *n* = 2 replicates and error bars representing ±1 S.D. (E–H) Tables showing details of variants and screening conditions for each round of evolution. Note that screening conditions varied between each round. Names of variants are shown with corresponding mutations compared with PHL7-WT, as well as relative performance vs. PHL7-WT, expressed as fold change of activity vs. PHL7-WT, and relative expression vs. PHL7-WT, measured using split GFP complementation of cell lysates. Screening conditions include details on the colony plate-based coarse and fine screenings and validation screenings, including how many colonies were chosen from each round of evolution. Top selected, representative variants are shown that directly led to mutations in future rounds of evolution (namely, the mutations in final variants). In mutations listed, the asterisk denotes mutations originating from rational design (i.e., not random mutations). (E) Round 1. (F) Round 2. (G) Round 3. (H) Round 4. (I) Evolutionary trajectory of final, engineered PHL7 variants. Mutations/scaffolds are shown that were introduced in each round of evolution. The dashed arrow denotes that scaffolds Q175E and R205K were introduced manually (not through DNA shuffling) between Round 2 and Round 3. Sequences of the final mutants are shown, as a combination of mutations incurred in Rounds 1–4. (J) Table of mutations of each of the four engineered mutants from this paper, as well as the PHL7-L93F/Q95Y variant. For each variant, the mutation at a position compared to the PHL7-WT sequence is shown.

Directed evolution was first performed using PHL7-WT as the starting template. Libraries of DNA fragments containing random mutations were created using DNA shuffling, cloned into the pET21b(+)-GFP11 screening vector with a C-terminal GFP11 tag,^10^ and transformed into *E. coli*. Enzyme libraries were then screened using the HT co-screening assay, first with coarse screening, followed by fine screening as previously described.^10^ To engineer new PHL7 variants with enhanced thermostability properties, a heat treatment step at 75°C for 1 h was implemented prior to BHET hydrolysis reaction at 70°C. In the first round, the library was coarse screened on 20 mM BHET agar plates at 70°C after 6 h of reaction. Putative hits were then selected after fine screening on BHET agar plates at 20 mM and 40 mM concentrations at 70°C, up to 24 h reaction time (Figures 3A and 3E). Selected enzyme variants from the first round mostly contained single mutations, including N213I, R92L, E13V, and T29N, that displayed higher activity (larger clearing zones/halos) on BHET agar plates compared with PHL7-WT. These variants were then pooled together, along with the single-point mutation variants F63Y, L93F, L93Q, L93A, Q95S, Q95Y, Q95G, I179V, L210V, L210T, and L210F, obtained from the five site-saturation mutant libraries, as parents for the second round of directed evolution. To increase selection pressure for thermostability, enzyme libraries on BHET plates were heat treated at 75°C for 2 h before being incubated at 70°C for activity screening, with the coarse screening assay done at 40 mM BHET concentration, at 70°C for 6 h, and the fine screening assay performed on 40 mM and 60 mM BHET agar plates at 70°C and monitored for up to 24-h reaction time (Figures 3B and 3F).

The addition of a salt bridge in PHL7-WT (PHL7-Q175E/R205K) was found to increase both enzyme activity and thermostability, as shown in Figure S1 and discussed above, we therefore included this PHL7-Q175E/R205K variant along with the best eight variants obtained from round 2 as templates for DNA shuffling. Enzyme libraries on BHET plates were heat treated at 75°C for 2 h before being screened. Coarse screening was performed on 60 mM BHET agar plates, at 70°C for 16 h, with subsequent fine screening performed on 60 mM and 80 mM BHET agar plates at 70°C for up to 48 h. Validation assays were performed with normalized enzyme concentrations using cell lysates in reactions with 2.9% (w/v) amorphous PET film coupon loading (Figures 3C and 3G). The top 14 improved variants yielded from the third round were used as templates for DNA shuffling in the fourth round of directed evolution with coarse screening performed on 100 mM BHET agar plates at 70°C for 26 h and subsequent fine screening performed on 100 mM and 120 mM BHET agar plates with up to 48 h incubation at 70°C. Similar to previous rounds, enzyme libraries on BHET plates were heat treated at 75°C for 2 h prior to the BHET hydrolysis reaction at 70°C (Figures 3D and 3H). The thermostability of enzyme variants across the evolutionary trajectory, measured by amount of protein retained after heat treatment, is shown in Table S2.

After four rounds of directed evolution, four enzyme variants, which we named PHL7-Jemez, PHL7-Santa Fe, PHL7-Taos, and PHL7-Tusas (after four of the subranges of the Rocky Mountains in New Mexico, USA) that displayed more than 2-fold higher hydrolytic activity compared with PHL7-WT after 24 h of reaction with 2.9% (w/v) amorphous PET film coupons (Figures 3D and 3H) were selected for detailed characterization. The evolutionary trajectory of the enzyme variants is shown in Figure 3I with amino acid mutations of the four PHL7 enzyme variants along with PHL7 L93F/Q95Y, compared with the PHL7-WT scaffold, shown in Figure 3J. The four enzyme variants, PHL7-Jemez, PHL7-Santa Fe, PHL7-Taos, and PHL7-Tusas, were expressed, purified (Figure S2), and characterized alongside benchmark enzymes, PHL7-WT,^19^ LCC-ICCG,^6^ and PHL7-L93F/Q95Y.^17^ The DNA and amino acid sequences of the final four enzyme variants are given in Tables S3 and S4, respectively, with mutations highlighted in yellow. Expression yields from the expressed, purified enzymes in this study are shown in Table S5, while the relative expression for each variant across the evolutionary trajectory is shown in Figures 3E–3H. The DNA sequences and amino acid sequences for the best enzyme variants obtained along the evolutionary trajectory are shown in Tables S6 and S7, respectively.

To further explore if the mutations found in the PHL7 variants are present in homologous PET hydrolase sequences, we performed PSI-BLAST^27^ for the PHL7-WT sequence against the nr70 database using the MPI Bioinfomatics server.^28^ We looked at the top 250 hits, which showed sequence identities of 54%–75% with PHL7-WT and sequence coverages of 96%–100% (one sequence showed coverage of 91%). We specifically calculated the frequency of all mutations in the homologs of PHL7. We found a few mutations highly conserved evolutionarily, while a few others showed rare representation in the homologs. While R111H and T112I mutations were absent in the homologous sequences, R32P, T64S, Q95Y, H185N, and R205K appeared with a very low frequency in the homologs (frequency 0.4%–3.6%). The mutations that were strongly represented were A35V (46.8%), Q80H (23.2%), L93F (17.6%), and Q175E (58.4%). A graphical representation^29^ of the amino acid variations at 11 mutated sites in PHL7 homologous sequences is shown in Figure S3.

### Comparing engineered enzymes with benchmark PET hydrolases

The engineered enzyme variants were tested for activity on commercially available, amorphous (9.4% crystallinity^10^) PET film coupons (Goodfellow) at conditions at which PHL7-WT was initially reported to have the highest activity: 70°C, pH 8 in 1 M potassium phosphate buffer.^19^ To evaluate PET deconstruction, enzymes were introduced to reactions with PET substrate and monomer release (TPA, MHET, and BHET) was monitored over time using HPLC (Figure 3A). See Note S1 for discussion of BHET monomer quantification and Figure S4 for a comparison between UV absorbance and HPLC quantification of aromatic products. For activity testing, PHL7-WT and its variants were tested in reactions with PET in 1 M potassium phosphate buffer, its reported optimal conditions^17^^,^^19^ and verified independently in this study (Figure S5), while LCC-ICCG was tested in reactions with PET in 100 mM potassium phosphate buffer, based on its optimal conditions reported previously^6^ (the same optimal conditions as LCC-WT^30^) and verified independently here (Figure S6). PHL7 and its variants were not further characterized in reactions with PET in 100 mM phosphate buffer, as its activity is dramatically reduced at lower buffer concentrations than 1 M (Figure S5).

**Figure 4:**
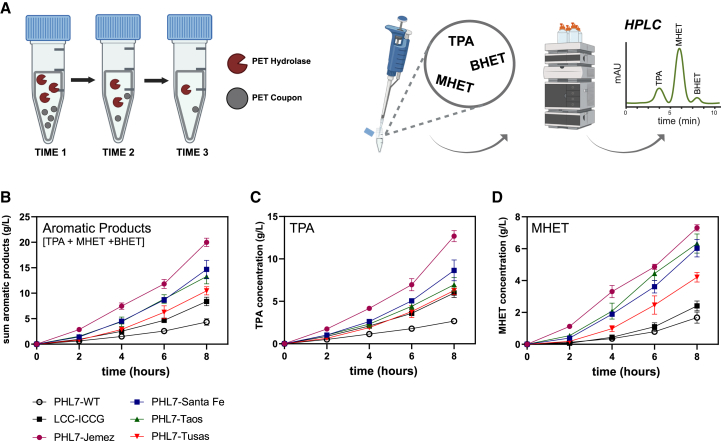
Initial enzymatic rates of PET hydrolases (A) Workflow for sampling PET reactions. Enzymes were added to reactions with PET, which were sampled over time. Samples were analyzed with HPLC, with monomers TPA, MHET, and BHET, and the sum of the three quantified. (B–D) Quantified products of reactions up to 8 h, with 0.7 mg enzyme/g PET, 2.9% (w/v) PET coupons, at 70°C and pH 8, for benchmarks PHL7-WT (white circles), LCC-ICCG (black squares), and four engineered PHL7 variants (colored shapes). Points display the average of *n* = 3 reactions, while error bars display ±1 SD. (B) Concentration of total aromatic products. (C) Concentration of TPA. (D) Concentration of MHET.

The engineered enzymes achieved higher initial rates of PET depolymerization compared with PHL7-WT, LCC-ICCG (Figures 4B–4D), and PHL7-L93F/Q95Y (Figure S7A), as well as higher (3-fold, on average) expression levels than PHL7-WT and comparable levels to LCC-ICCG (Table S4). The best-performing variant, PHL7-Jemez, achieved 2.4-fold higher conversion than LCC-ICCG by 8 h (on the basis of total aromatic products), and 4.6- and 7.2-fold higher conversions compared with PHL7-WT and PHL7-L93F/Q95Y, respectively (Figures 4B and S7A). Interestingly, much of the increase in total product release was due to an increase in MHET release (Figure 4D) by the PHL7 variants. Compared with the product release by LCC-ICCG, all four PHL7 enzyme variants showed 1.8- to 3-fold increases in MHET release, while TPA release was increased by ∼2.2-fold, with the highest observed for PHL7-Jemez (Figure 4C). Upon monitoring the pH of the reactions of PHL7-Jemez in 1 M buffer and LCC-ICCG in 100 mM buffer, we observed a greater pH change over a 48-h reaction for LCC-ICCG than PHL7-Jemez (Figure S8). However, as noted, LCC-ICCG still appears to have high activity even at comparatively lower buffer concentrations (Figure S6).

In initial reactions with 0.7 mg enzyme/g PET with 2.9% (w/v) amorphous PET film coupons, we observed that the substrates were fully depolymerized by 24 h in reactions with several of the PHL7 variants. To evaluate the enzyme activities over time, past 24 h, we decreased the enzyme loading to 0.35 mg enzyme/g PET, keeping the substrate concentration at 2.9% (w/v). The initial enzyme concentration of 0.7 mg enzyme/g PET (with 2.9% [w/v] PET) was selected to be in a similar range with other studies in the field, allowing for comparison. These studies include Erickson et al.,^4^ testing a number of natural, thermotolerant PET hydrolases (0.7 mg/g, with 2.9% [w/v] PET), and Pfaff et al.^17^ (0.5–2 mg/g, with one ∼2 × 1 cm PET coupon) and Richter et al.^16^ (0.55 mg/g, with one 3 × 0.5 cm PET coupon, ∼45 mg PET) when testing PHL7 and PHL7 mutants.

**Figure 5:**
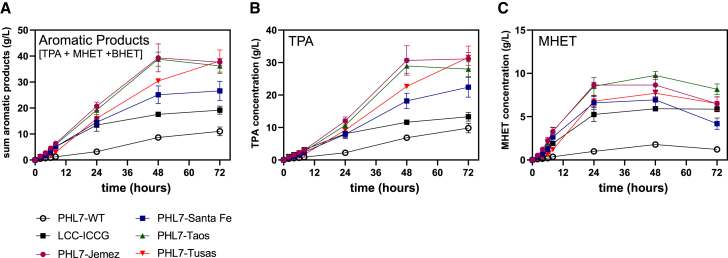
Depolymerization of amorphous PET film coupons as a function of time by PHL7 enzyme variants and LCC-ICCG Plots show products of reactions up to 72 h, quantified with HPLC, with 0.35 mg enzyme/g PET, 2.9% (w/v) amorphous PET film coupons, at 70°C and pH 8, for benchmarks PHL7-WT (white circles), LCC-ICCG (black squares), and four engineered PHL7 variants (colored shapes). Points display the average of *n* = 3 reactions, while error bars display ±1 SD. (A) Concentration of total aromatic products. (B) Concentration of TPA. (C) Concentration of MHET.

At lower enzyme concentration, the four engineered PHL7 variants again outperformed the benchmark enzymes (Figures 5 and S7B). The variants showed approximately 2-fold higher conversion by 72 h over LCC-ICCG, and 3.4- and 2.6-fold higher conversion than PHL7-WT and PHL7-L93F/Q95Y, respectively. In addition, we observed that the variants had higher hydrolytic activity on MHET compared with LCC-ICCG (Figure 5C), with MHET concentration depleting from its maximum concentrations (at ∼24 to 48 h) by the 72-h time point, whereas LCC-ICCG showed relatively constant MHET concentration beyond 24 h.

### Characterization of PHL7 variants at a range of reaction pHs and temperatures

We next characterized the enzymes beyond the standard testing conditions to evaluate their performance in several different reaction conditions. Enzymes were tested over a range of conditions: pH (pH 6, 7, 8, 9), temperature (65, 68, 70, and 72°C), PET substrate form and crystallinity (amorphous PET coupon [9.4% crystallinity],^10^ amorphous PET powder [13% crystallinity],^10^ and high crystallinity PET powder [41.8% crystallinity^31^]), and enzyme loading (0.35 and 0.7 mg enzyme/g PET).

Size reduction to increase the overall surface area of PET has been proposed as a way to enhance enzyme hydrolytic activity.^2^^,^^32^^,^^33^ However, we observed an overall decrease in hydrolytic activity when comparing amorphous PET film coupons with amorphous PET powder for the engineered variants (Figures 4, S9A–S9C, and S10A). Interestingly, the product released after 8 h of reaction for PHL7-WT, PHL7-L93F/Q95Y, and LCC-ICCG on the two amorphous substrates were similar, approximately 4 g/L product for PHL7 and PHL7-L93F/Q95Y and 8 g/L for LCC-ICCG. The PHL7 variants, however, showed significant decreases in conversion extent in reactions with the amorphous PET powder, with product releases reduced by roughly half, from ∼20 g/L to ∼10 g/L. This reduction in product release could be explained by the use of amorphous PET film coupons (not PET powder) as the main substrate to select for enzyme variants with enhanced hydrolysis performance throughout the directed evolution process. Here, higher hydrolysis refers to enhanced monomer yield, quantifying reactions with directly HPLC (or, with bioreactors, equivalents of OH^−^ required to neutralize reaction products). We further observed that while amorphous PET films were completely depolymerized in 24 h, the amorphous PET powder required extended reaction time, to 72 h, to achieve the same degree of depolymerization, potentially due to the PET substrate’s increased crystallinity from 9.4% to 13% upon being cryo-milled.^10^ We observed a similar reduction in hydrolytic activity in the case of the PHL7 variants toward high crystallinity PET powder (41.8% crystallinity,^31^ Goodfellow) (see Note S2).

**Figure 6:**
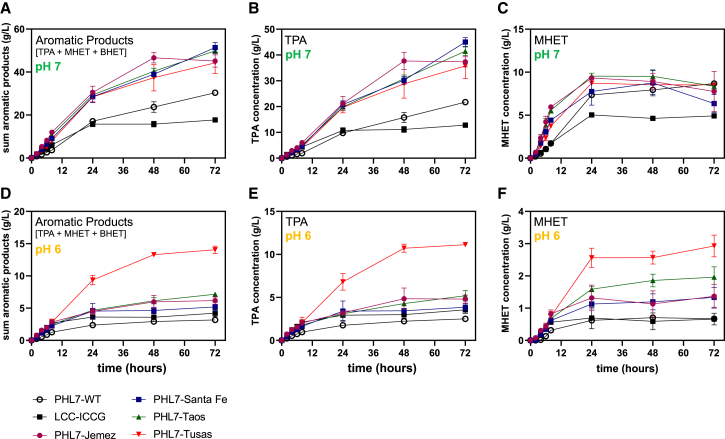
PET hydrolase activity at pH 6 and 7 Plots show products of reactions up to 72 h, quantified with HPLC, with 0.7 mg enzyme/g PET, 2.9% (w/v) amorphous PET film coupons, at 70°C, for benchmarks PHL7-WT (white circles), LCC-ICCG (black squares), and four engineered PHL7 variants (colored shapes) at pH 6 and pH 7. Points display the average of *n* = 3 reactions, while error bars display ±1 SD. (A–F) (A–C) Reactions at pH 7; (D–F) Reactions at pH 6. (A) Concentration of total aromatic products produced at pH 7. (B) Concentration of TPA produced at pH 7. (C) Concentration of MHET produced at pH 7. (D) Concentration of total aromatic products produced at pH 6. (E) Concentration of TPA produced at pH 6. (F) Concentration of MHET produced at pH 6.

Enzymatic performance at lower pH is an additional means to aid process viability,^34^^,^^35^ and thus, enzyme function at a pH lower than 8 is desirable. To this end, we investigated the performance of the PHL7 variants at lower pHs. Enzyme activities at pH 6 and pH 7 were reduced significantly compared with pH 8, up to 30%–40% less at pH 7 (Figures 4B–4D and 6A–6C) and 85% less at pH 6 (Figures 4B–4D and 6D–6F), in the first 8 h. Results at an increased pH 9 were similar to those at pH 8 (Note S3, Figure S11). At pH 7, our engineered enzymes again outperformed all benchmarks, with for instance, PHL7-Santa Fe showing 1.7- and 3-fold higher activities in 72 h than PHL7-WT and LCC-ICCG, comparing sums of aromatic products (Figure 6A). Additionally, we observed that at both pH 6 and pH 7, the hydrolytic activity of LCC-ICCG did not increase significantly beyond 24 h, whereas our variants, and PHL7-WT, continued to cause product release for the entire duration of the reaction. At pH 6, we observed dramatic decreases in hydrolytic activity for most of the enzymes, as well as the benchmark, LCC-ICCG (Figures 6D–6F and S7F). However, at pH 6, PHL7-Tusas showed significantly higher depolymerization, maintaining 27% of its productivity compared with pH 8 in the first 8 h, and reaching 100% of equivalent of productivity at pH 8 in 72 h (Figures 4B and 6D). Similarly, in comparison with the benchmark PHL7-L93F/Q95Y, PHL7-Jemez outperformed it under all four different pH conditions tested, ranging from pH 6 to pH 9 (Figure S7).

We further investigated the activities of the enzymes at different reaction temperatures. While our enzyme variants were engineered for improved depolymerization activity at 70°C, the objective was to compare their activities across a temperature range (65, 68, and 72°C) over which homologous thermotolerant enzymes have demonstrated activities,^4^^,^^5^^,^^6^ and which is still near the optimal temperature of enzymatic PET degradation, without reaching a temperature that could recrystallize the PET.^6^^,^^11^^,^^12^

Tournier et al. reported that incubating post-consumer PET at 72°C for ∼15 h increased its crystallinity from ∼15% to ∼40%, while it took less than 6 h at 75°C and over 24 h at 70°C.^6^ Still, Pfaff et al. tested their PHL7 variants activity at 72°C,^17^ a temperature at which LCC-ICCG was also reported to have high hydrolytic activity.^6^ While recrystallization occurs at higher temperatures, inhibition of the reaction by increased crystallinity may be kinetically outpaced by increased depolymerization activity: under different conditions, the speed of recrystallization can be less than or greater than the speed of depolymerization.^2^^,^^11^^,^^12^^,^^36^ Aside from temperature, this competition between depolymerization and recrystallization is affected by factors including enzyme loading, enzyme stability/deactivation, PET particle size or film thickness, polymer molecular weight, and amorphization/extrusion parameters, in the presence of water (or buffer).^11^^,^^36^^,^^37^ While the glass transition temperature, T_g_, of bulk PET has been reported to be approximately 65°C–81°C, the surface (interfacial) water-soaked T_g_ has been reported to be as low as 40°C^37^; however, these temperatures, may be too low for maximal enzyme activation and adhesion.^11^^,^^36^ As a result, the optimal reaction temperature, T_opt_, that balances the factors affecting depolymerization vs. recrystallization may vary based on the enzyme and process conditions.

Compared with activity at 70°C, at 65°C and 68°C, we saw, generally, that PHL7-WT and the PHL7 variants’ product releases were reduced from ∼20 g/L by 8 h to ∼15 g/L after 8 h (with 0.7 mg enzyme/g PET) and from ∼40 g/L by 72 h to ∼20 g/L after 72 h (with 0.35 mg enzyme/g PET) (Figures 4, S12, and S13). The variants’ catalytic activities at 65°C and 68°C were similar, with a trend of increasing activity up to 70°C. Meanwhile, the product released by LCC-ICCG remained relatively constant, about 10 g/L after 8 h and 20 g/L after 72 h (Figures S12 and S13). Consequently, below 70°C, LCC-ICCG outcompeted the exemplar PHL7 variant, PHL7-Jemez, but with the PHL7 variants performing better than LCC-ICCG as the temperature increased. In comparison to the benchmark PHL7-L93F/Q95Y, PHL7-Jemez showed 2- to 3-fold higher conversion at all three tested temperatures of 65, 68, and 70°C and at different enzyme concentrations (Figure S14). Despite having decreased initial rates at lower temperatures, one of our enzyme variants, PHL7-Tusas achieved the same level of conversion of LCC-ICCG by 72 h, with the product formed from this variant continuing to increase up to 72 h (Figure S12D). We did not observe this at 70°C, suggesting that, although the catalytic rate of the enzymes may decrease at lower temperatures, greater amounts of surviving enzymes may cause activity increases for longer times. Conversely, at 72°C (Figures S15A–S15C), all four PHL7 engineered variants had higher initial rates than those at 70°C (Figures 4B–4D), with all activities on par with PHL7-Jemez, the top-performing variant at 70°C. However, over time, the hydrolytic activity plateaued by 24 h for PHL7-Jemez and PHL7-Tusas (Figures S15D–S15F). We hypothesize this was due to increased reaction kinetics at 72°C, but decreased thermostability for the engineered enzymes, potentially also worsened by PET recrystallization.^6^^,^^11^^,^^12^^,^^37^ Therefore, 70°C appeared to be the optimal temperature for these enzymes, although interestingly PHL7-Taos showed higher product release (and apparent thermostability) at 72°C compared with itself and the other three variants at lower temperatures (Figure S15D).

### Evaluating product inhibition of PHL7-WT and PHL7-Jemez

Inhibition by product monomer accumulation is another key consideration in engineering PET hydrolases.^2^^,^^7^^,^^38^^,^^39^^,^^40^ Accumulation of TPA, EG, and MHET have been shown to affect the substrate hydrolysis rate of PET hydrolases,^38^^,^^40^ so we tested potential product inhibition using the top-performing variant, PHL7-Jemez, and PHL7-WT, by determining changes in monomer release in the presence of initial additions of TPA (Figures S16A and S16B), EG (Figures S16C and S16D), and MHET (Figures S16E and S16F). The concentrations of initial products were chosen to include a range similar to those used in previous studies by Barth et al. with *Tf*Cut2,^40^ Erickson et al. with *Is*PETase,^38^ and Tournier et al. with LCC-ICCG^6^: 0.5, 1.0, and 2.5 g/L of TPA, EG, and MHET. At high concentrations of TPA, PHL7-WT was observed to have a decrease in initial rate, which appeared to be relieved by the engineering of PHL7-Jemez (Figures S16A and S16B). With 2.5 g/L TPA addition, PHL7-WT suffered a 22% decrease in hydrolytic activity in 8 h compared with a reaction with no TPA added (*p* = 0.021, comparing 0.25% TPA and 0% TPA conditions), while PHL7-Jemez did not show a significant decrease (*p* = 0.29). No significant inhibition was observed by MHET and EG for both PHL7-WT (*p* = 0.36 for MHET; *p* = 0.054 for EG) and PHL7-Jemez (*p* = 0.29 for MHET; *p* = 0.23 for EG) in an 8-h reaction (Figures S16C–S16F), similar to *Is*PETase and *Is*PETase variants.^38^

### Structural evaluation of PHL7 mutations

**Figure 7:**
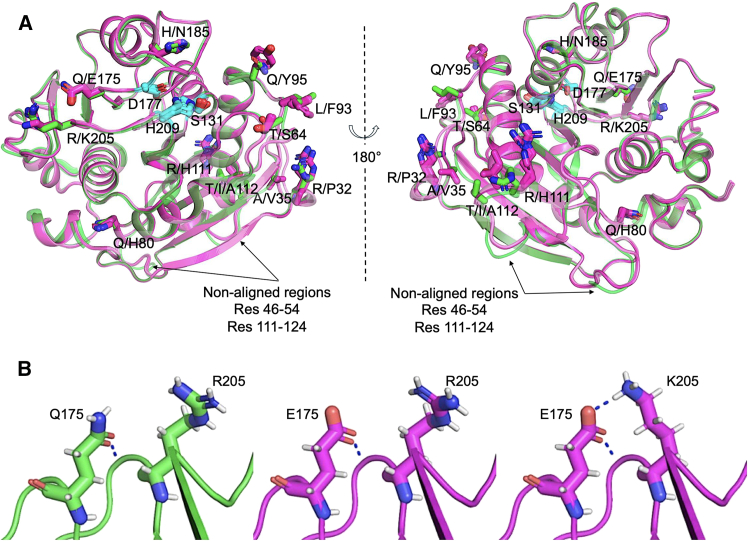
Structural modeling to gain insights into the engineered mutations of PHL7 (A) AlphaFold predicted structures of PHL7-Jemez (magenta) aligned to the crystal structure of PHL7-WT (green). The regions of maximum variability in the backbone were observed in the loops consisting of residues 46–54 and 111–124. Published sequence PHL7-L93F/Q95Y, was also modeled and overlayed on the crystal structure of PHL7-WT. The catalytic triad residues S131, D177, and H209 are highlighted in cyan. (B) ROSETTA modeling shows stabilizing effect of Q→E and R→K mutations at positions 175 and 205, respectively. A hydrogen bond is formed only in the case of the double mutation Q175E and R205K in PHL7-Santa Fe. The side chains of residues 175 and 205 are shown for PHL7-WT (left), PHL7-Jemez (middle), and PHL7-Santa Fe (right).

The roles of the various mutations in the PHL7 variants were investigated via structural modeling. The AlphaFold2 (AF2) structure prediction tool^41^ showed high confidence in each of the PHL7 variants’ modeled structures. PHL7-Jemez, PHL7-Santa Fe, PHL7-Taos, and PHL7-Tusas showed pLDDT scores of ∼98, while PHL7-L93F/Q95Y showed a pLDDT score of 96. Overlay of the predicted structures on the native PHL7 crystal structure (PDB code 7NEI)^19^ showed a root mean squared deviation (RMSD) of <0.6 Å over the full Cα backbone atoms (Figure 7A). The regions of maximum variation from the crystal structure included loops consisting of residues 46–54 and 111–124 that were also observed in the AF2-predicted structure of the native PHL7. To evaluate if the mutations enriched through directed evolution and co-screening of activity and expression were stabilizing, we performed computational modeling using ROSETTA.^42^ We used a design/relax protocol^43^ with an option to choose from the native and the identified mutations to determine if the observed mutations were stabilizing. We found the T64S mutation highly represented in the ROSETTA modeled sequences. Mutations such as Q80H and Q175E were also preferred in many models, indicating these mutations likely provide a stability advantage to the PHL7 variants.

Furthermore, various mutations were threaded on PHL7-WT structure to identify the role of each mutation. The Q175E mutation was predicted as most likely to contribute to the charge-charge interaction between the negatively charged E175 and positively charged R205 residue. The mutation, R205K showed an improved orientation and a formation of hydrogen bond between E175 and K205 (Figure 7B), a set of mutations that also showed an appreciable improvement in the stability of PHL7 protein (Figure S1B).

Comparing the thermostability of enzyme variants across the evolutionary trajectory, measured by the amount of protein retained after heat treatment in Table S2, it seems that A35V mutation in PHL7-Jemez decreases protein stability in low concentration buffers (100 mM potassium phosphate buffer, pH 8), which was confirmed by our computational modeling shown in Figure S17 and Table S8. However, ROSETTA modeling of the PHL7-WT and PHL7-Jemez variants shows the potential space-filling mutation A35V improves packing with F38 (Figure S18). Cartesian-ddG scores calculated using published protocols^44^ for each of the mutations present in PHL7 variants and the boxplot of ROSETTA computed scores for each of the PHL7 variant are shown in Table S9 and Figure S19, respectively.

**Figure 8:**
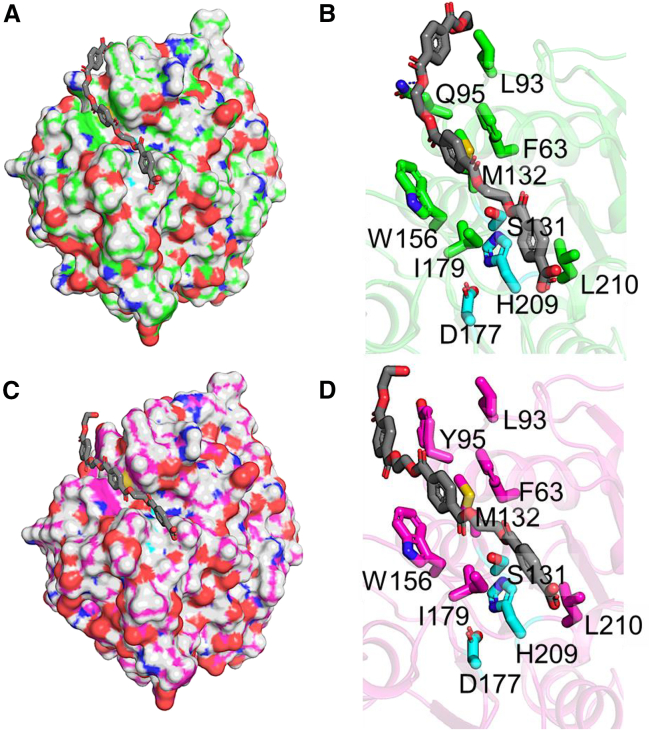
Computational docking of PET model substrate in the vicinity of the catalytic triad (A) Surface representation of PHL7-WT (green) with a bound PET model substrate (PET3mer). (B) PET3mer substrate can be divided into three PET units (units −2, −1, +1) where unit −2 is the leaving group resulting from the esterase activity. Key residues interacting with the PET3mer, that include L210/I179 packing against the leaving group (PET unit −2), W156/F63 pi-stacking against PET unit −1 and L93/Q95 packing against PET unit +1. In this pose, Q95 is also shown to form an H-bond with the ester linkage of PET unit +1. (C) Surface representation of PHL7-Jemez with a bound PET3mer. (D) Key residues interacting with the PET3mer include L210/I179 (PET unit −2), W156/F63 (PET unit −1), and Y95 pi-stacking against PET unit +1. Q95Y mutation in PHL7-Jemez results in a preference of an alternate binding mode for the PET3mer substrate, especially at the PET unit +1. The catalytic triad consists of S131, D177, and H209 (cyan).

To gain insight into what other catalytic features might be enhanced due to the mutations in PHL7-Jemez, we performed flexible docking of a PET model substrate consisting of three terephthalate and EG subunits (PET3mer) (Figure 8). Various conformers around the dihedral angles of PET3mer were created. Using a central point as the gamma oxygen of catalytic serine (S131), the PET3mer ligand was docked within a radius of 10 Å (Figures 8A and 8C).^45^ Top poses for both PHL7-WT (Figure 8B) and PHL7-Jemez (Figure 8D) showed comparable binding affinities, with W156 and F63 being key residues packing to the aromatic ring of TPA in the proximity of the scissile bond. The three PET3mer units (units −2, −1, +1)^6^ showed similarity in the binding mode at the PET unit −2 and −1, but the key difference was observed in PET unit +1, where the Q95Y mutation in PHL7-Jemez (Figure 8D), resulted in flipping outside the groove formed by L93/Q95 in the native protein, PHL7-WT (Figure 8B). While a similar groove is presented with L93/Y95 in PHL7-Jemez, the PET unit +1 aromatic ring in the substrate preferred pi-stacking only with Y95. To further understand the role of Y95 in the substrate recruitment and stabilizing the PET unit +1, we performed PET3mer docking in the published variant, PHL7-L93F/Q95Y.^17^ The top binding poses for PET3mer showed a preference or the mode that was more consistent with PHL7-Jemez substrate recruitment than PHL7-WT (Figure S20). The close proximity of two aromatic amino acids (F93/Y95) in the variant failed to provide a suitable interface for PET unit +1 of PET3mer. Whether such a difference in the substrate binding helps navigate different catalytic trajectories is worthwhile to be explored in a future study.

### Thermal denaturation kinetics of PHL7-WT and PHL7-Jemez

To further investigate the enzyme thermostability, we undertook a thermal denaturation study of both PHL7-WT and PHL7-Jemez by differential scanning calorimetry (DSC). We utilized multiple temperature scan rates to gain quantitative insights into the kinetics of enzyme unfolding, information that is unobtainable from a single scan rate that instead provides only an apparent T_m_. Although the highest PHL7-WT activity was previously reported in 1 M potassium phosphate buffer,^19^ here the PHL7 variants were investigated at lower (100 mM) phosphate concentration (Figure S21) because higher buffer concentrations were incompatible with this analysis, as the high ionic strength promoted sample precipitation within the DSC liquid handling system.

For both enzymes, the DSC thermogram changed significantly as the scan rate increased from 0.2°C to 3.2°C/min, with the endotherm sharpening, and the apparent T_m_ (the maximum on the DSC thermogram) rising by over 7°C, from 73.9°C to 81.2°C for PHL7-WT, and from 72.4°C to 79.9°C for PHL7-Jemez (Figures S21A and S21E). These observations are not consistent with either a reversible denaturation with rapid exchange between folded and unfolded states (for which the apparent T_m_ would be independent of scan rate), or a single-step, irreversible denaturation mechanism (for which the endotherm shape would remain constant). Instead, for both enzymes, a two-step, irreversible denaturation (i.e., from the native state through an intermediate to the denatured state) is the simplest model that gives a satisfactory fit to the experimental data (Figure S22). In this model, each transition (either native-to-intermediate or intermediate-to-denatured) is characterized by a calorimetric enthalpy (ΔH_cal_), an activation energy (*E*_a_), and a reference temperature (*T∗*) at which one enzyme molecule per second transits. Mathematical modeling of the sets of thermograms acquired with differing scan rates provides estimates of these energetic parameters for PHL7-WT and PHL7-Jemez (Table S10). From deconvoluting the thermograms into the two discrete transitions (Figures S21C and S21G) and comparing the magnitude of their respective ΔH_cal_ values, it is clear that the observed DSC endotherm is dominated by the native-to-intermediate transition for both PHL7 variants.

The 0.3°C–0.5°C lower apparent T_m_ values for PHL7-Jemez suggests this engineered variant has a marginally lower thermostability than PHL7-WT. To quantify this, we utilized the *E*_a_ and *T*^∗^ parameters to model the rate constants for native-to-intermediate (*k*_1_) and intermediate-to-denatured (*k*_2_) transitions across the temperature range of interest (Figure S22). We assume that, as with the denatured state, the intermediate state has no catalytic activity, hence *k*_1_ is of more functional significance than *k*_2_. For both variants, within the range of 65°C–72°C (the conditions for the PET hydrolysis activity measurements), *k*_1_ increases and, due to its inverse proportionality, the native state lifetime decreases by ∼44% with each 1°C rise in temperature. Notably, *k*_1_ for PHL7-Jemez is consistently ∼40% higher than for PHL7-WT in this temperature range, implying a reduced kinetic stability of this engineered variant. Indeed, under these low buffer concentration conditions, our modeling suggests that, at 65°C (the temperature selected for the bioreactor-scale PET hydrolysis experiments), the *k*_1_ rate constant is 6.0 × 10^−5^ s^−1^ for PHL7-WT, and 8.5 × 10^−5^ s^−1^ for PHL7-Jemez, corresponding to native-state enzyme half-lives of 3.2 and 2.3 h, respectively. A higher phosphate buffer concentration may extend these half-lives, given the previously observed 5°C increase in apparent T_m_ for PHL7-WT on raising the phosphate buffer concentration from 50 mM to 1 M.^19^

### PET hydrolysis using PHL7 variants in bioreactors

**Figure 9:**
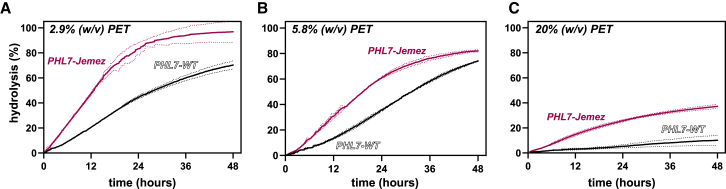
PET hydrolysis by PHL7-WT and PHL7-Jemez in bioreactors PHL7-WT (black) and PHL7-Jemez (magenta) were added to reactions of amorphous PET coupons (1 mg enzyme/g PET) in bioreactors at 65°C with 1 M potassium phosphate buffer, pH 8. Bioreactors were monitored for hydrolysis of PET over a 48-h reaction, with total mass loss observed at the end of the reaction (boxed). Data points and percentages are average of *n* = 2 bioreactors, while dotted lines represent ±1 SD. PET coupon solids loading was varied. (A–C) (A) Reaction with 2.9% (w/v) PET. (B) Reaction with 5.8% (w/v) PET. (C) Reaction with 20% (w/v) PET.

To demonstrate the utility of the newly engineered enzymes in bioreactors, which better represent industrial recycling processes,^2^^,^^7^^,^^35^^,^^46^ we evaluated PET deconstruction by PHL7-Jemez and PHL7-WT in bioreactors (Figure 9). The extent of PET hydrolysis over time and final solids mass loss were determined for reactions with PHL7-Jemez and PHL7-WT with amorphous PET film coupons (Figures 9A–9C). Additionally, PET solids loading was varied to determine its effects on enzyme performance: 2.9% (w/v) was used to mirror small-scale reactions (Figure 9A), which was also doubled to 5.8% (w/v) (Figure 9B), and 20% (w/v) was used to mimic levels similar to what would be used industrially (Figure 9C).^7^

The comparative performance of PHL7-Jemez in bioreactors was similar to that in small-scale reactions, showing improvement over the wild-type enzyme (Figures 9A–9C). PHL7-Jemez had approximately 2.5-fold higher initial rates (hydrolysis up to 12 h) at low PET loadings (2.9% and 5.8%) than PHL7-WT (Figures 9A and 9B) and nearly 5-fold higher initial rates at high PET loadings (20%) (Figure 9C). This higher catalytic activity was maintained over time for all conditions, with, for example, nearly 95% hydrolysis in 36 h by PHL7-Jemez compared with 60% by PHL7-WT (at 2.9% PET) (Figure 9A). PHL7-Jemez, at all conditions, also showed the highest polymer mass losses, with nearly 96.5% at 2.9% (w/v), 97.5% at 5.8% (w/v), and 70.5% at 20% (w/v) PET loading (Table 2). Interestingly, however, with increasing PET loadings, we saw diminished hydrolysis by PHL7-Jemez, starting at 5.8% PET, where only 82% hydrolysis was seen in 48 h, compared with almost 97% at 2.9% PET, even while maintaining approximately the same polymer mass loss, ∼97% (Figures 9A and 9B; Table 2). Reaction extent was severely affected at 20% PET loading, with only 37% hydrolysis and 70.5% mass loss in 48 h by PHL7-Jemez (Figure 9C; Table 2); the cause of this discrepancy between these two measurements is unclear. Nevertheless, by both means of quantitating progress, this is about a 3.5-fold higher reaction extent compared with PHL7-WT, at industrially relevant conditions.

**Table 2:** Mass loss of PET for bioreactor runs

PET loading (% [w/v])	Mass loss (%)	
	PHL7-WT	PHL7-Jemez
2.9	87.0 ± 0	96.5 ± 0.5
5.8	84.5 ± 2.5	97.5 ± 0.5
20	19.5 ± 5.5	70.5 ± 0.5

For PHL7-WT and PHL7-Jemez, PET coupons (1 mg enzyme/g PET) were deconstructed in bioreactors at 65°C with 1 M potassium phosphate buffer pH 8. Mass loss was determined after 48 h reactions. Measurements are of *n* = 2 bioreactors, with ±1 SD shown.

We hypothesize that the diminished hydrolysis at higher PET loadings, along with the discrepancies between hydrolysis and mass loss in reactions, were caused by the high reaction buffer concentration. When employing 1 M phosphate buffer, the ionic strength begins high and further increases as (Na^1+^)_2_TPA^2−^ is generated, resulting in the extensive precipitation witnessed in the reaction vessels, which is likely sodium phosphate salts but may also contain enzyme and monomer products.

## Discussion

Here we present the engineering of a thermotolerant PET hydrolase enzyme, PHL7, combining rational design and directed evolution using HT screening.^10^ At the conditions tested, the four engineered enzyme variants reported here outperformed its parent, PHL7-WT. These enzymes were evolved with significantly enhanced initial catalytic rates, along with higher hydrolytic activities over time, through rational/semi-rational design and random mutagenesis. This work, in addition to providing a new set of high-performance PET hydrolases, further demonstrates the utility of our PET hydrolase engineering platform toward improved enzymatic PET degradation and recycling. Comparing with the benchmark, LCC-ICCG (at its optimal buffer condition of 100 mM phosphate), the engineered PHL7 variants reported here (at their optimal buffer condition of 1 M phosphate), outperformed LCC-ICCG in reactions with 2.9% (w/v) amorphous PET film coupons at 70°C, under different pH conditions ranging from pH 6 to pH 9. The engineered PHL7 variants also outperformed LCC-ICCG in reaction with 2.9% (w/v) amorphous PET powder at 70°C, pH 8. However, at temperatures below 70°C and in reactions with high crystallinity PET powder, LCC-ICCG outperformed PHL7 variants. The high buffer concentration required for higher hydrolytic activity of PHL7 enzyme variants might help to better stabilize the pHs of the reactions and contribute to how PHL7 enzyme variants outperformed LCC-ICCG in small-scale reactions.

In reactions with amorphous PET film coupon loadings ranging from 2.9% to 20%, PHL7-Jemez exhibited higher extents of hydrolysis and mass loss compared with PHL7-WT, which demonstrates the catalytic enhancement of PHL7-Jemez compared with the parent enzyme, underscoring the utility of our HT screening platform for PET hydrolase engineering. In comparison with other, established, PET hydrolase benchmarks in bioreactors including LCC-LANL^10^ and LCC-ICCG^6^ (Figure S23), at 2.9% amorphous PET loading and similar reaction conditions (pH 8, 65°C), LCC-ICCG (in 100 mM phosphate buffer) showed up to 65% hydrolysis in 36 h and 83.5% mass loss in 48 h,^10^ compared here with PHL7-Jemez (in 1 M phosphate buffer) with 94% hydrolysis and 96.5% weight loss. Compared with LCC-LANL^10^ (in 100 mM phosphate buffer), PHL7-Jemez had significantly improved catalytic rates, with 36% higher hydrolysis extent in 24 h, although with similar overall extents of reaction (90% and 95% hydrolysis in 48 h) and overall mass loss (94.5% and 96.5% loss), respectively.

However, while PHL7-Jemez may be a high-performance PET hydrolase enzyme, at higher PET loadings, it appears that PHL7-Jemez would not outperform LCC-ICCG, which achieved over 60% hydrolysis of 16.5% (w/v) PET coupons in 48 h in our previous report,^10^ compared here with less than 40% hydrolysis of 20% (w/v) PET in 48 h. In addition, at the high buffer concentration tested here, PHL7-Jemez is not yet suitable to be used industrially. The high buffer concentrations used here for PHL7-WT and its engineered variants (and some other enzymes^47^) would cause significant process and separation challenges, beginning with the reaction inhibition we experienced, but also causing issues with, e.g., product separation, as acidification to precipitate TPA may not be possible in high concentration buffers, or may be prohibitively costly.^34^^,^^35^

To address potential issues caused by high buffer concentration, as a start, buffer concentration can reportedly be decreased to moderate amounts without significant decreases in activity^17^ or this may require future protein engineering efforts. The HT screening platform used here can be adapted for directed evolution of PET hydrolases with process-relevant variables as selection pressures. Reactor design may also contribute to alleviating issues posed by buffer concentration. For instance, enzyme membrane reactors could allow *in situ* removal of product monomers while maintaining high conversions.^48^^,^^49^ Using a moist-solids reaction may be of particular advantage to our variants, allowing them to deconstruct PET substrates efficiently, while removing buffer constraints. Kaabel et al. studied enzyme activity in moist-solids reactors, where minimal amounts of buffer were used, and observed no significant change in activity resulting from different buffer concentrations.^50^

In reactions at 70°C (Figures 4 and 5), a key contributor to the high hydrolytic activity of our enzyme variants was their ability to liberate large quantities of MHET in comparison with LCC-ICCG and PHL7-WT. It appeared that the MHET hydrolysis activity of PHL7 and its variants was particularly affected by lower temperatures, further supported by what appeared to be a decrease in ability to convert MHET to TPA, even over a 72-h reaction, at 65°C (Figure S12F). Higher temperatures could be requisite for high TPA production. Taken together, it appears that PHL7-WT and its engineered enzyme variants are substantially affected by temperature, more so than LCC-ICCG. This may be explained by an increased reliance on a more accessible polymer structure as the temperature was increased, and/or inherent temperature preference reinforced by evolution, possibly suggested by a decrease in MHET hydrolysis activity. Interestingly, PHL7-Tusas had among the highest activity, post-8 h reaction, at lower temperatures, appearing to perform better in relation to the other PHL7 enzyme variants at these lower temperatures than at 70°C. In addition, PHL7-Tusas had the highest performance at lower pH (pH 6), indicating that PHL7-Tusas could be used as a starting point for further engineering for higher activities at a range of temperatures, and/or under acidic pHs if desired, promoting more economical and industrially impactful PET recycling. This is a potential advantage of our screening platform, wherein enzyme variants can be screened for higher activity under certain selection pressures, including gradual lowering of reaction pH.

Our screening platform also could be adapted to discover enzyme variants that are more active toward high crystallinity PET substrates. While our engineered variants here exhibited enhanced activity toward high crystallinity PET substrate (Goodfellow high crystallinity PET powder) compared with the starting template, PHL7-WT, LCC-ICCG still performed significantly better than all other variants tested on that substrate. However, PHL7-Jemez, for example, could be used as a starting point for a directed evolution campaign with improved activity on high crystallinity PET as a selection criterion. PET hydrolases with enhanced activity on high crystallinity PET would be of particularly advantageous for improving process economics as, currently, high crystallinity PET substrates are more resistant to depolymerization by enzymes^2^^,^^4^^,^^6^^,^^50^ and substrate pre-treatment steps present a significant cost barrier in industrial PET recycling by enzymes.^34^^,^^35^ One of the potential limitations in our study is that we did not explicitly test the independent effects of PET molecular weight, which has recently been suggested by Pfaff et al.^17^ and Cui et al.^51^ to be an additional factor in enzymatic degradability. Cui et al.,^51^ however, observed small decreases in PET molecular weight over the course of reactions and posited that comparatively greater enzyme activity on higher crystallinity PET substrates could be explained by lower molecular weights, while Pfaff et al.^17^ suggested that PHL7-WT’s activity was more affected by percent crystallinity, and LCC-ICCG’s (which may more efficiently hydrolyze shorter polymers) was more affected by molecular weight. While this study used substrates standard to the field (Goodfellow amorphous PET film, Goodfellow high crystallinity PET powder, crushed/milled Goodfellow amorphous PET powder), that have been characterized for molecular weight by Pfaff et al.^17^ and Cui et al.,^51^ we recognize that PET substrate properties may vary and that the reported effects of molecular weight on hydrolytic activity warrants future investigation.

The mutations found in the engineered PHL7-Jemez appeared to relieve TPA product inhibition observed for PHL7-WT (Figures S16A and S16B). No significant inhibition was observed by MHET and EG for both PHL7-WT and PHL7-Jemez (Figures S16C–S16F), suggesting that PHL7 enzymes are able to tolerate high MHET concentrations. LCC-ICCG was likewise not shown to experience product inhibition by MHET, EG, and TPA,^6^ with its primary limitation explained by thermal degradation of the protein. We posit the same is true for PHL7 and its variants. While PHL7-Jemez demonstrated higher catalytic activity than PHL7-WT both in small scale and in pH-controlled bioreactors at the conditions tested (1 M potassium phosphate buffer), the mutations in PHL7-Jemez appeared to reduce native-state enzyme half-lives in 100 mM potassium phosphate buffer concentrations. The use of high phosphate buffer concentrations throughout the screening/selection process may have increased enzyme thermostability, thereby, making improvement in activity and expression/solubility the two main criteria for selection. The engineered salt bridge presented in PHL7-Jemez may increase enzyme thermostability; however, other mutations to PHL7-Jemez may increase activity at the detriment of stability. In high (1 M) phosphate buffer concentration reactions, at which the variants were engineered and tested, the loss in enzyme thermostability was compensated by an increase in apparent T_m_ as a result of the high buffer concentration employed.^19^^,^^52^

Mutations from the enhanced PHL7 enzyme variants presented here are added to a growing catalog of mutational hotspots for PET hydrolase engineering. For PHL7, a mutation at H185 may enable an increase in enzyme activity compared with the wild type, as supported by previous studies on homologs.^10^^,^^23^^,^^53^^,^^54^ While positions previously identified as impactful for function may be targeted through traditional rational design strategies, these efforts may still fail to precisely identify optimal mutations at these positions.^16^ Here, we discovered the beneficial mutation H185N through random mutagenesis, while Richter et al. reported that the predicted mutation H185S (mapped from a beneficial mutation in *Is*PETase^53^) decreased stability and activity.^16^ This study highlights the advantage of our screening platform through screening large, random mutagenesis libraries, as we have successfully discovered mutations at previously flagged positions where other, rational design studies have failed. Interestingly, both Cribari et al.^54^ and Groseclose et al.^10^ recently discovered the beneficial mutation H218Y in LCC-ICCG,^54^ which is an analogous site of PHL7 H185. Further, the datasets obtained throughout the process of directed evolution via screening large libraries using our workflow (simultaneously quantifying expression/solubility and activity, at specific conditions) may provide training data for artificial intelligence/machine learning models, further accelerating discovery of high-performance PET hydrolases beyond the HT-directed evolution platform demonstrated here.

## Methods

### Materials and data analysis

Unless noted, materials were obtained from the following sources. Oligonucleotides were purchased from Integrated DNA Technologies. Genes were synthesized by Twist Biosciences. Sanger sequencing was performed by Genewiz. Enzymes were purchased from New England Biolabs. Amorphous PET films (product ES301445; 9.4% crystallinity^10^) and high crystallinity PET powder (product ES306031; 41.8% crystallinity^31^) were purchased from Goodfellow Cambridge Ltd. A micronized amorphous powder was produced from the PET film by cryo-milling, first in an SM300 cutting mill (Retsch), then in a ZM200 centrifugal mill (Retsch), as described previously,^4^ but using a ring sieve with a larger pore size (0.5 mm) in the second step. The powder was thoroughly dried at 45°C for over 24 h before use as a substrate. The crystallinity was determined based on DSC, either by Groseclose et al.^10^ (amorphous film and powder) or Cuthbertson et al.^31^ (crystalline powder). Chemicals were purchased from Fisher Scientific or Millipore Sigma. Kits were purchased from Qiagen. Data analysis and curation were performed in Microsoft Excel, GraphPad Prism, and Agilent OpenLab CDS. Sequencing and gene design were performed using ApE (M. Wayne Davis) and SnapGene (Dotmatics). Figures were prepared with Adobe Illustrator and BioRender.

### Cloning, mutagenesis, and library creation

A codon-optimized, synthesized gene encoding PHL7-WT^19^ was cloned into the pET21b(+)-GFP11 screening vector,^10^ between the *Nde*I and *Bam*HI sites. The DNA sequence of this construct is shown in Table S1. A plasmid encoding LCC-ICCG in the pET21b(+) vector was used from previous reports.^4^ A synthesized gene encoding PHL7-L93F/Q95Y^17^ was cloned into the pET21b(+) vector between the *Nde*I and *Xho*I sites. The DNA and protein sequences of these variants are shown in Tables S3 and S4. Selected engineered variants were amplified from the pET21b(+)-GFP11 vector with *Nde*I and *Xho*I sites and subcloned into pET21b(+), as necessary, for expression and purification using the His_6_ system. Plasmids were transformed into *E. coli* BL21 (DE3) Gold cells (B F^−^ *ompT hsdS*(rB^−^ mB^−^) *dcm*^+^ Tet^r^ gal λ(DE3) *endA* Hte). Chemical transformation was used for routine cloning, while library transformations used in-house electrocompetent cells. Cells were cultured either using LB Miller agar or LB Miller liquid media, with relevant antibiotics (carbenicillin, 100 μg mL^−1^).

Site-directed mutagenesis (SDM) was performed by inverse PCRs using 5′-phosphorlyated oligo primers, followed by treatment with DpnI and T4 DNA Ligase at 30°C overnight. Site-saturation mutagenesis (SSM) was performed in the same way, except with the use of degenerate (NNK) oligos at the position of interest. For SDM, single colonies were picked, cultured, subjected to plasmid isolation, and sequence verified. For SSM, 96 colonies were picked for screening into media in a 96-well plate.

Libraries were constructed using a DNA shuffling protocol adapted from Waldo^55^ and our previous report.^10^ Briefly, gene templates were amplified by Q5 DNA Polymerase (NEB), then fragmented with DNAseI (Invitrogen). Fragmented DNA was re-assembled and amplified using Exo(−) Pfu DNA Polymerase (Agilent). Full-length library gene fragments were cloned into pET21b(+)-GFP11 between the *Nde*I and *Bam*HI sites, after digestion with restriction enzymes and ligation with T4 DNA Ligase. Sequencing after ligation revealed that each 780-base pair (bp) gene incurred 1–3 bp random mutations per round, accounting for 1–3 residue mutations, with up to 2 (generally 0 to 1) silent mutations. The ligated library was transformed into *E. coli* cells, which was selected for on LB Miller plates with carbenicillin. Colonies on plates were streaked into LB liquid media, were prepared as 1.0 OD_600_ glycerol stocks, and were stored at −80°C until use.

### HT co-screening assay

The protocol was adapted from our previous report.^10^ Briefly, transformed bacterial libraries were plated on Durapore PVDF 0.45-μm 47-mm membrane filters (product HVLP14250) on LB agar plates. To yield a well-spread, yet pickable density of cells on the plate, libraries were plated at approximately a 2.5 × 10^5^ dilution from a 1.0 OD_600_ freezer cell stock. Library plates were then grown overnight. The next day, Durapore membranes (with cells) were transferred onto LB agar plates with IPTG (1 mM) and incubated for 2 h to induce protein expression. Membranes were then transferred to BHET screening plates. To cast BHET screening plates, first, a 0.7% (w/v) agarose in (500 mM potassium phosphate pH 8) buffer solution was made. BHET solution (at a working concentration of 500 mM BHET in 100% DMSO) was then added to the agarose solution (in 50-mL total aliquots) to the appropriate concentration (ranging from 20 to 120 mM BHET), then mixed well, pouring into a 50-mm Petri dish, then cooled; 500 mM buffer was used in screening plates due to solubility limitations of agarose at 1 M buffers.

For coarse screening, library colonies were lysed on screening plates by spraying membranes with BugBuster (Millipore) two to three times from a spray bottle, rotating the plate. This method ensures an even coverage of BugBuster and lysed cells across the plate, as shown in our previous report.^10^ Membranes were then removed from plates and stored at 4°C on original LB agar plates. Screening plates were then incubated at relevant heat treatment and screening temperatures. Incubations and reactions were done in VWR Hybridization Ovens (model 5420), for 2 to 24 h. After reactions were completed, solutions of refolded GFP1-10 in (100 mM Tris-HCl pH 7.4, 150 mM NaCl, 10% [v/v] glycerol) TNG buffer were put on screening plates. GFP1-10 was refolded from inclusion bodies as in previous reports^24^^,^^56^ then incubated 4 h to overnight. Plates were imaged using a ChemiDoc MP Imager, detecting colorimetric blot and Alexa 488 signals. Membranes (with partially lysed colonies) were then re-aligned on screening plates and colonies were picked into LB in 96-well plates for next steps of screening.

For fine screening, colonies with improved performance selected from libraries were grown out in plates overnight, then replica plated (Boekel Scientific) onto Durapore membranes. The screening process was repeated as above, but with 8 μL of BugBuster pipetted onto each colony for cell lysis. Colonies selected from this fine screening were chosen as putative improved variants for our validation screening assay using real PET substrates.

### Validation screening assay

Putative improved variants were expressed in small-scale, 2- to 25-mL expressions. Starter cultures of colonies grown overnight in LB were inoculated 1:100 into 2 to 25 mL of 2xYT media with antibiotic in Falcon tubes (Fisher Scientific) or deep-well microwell plates (USA Scientific) and grown to 0.6 to 0.8 OD_600_ at 37°C, 250 rpm. Cultures were then placed on ice or at 4°C for 10 min before 1 mM IPTG was added to induce expression, which were then grown for an additional 16–20 h at 20°C, 150 rpm. Cultures were pelleted at 3,500 rpm for 20 min, supernatant was removed, and pellets were resuspended in 500 μL of lysis buffer (100 mM potassium phosphate pH 8, 200 mM NaCl) then lysed by sonication with a Fisherbrand Model 50 Sonic Dismembrator (Fisher Scientific). Sonication was 5 × 20 s, on ice, centrifuging at 14,000 rpm for 3 min at 4°C between cycles, with a final centrifuge for 30 min to clarify cell lysate.

Enzyme concentration in cell lysates was measured via plate reader (detecting GFP fluorescence intensity; excitation: 488 nm, emission: 520 nm) after complementation with GFP1-10. Briefly, 20 μL of cell lysate was added to Corning MaxiSorp 96-well plates with 180 μL of refolded GFP1-10 in TNG buffer. Plates were then incubated overnight at room temperature with shaking. Proteins were quantified via a standard curve from 2-fold serial dilutions of a purified sulfide reductase-GFP11 construct (from 0.11 to 14.26 μM; see Groseclose et al.^10^ and Cabantous et al.^24^). Background fluorescence was subtracted from all samples using the cell lysate of an expression construct lacking the GFP11 tag (i.e., PHL7 in pET21b[+]). Fluorescence was measured using a Tecan M Plex Plate Reader. GFP1-10 complementation was performed in triplicate.

Proteins were diluted to 0.5 or 1 μM using lysis buffer and added 1:10 (to 0.05 μM, in 500 μL total) in reactions containing 1 M potassium phosphate buffer, pH 8 (PHL7) or 100 mM potassium phosphate buffer, pH 8 (LCC-ICCG) reaction buffer, and 0.92% (w/v) PET coupons as 3-mm hole-punched circles (approximately 2.5 mg each; Fiskars). Reactions were then incubated in deep-well 96-well plates at 70°C, with aliquots drawn at each time point: 2, 4, 6, 8, 24, 48, and 72 h. Absorbance at 240 nm was measured using a Tecan M Plex Plate Reader to detect aggregate aromatic products released,^25^ with baseline (t = 0) absorbance for each enzyme subtracted from time points. BHET equivalent concentrations were determined from a standard curve of absorbance of serially diluted BHET. Promising enzyme variants were grown out and plasmids were isolated and sequenced. Plasmids from any promising variants were used as parents for additional rounds of evolution.

### Directed evolution of PHL7

DNA fragments encoding for PHL7-WT were used as starting templates to create random mutation libraries, cloned into the pET21b(+)-GFP11 vector, and transformed into *E. coli* as described above. To engineer new PHL7 variants with enhanced thermostability properties, enzyme libraries on BHET plates were heat treated at 75°C for 1 h before being incubated at 70°C for colony screening. An HT co-screening assay with coarse screening was first performed, followed by fine screening. In the first round, the library was coarse screened on 20 mM BHET agar plates at 70°C after 6 h of reaction after heat treatment for 1 h. Colonies that displayed higher BHET hydrolytic activity (larger clearing zones) and/or higher enzyme solubility (brighter green fluorescence) were picked and grown in 96-well plate format as previously described. Putative hits were then selected after fine screening on BHET agar plates at 20 mM and 40 mM concentrations at 70°C, up to 24-h reaction time, after heat treatment for 1 h. After a validation assay using normalized concentrations of enzymes in cell lysates (using 0.92% [w/v] PET coupons), the top four enzyme variants selected were then pooled together with the best 11 single-point mutation variants obtained from the five site-saturation mutant libraries as parents for the second round of directed evolution, with the coarse screening assay done at 40 mM BHET concentration, at 70°C for 6 h, and the fine screening assay performed on 40 mM and 60 mM BHET agar plates at 70°C and monitored for up to 24 h reaction time. Similar to the first round, enzyme libraries on BHET plates were heat treated at 75°C for 2 h before being incubated at 70°C for colony screening. Validation assays were performed for enzyme variants selected from the second round along with the starting template PHL7-WT (also with 0.92% [w/v] PET loading).

The third round of evolution was performed using the best eight variants obtained from round 2 and the Q175E/R205K salt bridge variant as templates. Coarse screening was performed on 60 mM BHET agar plates, at 70°C for 16 h, with subsequent fine screening performed on 60 mM and 80 mM BHET agar plates at 70°C for up to 48 h. Enzyme libraries on BHET plates were heat treated at 75°C for 2 h before being screened. Validation assays were performed with normalized concentrations of cell lysates with 2.9% (w/v) PET loading.

In the final round of directed evolution, the top 14 improved variants yielded from the third round were used as temples, enzyme libraries on BHET plates were heat treated at 75°C for 2 h prior to the BHET hydrolysis reaction at 70°C. BHET concentrations were also increased to 100 mM in coarse screening with a longer reaction time of 26 h. Fine screening was performed with both 100 mM and 120 mM BHET agar plates with up to 48 h incubation at 70°C. After the fourth round of directed evolution, four PHL variants: PHL7-Jemez, PHL7-Santa Fe, PHL7-Taos, and PHL7-Tusas were selected for final characterization, after a validation assay using enzymes in cell lysates with 2.9% (w/v) PET.

### Protein expression and purification

Proteins were expressed using the pET21b(+) expression vector, using His_6_ tag purification with Co TALON Resin (Takara Bio). Colonies were streaked out on LB selection plates, picked, and grown out overnight in LB media at 37°C, 250 rpm. Cultures were then inoculated 1:100 into 500 mL 2XYT media with carbenicillin, grown to 0.6 to 0.8 OD_600_ at 37°C, 250 rpm, and induced with 1 mM IPTG after being cooled for 10 min on ice or at 4°C. Cultures were then grown for an additional 16–20 h at 20°C, 150 rpm. Cells were harvested for 20 min at 3,500 rpm and stored at −80°C until purification.

For purification, pellets were thawed and resuspended in 30 mL column buffer (100 mM potassium phosphate pH 8, 200 mM NaCl, 10% [v/v] glycerol), then sonicated using a Branson Digital Sonifier 450 at 80% amplitude for 10 min on ice at 20°C. Lysate was clarified by centrifuging 1 h at 4°C and 40,000 × *g*, then filtered with a 0.45-μm syringe filter before loading onto 2.5 mL packed, equilibrated resin. The lysate was incubated with the resin, rocking at 4°C overnight. Purification was performed manually. Flow-through was discarded and the resin was washed with 15 column volumes (CVs) of column buffer (100 mM potassium phosphate pH 8, 200 mM NaCl, 10% [v/v] glycerol), 10 CVs of column buffer with 5 mM imidazole, and finally eluted with 5 CVs with column buffer with 250 mM imidazole. Proteins were verified for correct size and purity by SDS-PAGE by running alongside Protein Kaleidoscope Protein Standards (Bio-Rad). Purified protein samples were boiled in Laemmli Buffer at 100°C for 20 min before loading on a gel. Purity of purified proteins was >90% (evaluated with Image Lab, Bio-Rad). Enzymes were then buffer exchanged using an Amicon 10-kDa cutoff filter (Millipore Sigma) with 100 mM potassium phosphate pH 8, 200 mM NaCl, using the manufacturer’s protocol. Protein concentration was quantified by Pierce BCA Protein Assay (Fisher Scientific) using the manufacturer’s protocol. Aliquots of the enzymes were stored at −80°C. Enzymes were thawed on ice prior to use. We did not observe any loss of activity after storage.

### Protein thermostability assay

Enzymes in cell lysates were normalized to the same concentration, 0.5 μM or 1 μM, and incubated for 1 h in a thermal cycler (MJ Research; model PTC-200) at a range of temperatures, from 60°C to 85°C, in reaction buffer (1 M or 100 mM potassium phosphate, pH 8) in PCR tubes. Following heat treatment, samples were removed, transferred to 1.5-mL microtubes, and centrifuged at 14,000 × *g* for 3 min to separate aggregated protein and cell debris. Supernatant was removed and GFP complementation was used to quantify the amount of soluble enzyme remaining by diluting 1:10 in a solution of GFP1-10 in TNG buffer (as above; see Groseclose et al.^10^ and Cabantous et al.^24^) and incubated for 4 h to overnight, shaking, at room temperature, in the wells of Corning MaxiSorp 96-well plates. Background fluorescence was subtracted from all samples. Fluorescence was measured using a Tecan M Plex Plate Reader (ex: 488 nm, em: 520 nm). All samples were performed in triplicate. Remaining protein was compared with initial concentrations to determine fraction/percentage of protein retained.

### Small-scale PET hydrolysis reactions

Reactions were performed with 0.69 or 0.345 μM enzyme and 2.9% (w/v) loading PET (0.35 or 0.7 mg enzyme/g PET for PHL7-WT) in 500 μL evaporation-proof cryo-vials (Simport Scientific; product T309-2A). Reactions were composed of PET, enzymes (diluted with lysis buffer), and appropriate potassium phosphate buffer (of varied pH and concentration). PET was either in the form of milled powder (added prior to reaction buffer and aliquoted into reactions after re-suspension) or as film in the form of 3-mm hole-punched circular coupons (Fiskars). Time points were taken at 2, 4, 6, 8, 24, 48, and 72 h, incubating at the reaction temperature. Samples were taken for absorbance measurement and HPLC analysis. For HPLC, samples were immediately diluted 50% (v/v) with methanol and then filtered using a 0.2-μm plate filter using MultiScreen HTS Filter Plates (Millipore Sigma; product MSGVN2250). Absorbance measurement was performed as above^25^ and our previous report.^10^ Samples were stored at −20°C until analysis. As necessary, samples for absorbance and HPLC analysis were diluted with ultrapure water. All reactions were performed in triplicate.

### Monomer quantification

Concentrations of monomers TPA, MHET, and BHET were quantified by HPLC using an Agilent Technologies Infinity II 1260, equipped with a G7115A diode array detector (DAD), detecting signal at 240 nm. Samples were analyzed using a protocol adapted from Knott et al.^57^ Ten microliters of sample maintained at 10°C was injected onto a Phenomenex Luna C18(2) (100 Å, 150 mm × 4.6 mm, 5 μm) at 40°C. The mobile phase consisted of (A) 20 mM phosphoric acid in ultrapure water and (B) 100% methanol. The flow rate was a constant at 1.2 mL/min for a total time of 10 min per sample. An A:B gradient program was used, as follows: 80:20 at t = 0 min; a gradient to 35:65 by t = 7.5 min; and held constant at 80:20 from t = 7.51 min to 10 min. A calibration curve, from 0.1 to 500 mg/L, was used for each analyte to determine concentrations.

### Enzyme denaturation kinetics by differential scanning calorimetry

Denaturation thermograms were acquired for each enzyme by differential scanning calorimetry (DSC) on a MicroCal PEAQ-DSC automated instrument (Malvern Panalytical). Just before DSC analysis, each sample was purified by size exclusion chromatography on a HiLoad Superdex 75 pg column (Cytiva) pre-equilibrated with 100 mM sodium phosphate pH 8. For each enzyme, thermograms were recorded on 0.8 mg/mL samples across the temperature range of 50°C–100°C at five different ramp rates (0.2, 0.4, 0.8, 1.6, and 3.2°C/min) in low feedback mode. The instrument’s control and analysis software was used to perform buffer subtraction and baseline correction. For each enzyme variant, the set of thermograms were fit globally to a variety of different kinetic models using the CalFitter v2.0 webserver,^58^ including either a single-step irreversible process (i.e., native to denatured), a single-step reversible process, or a two-step irreversible process (i.e., denaturation via an intermediate). More complex models were not considered. In each model, each enzyme state transition is described by a calorimetric enthalpy (Δ*H*_cal_), an activation energy (*E*_a_), and a reference temperature (*T∗*) at which one enzyme molecule per second transits. At a given temperature *T* (in Kelvin), the rate constant (*k*) for each transition is related to its *E*_a_ and *T*∗ by the Arrhenius relationship:

(Equation 1) $$k=\exp\left(-\frac{E_{a}}{R}\left[\frac{1}{T}-\frac{1}{T^{*}}\right]\;\right)\text{,}$$

where *R* is the universal gas constant (8.314 J/K/mol). Note that *T*∗ is dependent on *E*_a_ since:

(Equation 2) $$T^{*}=\frac{E_{a}}{R}.\ln\left(A\right)\text{,}$$

where *A* is the Arrhenius frequency factor for the transition, which we assume to be constant over the narrow temperature range investigated.

### PET hydrolysis in pH-controlled bioreactors

Enzymatic PET hydrolysis reactions at 200 mL scale were carried out in duplicate using Applikon MiniBio bioreactor systems with 250 mL glass vessels (Getinge AB, Sweden) equipped with one marine impeller. Amorphous PET film of 0.25-mm thickness (Goodfellow) was cut into approximately 10 × 10-mm squares, washed with 70% EtOH, and incubated at 40°C until completely dry. These PET film squares were added to the reactor at a given solids loading (2.9%, 5.8%, or 20% [w/v]) in 1 M sodium phosphate buffer, pH 8. The suspension was pre-equilibrated to 65°C with stirring at 400 rpm. The reaction was initiated by the addition of enzyme to 1 mg/g PET. Depolymerization reactions proceeded for 48 h with continuous pH control through the intermittent addition of 6 or 9.5 M NaOH using a peristaltic pump control module (Applikon my-Control). The “% hydrolysis” in the bioreactor experiments is inferred from the cumulative volume of base solution (6 or 9.5 M NaOH) used to maintain the pH (i.e., to neutralize the liberated acid), with 100% corresponding to the theoretical volume needed to neutralize complete hydrolysis of the PET into TPA and ethylene glycol. At the end of the reaction, any remaining substrate was recovered by filtration through a Whatman glass microfiber filter (Cytiva) using a Büchner funnel. The retained solid residue was washed with ultrapure water to remove any precipitated salts and dried at 40°C overnight prior to obtaining the residual dry weight, from which the percentage mass loss was calculated.

### Protein modeling

The online platform ColabFold^59^ that uses the AlphaFold2 (AF2) structure prediction tool^41^ to predict three-dimensional structures from amino acid sequences, was employed to predict the structure of all PHL7 variants including the native PHL7 sequence. The X-ray crystal structure of PHL7 was acquired from the protein databank (PDB code: 7NEI)^19^ and was used as a reference to determine the root mean square deviation (RMSD) of Cα backbone atoms of each AF2 predicted structures. The alignment of the predicted structures with the crystal structure was performed using PyMOL Molecular Graphics System (version 2.3.0, Schrodinger, LLC). To gain insight into mutations that could be stabilizing, we performed computational design with backbone flexibility (FastDesign protocol in ROSETTA)^43^ to probe the preference of each mutation that accumulated during directed evolution over the native amino acid. Stabilizing mutations are expected to be preferred in the FastDesign protocol for protein design. For determining the PHL7/PET interaction, a model PET substrate dubbed PET3mer consisting of three terephthalic acid and three ethylene glycol subunits was created using the Avogadro molecule editor software.^60^ The PET3mer conformers were generated using the BCL::CONF conformer ensemble generator^61^ that created 480 conformers of PET3mer. The PET3mer was docked in the space around the catalytic triad that included a sphere of 10-Å radius with a center at the gamma oxygen of catalytic residue S131. The RosettaLigand protocol utilizes all provided conformers of the ligand and performs docking followed by side chain repacking and backbone minimization at the interface^45^ Approximately 14,000 docking trajectories were run for each PHL7 variant (PHL7-Jemez, PHL7-WT and PHL7-L93F/Q95Y). Of 14,000 poses, the top 10% based on total score followed by top 5 poses based on ligand binding score were selected for visual inspection on PyMOL. The poses with the scissile bond of PET3mer in close proximity (5.6–6.3 Å) to the S131 were chosen to be the representative model for PHL7/PET3mer interaction at the stage of substrate recruitment.

### Sequence analysis

The MPI Bioinfomatics toolkit^28^ provides a platform for submitting a protein sequence and user-defined database to perform search using PSI-BLAST+ method. PHL7-WT sequence was used as an input to search over the nr70 database (a non-redundant database curated down to 70% sequence identity, updated on December 27, 2024) for homologous sequences using default parameters such as BLOSUM62 scoring matrix and e-value cutoff of 1 × 10^−3^. The output of the PSI-BLAST+ search was automatically sent to ClustalΩ^62^ to produce a multiple sequence alignment of 250 homologous sequences, which was further analyzed for sequence diversity at the 11 mutated sites of PHL7 indicated in Figure 3J.

## Resource availability

### Lead contact

Requests for further information and resources should be directed to Hau B. Nguyen (hau@lanl.gov).

### Materials availability

Requests for materials will be fulfilled by the lead contact upon reasonable request. The nucleotide sequences of the following genes have been made available in GenBank with the corresponding accession numbers: PHL7-WT (GenBank: PQ223703), PHL7-Jemez (GenBank: PQ223704), PHL7-Santa Fe (GenBank: PQ223705), PHL7-Taos (GenBank: PQ223706), PHL7-Tusas (GenBank: PQ223707), PHL7-L93F/Q95Y (GenBank: PQ223708), and LCC-ICCG (GenBank: PQ223702). The following genes of the mutants along the evolutionary trajectory of the final mutants have additionally been made available in GenBank with the corresponding accession numbers: PHL7-A1 (GenBank: PQ223709), PHL7-A2 (GenBank: PQ223710), PHL7-A3 (GenBank: PQ223711), PHL7-A4 (GenBank: PQ223712), PHL7-A5 (GenBank: PQ223713), PHL7-A6 (GenBank: PQ223714), PHL7-B1 (GenBank: PQ223715), PHL7-B2 (GenBank: PQ223716), PHL7-B3 (GenBank: PQ223717), PHL7-C1 (GenBank: PQ223718), PHL7-C2 (GenBank: PQ223719), PHL7-C3 (GenBank: PQ223720), PHL7-C4 (GenBank: PQ223721), PHL7-C5 (GenBank: PQ223722), and PHL7-C6 (GenBank: PQ223723).

### Data and code availability

Supplemental data are available in the supplemental information. Requests for additional data or code will be fulfilled by the lead contact upon reasonable request.

## Acknowledgments

This work was performed as part of the Bio-Optimized Technologies to keep Thermoplastics out of Landfills and the Environment (BOTTLE) Consortium and was supported by the Advanced Manufacturing Office and Bioenergy Technologies Office under contract DE-AC36-08GO28308 with the National Renewable Energy Laboratory, operated by Alliance for Sustainable Energy, LLC, and under contract NL0035994 with Los Alamos National Laboratory (LANL), operated by Triad National Security, LLC. T.M.G. and H.B.N. also acknowledge the LANL Directed Research and Development program for funding project #20220807PRD4. H.B.N. also thanks the LANL Test and Evaluation program for funding project #WC3N/25ENZYME and the Feynman Center for Innovation for funding project #XB3B00/19252403. A.R.P, M.C., and B.M. were supported by 10.13039/501100013589Research England through the Expanding Excellence in England (E3) scheme. A.R.P. also thanks the 10.13039/501100000268BBSRC for financial support (grants BB/X011410/1 and BB/Y007972/1). We would like to thank Bailee Nasise and Shloka Bhakta for their help with screening libraries of PHL7 and Theresa Kern and Lyman Monroe for their assistance with HPLC data collection and analysis. We acknowledge BioRender for assistance with creation of the figures. This research used resources provided by the Los Alamos National Laboratory Institutional Computing Program, which is supported by the US Department of Energy National Nuclear Security Administration under contract no. 89233218CNA000001. This work is released for publication in accordance with LANL LA-UR-24-24196 by Triad National Security, LLC operator of the Los Alamos National Laboratory under contract no. 89233218CNA000001 with the US Department of Energy.

## Author contributions

Conceptualization: H.B.N., T.D., T.M.G., and G.T.B.; data curation: T.M.G., R.K.J., A.R.P., and H.B.N.; formal analysis: T.M.G., M.C., B.M., R.K.J., A.R.P., and H.B.N.; funding acquisition: G.T.B., A.R.P., T.D., and H.B.N.; investigation: T.M.G., E.K., M.C., B.M., R.K.J., Z.K.T., L.A.L., A.R.P., and H.B.N.; methodology: T.M.G., A.R.P., and H.B.N.; project administration: R.K.J., G.T.B., A.R.P., T.D., and H.B.N.; supervision: G.T.B., A.R.P., T.D., and H.B.N.; visualization: T.M.G., R.K.J., A.R.P., and H.B.N.; writing – original draft: T.M.G., R.K.J., A.R.P., and H.B.N.; and writing – review & editing: all authors.

## Declaration of interests

The high-throughput screening platform, methods, and enzyme variants are the subject of domestic and foreign patent applications by Los Alamos National Laboratory on behalf of the Department of Energy and Triad National Security, LLC.

## Supplemental information

Document S1. Figures S1–S23, Tables S1–S5, S7–S10, and Notes S1–S3

Table S6. Nucleotide DNA sequences for enzymes along the evolutionary trajectory

Data S1. *Chem Catalysis* checklist

Document S2. Article plus supplemental information
