# Supplementary material for: Engineering PHL7 for improved poly(ethylene terephthalate) depolymerization via rational design and directed evolution
Source: Chem Catal. 2025 Aug 21;5(8):101399. doi: 10.1016/j.checat.2025.101399 (PMC12371127; doi:10.1016/j.checat.2025.101399)
Supplement: Document S1. Figures S1–S23, Tables S1–S5, S7–S10, and Notes S1–S3 [file mmc1.pdf]

Chem Catalysis, Volume *5*

Supplemental information

Engineering PHL7 for improved poly(ethylene terephthalate) depolymerization via rational design and directed evolution

Thomas M. Groseclose, Erin Kober, Matilda Clark, Benjamin Moore, Ramesh K. Jha, Zoe K. Taylor, Lexy A. Lujan, Gregg T. Beckham, Andrew R. Pickford, Taraka Dale, and Hau B. Nguyen

Note S1: In our HPLC analysis, TPA and MHET concentrations for reactions were abundant and readily measured, while BHET concentrations were generally low and are thereby not explicitly discussed. However, BHET concentrations were factored into calculations of the sum of aromatic products: the sum total concentration of TPA, MHET, and BHET.

Note S2: On high crystallinity (41.8% crystallinity[1]) PET powder, our engineered enzymes' activities were reduced by up to 11-fold, while PHL7-WT's was reduced by about 4-fold, and LCC-ICCG's by 3-fold (comparing up to 8 h) (**Figure 4b**, **Figure S9d-f**, **Figure S10b**). As a result, LCC-ICCG had the highest activity of all the enzymes we tested on high crystallinity PET, with on average, about 50% higher activity than our variants by 8- and 72 h time points. However, our engineered enzymes showed significantly improved activities over PHL7-WT on high crystallinity PET. For example, PHL7-Jemez had a 1.3-fold increase over PHL7 (**Figure S9d**). This is likely a consequence of our screening method, as high crystallinity substrates were not used in screening, and despite the high reaction temperatures approaching 70 °C, these enzymes are thought to preferentially depolymerize the amorphous regions of the polymer.[2,3] Future work, however, could adapt our screening methods to engineer against high crystallinity PET. Further, the PHL7 enzyme variants could be of use in enzyme cocktails with LCC-ICCG, as it appears that LCC-ICCG, in reactions with high crystallinity PET, accumulates MHET, whereas the engineered variants here appear readily able to convert residual MHET, with concentrations rapidly decreasing after 24 h (when we posit the more freely-accessible PET becomes limiting, and overall conversion begins to plateau) (**Figure S9c,f**). To date, no MHETase exists that can function at 70 °C. Synergistic reactions with LCC-ICCG and PHL7-Jemez could serve as viable alternatives to facilitate higher extents of total PET conversion.

Note S3: For reactions at pH 9, we observed similar results to the reactions at pH 8 (**Figure S11**, **Figure S7c-d**). The total product release between the two conditions were similar for our engineered enzymes (about 15 g/L), and PHL7-WT (about 4.5 g/L). However, we did see that LCC-ICCG had initial higher activity than at pH 8, with about 40% higher activity by 8 h at pH 9 (**Figure S11a**). Though, similar to pH 8 with amorphous coupons, we observed complete conversion of the PET by 24 h by our variants (i.e., the coupons disappeared entirely), prompting us to run our time-course reaction to 72 h, at a reduced enzyme loading (0.35 mg enzyme/g PET). Here, we saw that our engineered enzymes out-performed LCC-ICCG and PHL7-WT over time, namely, with product continuing to be released to 72 h, while LCC-ICCG's activity appeared to plateau by 24 h (**Figure S11d**). Taken together, we posit that LCC-ICCG's higher apparent activity may be due to the pH in the reaction maintaining for a longer time around its optimum, pH 8, in a reaction starting at pH 9, whereas the PHL7 variants are stable at a wider range of pH. Interestingly, LCC-ICCG has a predicted isoelectric point (pI) of 8.9, whereas PHL7 has a predicted pI of 5.3.[4]

Table S1: Nucleotide DNA sequence for expression cassette in GFP11 plasmid vector.

PHL7-WT in pET21b(+)-GFP11 Cassette<br><br>**Key:**<br>• T7 Promoter<br>• *lac* Operator<br>• RBS<br>• Restriction Sites (NdeI / BamHI)<br>• ***PHL7-WT***<br>• Linker<br>• GFP-11<br>• T7 Terminator	TAATACGACTCACTATAGGGGAATTGTGAGCGGATAACAATTCCCCTCTA<br>GAAATAATTTTGTTTAACTTTAAGAAGGAGATATACAT**ATGGCGAATCCGT**<br>**ATGAGCGTGGCCCGGACCCGACCGAGAGCAGCATTGAAGCGGTTCG**<br>**TGGCCCGTTTGCGGTTGCGCAGACCACCGTTAGCCGTCTGCAGGCGG**<br>**ATGGCTTCGGTGGTGGCACCATCTACTATCCGACCGACACCAGCCAA**<br>**GGCACCTTCGGTGCGGTGGCGATTAGCCCGGGCTTTACCGCGGTCA**<br>**GGAGAGCATTGCGTGGCTGGGTCCGCGTATTGCGAGCCAAGGTTTTG**<br>**TGGTTATCACCATTGATACCATCACCCGTCTGGATCAGCCGGACAGCC**<br>**GTGGCCGTCAGCTGCAAGCGGCGCTGGATCACCTGCGTACCAACAG**<br>**CGTGGTTCGTAACCGTATTGACCCGAACCGTATGGCGGTTATGGGTCA**<br>**CAGCATGGGCGGTGGtGGTGCGCTGAGCGCTGCGGCGAACAACACC**<br>**AGCCTGGAAGCGGCGATCCCGCTGCAGGGTTGGCACACCCGTAAGA**<br>**ACTGGAGCAGCGTGCGTACCCCGACCCTGGTGGTTGGTGCGCAACT**<br>**GGATACCATTGCGCCGGTTAGCAGCCACAGCGAGGCGTTCTACAACA**<br>**GCCTGCCGAGCGATCTGGACAAAGCGTATATGGAACTGCGTGGTGCG**<br>**AGCCACCTGGTGAGCAACACCCCGGACACCACCACcGCGAAGTACA**<br>**GCATCGCGTGGCTGAAACGTTTTGTTGACGATGACCTGCGTTATGAGC**<br>**AATTTCTGTGCCCGGCGCCGGACGACTTTGCGATTAGCGAATACCGTA**<br>**GCACCTGCCCGTTC**GGATCCGATGGAGGGTCTGGTGGCGGATCAACT<br>AGTCGTGACCACATGGTCCTTCATGAGTACGTAAATGCTGCTGGGATTA<br>CATAAGGTACCTAACTCGAGCACCACCACCACCACCACTGAGATCCGG<br>CTGCTAACAAAGCCCGAAAGGAAGCTGAGTTGGCTGCTGCCACCGCT<br>GAGCAATAACTAGCATAACCCCTTGGGGCCTCTAAACGGGTCTTGAGG<br>GGTTTTTTG

Table S2: Thermostability of enzymes across the evolutionary trajectory, expressed as percentage of protein retained after heat treatment at 75 °C for 1 and 2 hours. Percentage of protein is the ratio of final protein concentration, quantified by split-GFP complementation, to initial. Enzymes were normalized to 1 μM and reactions were in 100 mM potassium phosphate buffer, pH 8. Percentages are averages if of n = 2 reactions, with ±1 S.D. shown.

Variant	Protein Amount Retained after Heat Treatment (%)	
	1 h at 75 °C	2 h at 75 °C
PHL7-WT	33.79 ± 0.31	28.83 ± 0.92
PHL7-A1	51.85 ± 1.90	36.67 ± 0.82
PHL7-A2	61.63 ± 2.73	43.14 ± 1.54
PHL7-A3	67.73 ± 2.76	48.37 ± 1.90
PHL7-A4	36.14 ± 1.73	28.55 ± 1.17
PHL7-A5	36.26 ± 1.63	32.24 ± 0.80
PHL7-A6	40.13 ± 1.05	37.43 ± 1.58
PHL7-B1	31.78 ± 1.09	27.75 ± 1.26
PHL7-B2	49.28 ± 4.28	48.94 ± 2.23
PHL7-B3	52.68 ± 0.45	37.08 ± 1.89
PHL7-C1	34.84 ± 1.07	34.00 ± 0.52
PHL7-C2	28.96 ± 0.72	26.45 ± 0.54

PHL7-C3	49.89	±	4.98	48.29	±	1.60
PHL7-C4	28.52	±	0.68	26.07	±	0.53
PHL7-C5	26.87	±	1.03	22.07	±	0.43
PHL7-C6	34.04	±	0.25	29.16	±	0.70
PHL7-Jemez	32.55	±	0.49	30.85	±	0.30
PHL7-Santa Fe	33.42	±	0.45	28.11	±	0.59
PHL7-Taos	37.73	±	1.32	31.65	±	0.73
PHL7-Tusas	73.48	±	2.98	73.96	±	1.19

Table S3: Nucleotide DNA sequences for enzymes. Highlighted nucleotides for PHL7 variants denote those that differ from PHL7-WT.

Name	DNA Sequence
PHL7-WT[5]	ATGGCGAATCCGTATGAGCGTGGCCCGGACCCGACCGAGAGCAGCATT<br>GAAGCGGTTCGTGGCCCGTTTGCGGTTGCGCAGACCACCGTTAGCCGT<br>CTGCAGGCGGATGGCTTCGGTGGTGGCACCATCTACTATCCGACCGAC<br>ACCAGCCAAGGCACCTTCGGTGCGGTGGCGATTAGCCCGGGCTTTACC<br>GCGGGTCAGGAGAGCATTGCGTGGCTGGGTCCGCGTATTGCGAGCCA<br>AGGTTTTGTGGTTATCACCATTGATACCATCACCCGTCTGGATCAGCCG<br>GACAGCCGTGGCCGTCAGCTGCAAGCGGCGCTGGATCACCTGCGTAC<br>CAACAGCGTGGTTCGTAACCGTATTGACCCGAACCGTATGGCGGTTATG<br>GGTCACAGCATGGGCGGTGGtGGTGCGCTGAGCGCTGCGGCGAACAA<br>CACCAGCCTGGAAGCGGCGATCCCGCTGCAGGGTTGGCACACCCGTA<br>AGAACTGGAGCAGCGTGCGTACCCCGACCCTGGTGGTTGGTGCGCAA<br>CTGGATACCATTGCGCCGGTTAGCAGCCACAGCGAGGCGTTCTACAAC<br>AGCCTGCCGAGCGATCTGGACAAAGCGTATATGGAACTGCGTGGTGCG<br>AGCCACCTGGTGAGCAACACCCCGGACACCACCACcGCGAAGTACAGC<br>ATCGCGTGGCTGAAACGTTTTGTTGACGATGACCTGCGTTATGAGCAAT<br>TTCTGTGCCCGGCGCCGGACGACTTTGCGATTAGCGAATACCGTAGCA<br>CCTGCCCGTTC
PHL7-Jemez	ATGGCGAATCCGTATGAGCGTGGCCCGGACCCGACCGAGAGCAGCATT<br>GAAGCGGTTCGTGGCCCGTTTGCGGTTGCGCAGACCACCGTTAGCCGT<br>CTGCAGGTGGATGGCTTCGGTGGTGGCACCATCTACTATCCGACCGAC<br>ACCAGCCAAGGCACCTTCGGTGCGGTGGCGATTAGCCCGGGCTTTACC<br>GCGGGTCAGGAGAGCATTGCGTGGCTGGGTCCGCGTATTGCGAGCCA<br>AGGTTTTGTGGTTATCACCATTGATACCATCACCCGTCTGGATTATCCGG<br>ACAGCCGTGGCCGTCAGCTGCAAGCGGCGCTGGATCACCTGCGTATCA<br>ACAGCGTGGTTCGTAACCGTATTGACCCGAACCGTATGGCGGTTATGGG<br>TCACAGCATGGGCGGTGGTGGTGCGCTGAGCGCTGCGGCGAACAACA<br>CCAGCCTGGAAGCGGCGATCCCGCTGCAGGGTTGGCACACCCGTAAG<br>AACTGGAGCAGCGTGCGTACCCCGACCCTGGTGGTTGGTGCGGAACT<br>GGATACCATTGCGCCGGTTAGCAGCAACAGCGAGGCGTTCTACAACAG<br>CCTGCCGAGCGATCTGGACAAAGCGTATATGGAACTGCGTGGTGCGAG<br>CCACCTGGTGAGCAACACCCCGGACACCACCACCGCGAAGTACAGCAT<br>CGCGTGGCTGAAACGTTTTGTTGACGATGACCTGCGTTATGAGCAATTT<br>CTGTGCCCGGCGCCGGACGACTTTGCGATTAGCGAATACCGTAGCACC<br>TGCCCGTTC
PHL7-Santa Fe	ATGGCGAATCCGTATGAGCGTGGCCCGGACCCGACCGAGAGCAGCATT<br>GAAGCGGTTCGTGGCCCGTTTGCGGTTGCGCAGACCACCGTTAGCCGT<br>CTGCAGGCGGATGGCTTCGGTGGTGGCACCATCTACTATCCGACCGAC

	ACCAGCCAAGGCACCTTCGGTGCGGTGGCGATTAGCCCGGGCTTTACC<br>GCGGGTCAGGAGAGCATTGCGTGGCTGGGTCCGCGTATTGCGAGCCAT<br>GGTTTTGTGGTTATCACCATTGATACCATCACCCGTCTGGATTATCCGGA<br>CAGCCGTGGCCGTCAGCTGCAAGCGGCGCTGGATCACCTGCGTACCA<br>ACAGCGTGGTTCGTAACCGTATTGACCCGAACCGTATGGCGGTTATGGG<br>TCACAGCATGGGCGGTGGTGGTGCGCTGAGCGCTGCGGCGAATAACA<br>CCAGCCTGGAAGCGGCGATCCCGCTGCAGGGTTGGCACACCCGTAAG<br>AACTGGAGCAGCGTGCGTACCCCGACTCTGGTGGTTGGTGCGGAACT<br>GGATACCATTGCGCCGGTTAGCAGCAACAGCGAGGCGTTCTACAACAG<br>CCTGCCGAGCGATCTGGACAAAGCGTATATGGAACTGAAAGGTGCGAG<br>CCACCTGGTGAGCAACACCCCGGACACCACCACCGCGAAGTACAGCAT<br>CGCGTGGCTGAAACGTTTTGTTGACGATGACCTGCGTTATGAGCAATTT<br>CTGTGCCCGGCGCCGGACGACTTTGCGATTAGCGAATACCGTAGCACC<br>TGCCCGTTC
PHL7-Taos	ATGGCGAATCCGTATGAGCGTGGCCCGGACCCGACCGAGAGCAGCATT<br>GAAGCGGTTCGTGGCCCGTTTGCGGTTGCGCAGACCACCGTTAGCCGT<br>CTGCAGGCGGATGGCTTCGGTGGTGGCACCATCTACTATCCGACCGAC<br>ACCAGCCAAGGCACCTTCGGTGCGGTGGCGATTAGCCCGGGCTTTACC<br>GCGGGTCAGGAGAGCATTGCGTGGCTGGGTCCGCGTATTGCGAGCCA<br>AGGTTTTGTGGTTATCACCATTGATACCATCACCCGTCTGGATCAGCCG<br>GACAGCCGTGGCCGTCAGCTGCAAGCGGCGCTGGATCACCTGCGTGC<br>CAACAGCGTGGTTCGTAACCGTATTGACCCGAACCGTATGGCGGTTATG<br>GGTCACAGCATGGGCGGTGGCGGTGCGCTGAGCGCTGCGGCGAACAA<br>CACCAGCCTGGAAGCGGCGATCCCGCTGCAGGGTTGGCACACCCGTA<br>AGAACTGGAGCAGCGTGCGTACCCCGACCCTGGTGGTTGGTGCGGAA<br>CTGGATACCATTGCGCCGGTTAGCAGCAACAGCGAGGCGTTCTACAAC<br>AGCCTGCCGAGCGATCTGGACAAAGCGTATATGGAACTGCGTGGTGCG<br>AGCCACCTGGTGAGCAACACCCCGGACACCACCACCGCGAAGTACAG<br>CATCGCGTGGCTGAAACGTTTTGTTGACGATGACCTGCGTTATGAGCAA<br>TTTCTGTGCCCGGCGCCGGACGACTTTGCGATTAGCGAATACCGTAGCA<br>CCTGCCCGTTC
PHL7-Tusas	ATGGCGAATCCGTATGAGCGTGGCCCGGACCCGACCGAGAGCAGCATT<br>GAAGCGGTTCGTGGCCCGTTTGCGGTTGCGCAGACCACCGTTAGCCCT<br>CTGCAGGCGGATGGCTTCGGTGGTGGCACCATCTACTATCCGACCGAC<br>ACCAGCCAAGGCACCTTCGGTGCGGTGGCGATTAGCCCGGGCTTTAGC<br>GCGGGTCAGGAGAGCATTGCGTGGCTGGGTCCGCGTATTGCGAGCCA<br>AGGTTTTGTGGTTATCACCATTGATACCATCACCCGTCTGGATCAGCCG<br>GACAGCCGTGGCCGTCAGCTGCAAGCGGCGCTGGATCACCTGCATAC<br>CAACAGCGTGGTTCGCAACCGGATTGACCCGAACCGTATGGCGGTTAT<br>GGGTCACAGCATGGGCGGTGGCGGTGCGCTGAGCGCTGCGGCGAAC<br>AACACCAGCCTGGAAGCGGCGATCCCGCTGCAGGGTTGGCACACCCG<br>TAAGAACTGGAGCAGCGTGCGTACCCCGACCCTGGTGGTTGGTGCGG<br>AACTGGATACCATTGCGCCGGTTAGCAGCAACAGCGAGGCGTTCTACAA<br>CAGCCTGCCGAGCGATCTGGACAAAGCGTATATGGAACTGCGTGGTGC<br>GAGCCACCTGGTGAGCAACACCCCGGACACCACCACCGCGAAGTACA<br>GCATCGCGTGGCTGAAACGTTTTGTTGACGATGACCTGCGTTATGAGCA<br>ATTTCTGTGCCCGGCGCCGGACGACTTTGCGATTAGCGAATACCGTAGC<br>ACCTGCCCGTTC
PHL7-L93F/Q95Y[6]	ATGGCGAATCCGTATGAGCGTGGCCCGGACCCGACCGAGAGCAGCATT<br>GAAGCGGTTCGTGGCCCGTTTGCGGTTGCGCAGACCACCGTTAGCCGT<br>CTGCAGGCGGATGGCTTCGGTGGTGGCACCATCTACTATCCGACCGAC<br>ACCAGCCAAGGCACCTTCGGTGCGGTGGCGATTAGCCCGGGCTTTACC<br>GCGGGTCAGGAGAGCATTGCGTGGCTGGGTCCGCGTATTGCGAGCCA<br>AGGTTTTGTGGTTATCACCATTGATACCATCACCCGTTTTGATTATCCGG

	ACAGCCGTGGCCGTCAGCTGCAAGCGGCGCTGGATCACCTGCGTACC<br>AACAGCGTGGTTCGTAACCGTATTGACCCGAACCGTATGGCGGTTATGG<br>GTCACAGCATGGGCGGTGGtGGTGCGCTGAGCGCTGCGGCGAACAAC<br>ACCAGCCTGGAAGCGGCGATCCCGCTGCAGGGTTGGCACACCCGTAA<br>GAACTGGAGCAGCGTGCGTACCCCGACCCTGGTGGTTGGTGCGCAAC<br>TGGATACCATTGCGCCGGTTAGCAGCCACAGCGAGGCGTTCTACAACA<br>GCCTGCCGAGCGATCTGGACAAAGCGTATATGGAACTGCGTGGTGCGA<br>GCCACCTGGTGAGCAACACCCCGGACACCACCACcGCGAAGTACAGCA<br>TCGCGTGGCTGAAACGTTTTGTTGACGATGACCTGCGTTATGAGCAATT<br>TCTGTGCCCGGCGCCGGACGACTTTGCGATTAGCGAATACCGTAGCAC<br>CTGCCCGTTC
LCC-ICCG[7]	ATGTCTAACCCGTACCAGCGCGGACCGAACCCGACCCGTTCTGCGTTA<br>ACCGCTGATGGTCCGTTTTCCGTGGCTACCTACACCGTTTCTCGTCTGT<br>CCGTTTCCGGTTTTGGTGGTGGTGTTATCTACTATCCGACTGGTACCTC<br>TCTGACCTTCGGCGGTATCGCGATGTCCCCGGGTTACACCGCTGATGC<br>TTCCTCTCTGGCGTGGCTGGGTCGTCGCCTGGCGAGCCACGGTTTTG<br>TTGTTCTGGTTATCAACACGAACTCTCGTTTCGACGGCCCGGACTCCC<br>GTGCCTCGCAACTGTCTGCTGCGCTGAACTACCTGCGTACGTCGTCAC<br>CTTCAGCGGTCCGTGCACGCCTGGATGCCAATCGTCTGGCTGTGGCG<br>GGTCACAGCATGGGCGGTGGCGGTACCCTGCGTATTGCTGAACAGAAC<br>CCGTCCCTGAAAGCTGCAGTGCCACTGACTCCGTGGCATACCGACAAA<br>ACGTTCAACACCAGTGTTCCGGTACTGATCGTAGGCGCAGAAGCGGAC<br>ACCGTAGCACCGGTTTCCCAGCACGCAATCCCGTTCTACCAGAACCTG<br>CCGAGCACCACTCCAAAAGTATACGTTGAACTGTGCAACGCCTCGCAC<br>ATTGCTCCGAACTCGAACAACGCTGCGATTAGCGTGTACACCATCTCCT<br>GGATGAAACTGTGGGTTGATAACGATACCCGTTATCGCCAATTCCTGTG<br>TAACGTGAACGATCCGGCTCTCTGCGATTTTCGTACCAACAACCGTCAT<br>TGCCAA

Table S4: Protein sequences for enzymes.

Name	Protein Sequence
PHL7-WT[5]	M^{1}ANPYERGPDPTESSIEAVRGPFAVAQTTVSRLQADGFGGGTIYYPTDTS<br>QGTFGAVAISPGFTAGQESIAWLGPRIASQGFVVITIDTITRLDQPDSRGRQL<br>QAALDHLRTNSVVRNRIDPNRMAVMGHSMGGGGALSAAANNTSLEAAIPL<br>QGWHTRKNWSSVRTPTLVVGAQLDTIAPVSSHSEAFYNSLPSDLDKAYME<br>LRGASHLVSNTPDTTTAKYSIAWLKRFVDDDLRYEQFLCPAPDDFAISEYRS<br>TCPF
PHL7-Jemez	M^{1}ANPYERGPDPTESSIEAVRGPFAVAQTTVSRLQVDGFGGGTIYYPTDTS<br>QGTFGAVAISPGFTAGQESIAWLGPRIASQGFVVITIDTITRLDYPDSRGRQL<br>QAALDHLRINSVVRNRIDPNRMAVMGHSMGGGGALSAAANNTSLEAAIPL<br>QGWHTRKNWSSVRTPTLVVGAELDTIAPVSSNSEAFYNSLPSDLDKAYME<br>LRGASHLVSNTPDTTTAKYSIAWLKRFVDDDLRYEQFLCPAPDDFAISEYRS<br>TCPF
PHL7-Santa Fe	M^{1}ANPYERGPDPTESSIEAVRGPFAVAQTTVSRLQADGFGGGTIYYPTDTS<br>QGTFGAVAISPGFTAGQESIAWLGPRIASHGFVVITIDTITRLDYPDSRGRQL<br>QAALDHLRTNSVVRNRIDPNRMAVMGHSMGGGGALSAAANNTSLEAAIPL<br>QGWHTRKNWSSVRTPTLVVGAELDTIAPVSSNSEAFYNSLPSDLDKAYME<br>LKGASHLVSNTPDTTTAKYSIAWLKRFVDDDLRYEQFLCPAPDDFAISEYRS<br>TCPF
PHL7-Taos	M^{1}ANPYERGPDPTESSIEAVRGPFAVAQTTVSRLQADGFGGGTIYYPTDTS<br>QGTFGAVAISPGFTAGQESIAWLGPRIASQGFVVITIDTITRLDQPDSRGRQL<br>QAALDHLRANSVVRNRIDPNRMAVMGHSMGGGGALSAAANNTSLEAAIPL<br>QGWHTRKNWSSVRTPTLVVGAELDTIAPVSSNSEAFYNSLPSDLDKAYME

	LRGASHLVSNTPDTTTAKYSIAWLKRFVDDDLRYEQFLCPAPDDFAISEYRS<br>TCPF
PHL7-Tusas	M^{1}ANPYERGPDPTESSIEAVRGPFAVAQTTVSPLQADGFGGGTIYYPTDTS<br>QGTFGAVAISPGFSAGQESIAWLGPRIASQGFVVITIDTITRLDQPDSRGRQ<br>LQAALDHLHTNSVVRNRIDPNRMAVMGHSMGGGGALSAAANNTSLEAAIP<br>LQGWHTRKNWSSVRTPTLVVGAELDTIAPVSSNSEAFYNSLPSDLDKAYM<br>ELRGASHLVSNTPDTTTAKYSIAWLKRFVDDDLRYEQFLCPAPDDFAISEYR<br>STCPF
PHL7-L93F/Q95Y[6]	M^{1}ANPYERGPDPTESSIEAVRGPFAVAQTTVSRLQADGFGGGTIYYPTDTS<br>QGTFGAVAISPGFTAGQESIAWLGPRIASQGFVVITIDTITRFDYPDSRGRQL<br>QAALDHLRTNSVVRNRIDPNRMAVMGHSMGGGGALSAAANNTSLEAAIPL<br>QGWHTRKNWSSVRTPTLVVGAQLDTIAPVSSHSEAFYNSLPSDLDKAYME<br>LRGASHLVSNTPDTTTAKYSIAWLKRFVDDDLRYEQFLCPAPDDFAISEYRS<br>TCPF
LCC-ICCG[7]	M^{35}SNPYQRGPNPTRSALTADGPFSVATYTVSRLSVSGFGGGVIYYPTGTS<br>LTFGGIAMSPGYTADASSLAWLGRRLASHGFVVLVINTNSRFDGPDSRAS<br>QLSAALNYLRTSSPSAVRARLDANRLAVAGHSMGGGGTLRIAEQNPSLKA<br>AVPLTPWHTDKTFNTSVPVLIVGAEADTVAPVSQHAIPFYQNLPSTTPKVY<br>VELCNASHIAPNSNNAAISVYTISWMKLWVDNDTRYRQFLCNVNDPALCD<br>FRTNNRHCQ

Table S5: Expression yield of purified enzymes, per liter of expression culture, quantified by BCA assay and corrected for purity by SDS PAGE analysis.

Variant Name	Enzyme Expression Yield [*per L culture*] (mg)
PHL7-WT	9.50
PHL7-L93F/Q95Y	22.47
LCC-ICCG	31.64
PHL7-Jemez	31.14
PHL7-Santa Fe	31.56
PHL7-Taos	31.08
PHL7-Tusas	23.46

Table S7: Protein sequences for enzymes along the evolutionary trajectory.

Name	DNA Sequence
PHL7-A1	M^{1}ANPYERGPDPTESSIEAVRGPFAVAQTTVSRLQADGFGGGTIYYPTDTS<br>QGTFGAVAISPGFTAGQESIAWLGPRIASQGFVVITIDTITRLDYPDSRGRQL<br>QAALDHLRTNSVVRNRIDPNRMAVMGHSMGGGGALSAAANNTSLEAAIPL<br>QGWHTRKNWSSVRTPTLVVGAQLDTIAPVSSHSEAFYNSLPSDLDKAYME<br>LRGASHLVSNTPDTTTAKYSIAWLKRFVDDDLRYEQFLCPAPDDFAISEYRS<br>TCPF
PHL7-A2	M^{1}ANPYERGPDPTESSIEAVRGPFAVAQTTVSRLQADGFGGGTIYYPTDTS<br>QGTFGAVAISPGFTAGQESIAWLGPRIASQGFVVITIDTITRLDSPDSRGRQL<br>QAALDHLRTNSVVRNRIDPNRMAVMGHSMGGGGALSAAANNTSLEAAIPL<br>QGWHTRKNWSSVRTPTLVVGAQLDTIAPVSSHSEAFYNSLPSDLDKAYME<br>LRGASHLVSNTPDTTTAKYSIAWLKRFVDDDLRYEQFLCPAPDDFAISEYRS<br>TCPF
PHL7-A3	M^{1}ANPYERGPDPTESSIEAVRGPFAVAQTTVSRLQADGFGGGTIYYPTDTS<br>QGTFGAVAISPGFTAGQESIAWLGPRIASQGFVVITIDTITRLDQPDSRGRQL<br>QAALDHLRTNSVVRNRIDPNRMAVMGHSMGGGGALSAAANNTSLEAAIPL<br>QGWHTRKNWSSVRTPTLVVGAQLDTIAPVSSHSEAFYNSLPSDLDKAYME

	LRGASHLVSITPDTTTAKYSIAWLKRFVDDDLRYEQFLCPAPDDFAISEYRS<br>TCPF
PHL7-A4	M[1]ANPYERGPDPTESSIEAVRGPFAVAQTTVSRLQADGFGGGTIYYPTDTS<br>QGTFGAVAISPGFTAGQESIAWLGPRIASQGFVVITIDTITLLDQPDSRGRQL<br>QAALDHLRTNSVVRNRIDPNRMAVMGHSMGGGGALSAAANNTSLEAAIPL<br>QGWHTRKNWSSVRTPTLVVGAQLDTIAPVSSHSEAFYNSLPSDLDKAYME<br>LRGASHLVSNTPDTTTAKYSIAWLKRFVDDDLRYEQFLCPAPDDFAISEYRS<br>TCPF
PHL7-A5	M[1]ANPYERGPDPTVSSIEAVRGPFAVAQTTVSRLQADGFGGGTIYYPTDTS<br>QGTFGAVAISPGFTAGQESIAWLGPRIASQGFVVITIDTITRLDQPDSRGRQL<br>QAALDHLRTNSVVRNRIDPNRMAVMGHSMGGGGALSAAANNTSLEAAIPL<br>QGWHTRKNWSSVRTPTLVVGAQLDTIAPVSSHSEAFYNSLPSDLDKAYME<br>LRGASHLVSNTPDTTTAKYSIAWLKRFVDDDLRYEQFLCPAPDDFAISEYRS<br>TCPF
PHL7-A6	M[1]ANPYERGPDPTESSIEAVRGPFAVAQTPVSRLQADGFGGGTIYYPTDTS<br>QGTFGAVAISPGFTAGQESIAWLGPRIASQGFVVITIDTITRLDQPDSRGRQL<br>QAALDHLRTNSVVRNRIDPNRMAVMGHSMGGGGALSAAANNTSLEAAIPL<br>QGWHTRKNWSSVRTPTLVVGAQLDTIAPVSSHSEAFYNSLPSDLDKAYME<br>LRGASHLVSNTPDTTTAKYSIAWLKRFVDDDLRYEQFLCPAPDDFAISEYRS<br>TCPF
PHL7-B1	M[1]ANPYERGPDPTESSIEAVRGPFAVAQTTVSRLQADGFGGGTIYYPTDTS<br>QGTFGAVAISPGFTAGQESIAWLGPRIASQGFVVITIDTITRLDYPDSRGRQL<br>QAALDHLRTNSVVRNRIDPNRMAVMGHSMGGGGALSAAANNTSLEAAIPL<br>QGWHTRKNWSSVRTPTLVVGAQLDTIAPVSSNSEAFYNSLPSDLDKAYME<br>LRGASHLVSNTPDTTTAKYSIAWLKRFVDDDLRYEQFLCPAPDDFAISEYRS<br>TCPF
PHL7-B2	M[1]ANPYERGPDPTESSIEAVRGPFAVAQTTVSRLQADGFGGGTIYYPTDTS<br>QGTFGAVAISPGFTAGQESIAWLGPRIASQGFVVITIDTITLLDQPDSRGRQL<br>QAALDHLHTNSVVRNRIDPNRMAVMGHSMGGGGALSAAANNTSLEAAIPL<br>QGWHTRKNWSSVRTPTLVVGAQLDTVAPVSSHSEAFYNSLPSDLDKAYM<br>ELRGASHLVSNTPDTTTAKYSIAWLKRFVDDDLRYEQFLCPAPDDFAISEYR<br>STCPF
PHL7-B3	M[1]ANPYERGPDPTESSIEAVRGPFAVAQTTVSRLQADGFGGGTIYYPTDTS<br>QGTFGAVAISPGFSAGQESIAWLGPRIASQGFVVITIDTITRLDYPDSRGRQL<br>QAALDHLRTNSVVRNRIDPNRMAVMGHSMGGGGALSAAANNTSLEAAIPL<br>QGWHTRKNWSSVRTPTLVVGAQLDTIAPVSSHSEAFYNSLPSDLDKAYME<br>LRGASHLVSNTPDTTTAKYSIAWLKRFVDDDLRYEQFLCPAPDDFAISEYRS<br>TCPF
PHL7-C1	M[1]ANPYERGPDPTESSIEAVRGPFAVAQTTVSPLQADGFGGGTIYYPTDTS<br>QGTFGAVAISPGFTAGQESIAWLGPRIASQGFVVITIDTITRLDQPDSRGRQL<br>QAALDHLRANSVVRNRIDPNRMAVMGHSMGGGGALSAAANNTSLEAAIPL<br>QGWHTRKNWSSVRTPTLVVGAQLDTIAPVSSNSEAFYNSLPSDLDKAYME<br>LKGASHLVSNTPDTTTAKYSIAWLKRFVDDDLRYEQFLCPAPDDFAISEYRS<br>TCPF
PHL7-C2	M[1]ANTYERGPDPTESSIEAVRGPFAVAQTTVSRLQADGFGGGTIYYPTDTS<br>QGTFGAVAISPGFTAGQESIAWLGPRIASQGFVVITIDTITRLDYPDSRGRQL<br>QAALDHLRTNSVVRNRIDPNRMAVMGHSMGGGGALSAAANNTSLEAAIPL<br>QGWHTRKNWSSVRTPTLVVGAQLDTIAPVSSNSEAFYNSLPSDLDKAYME<br>LRGASHLVSNTPDTTTAKYSIAWLKRFVDDDLRYEQFLCPAPDDFAISEYRS<br>TCPF
PHL7-C3	M[1]ANPYERGPDPTESSIEAVRGPFAVAQTTVSRLQADGFGGGTIYYPTDTS<br>QGTFGAVAISPGFTAGQESIAWLGPRIASQGFVVITIDTITRLDQPDSRGRQL<br>QAALDHLHTNSVVRNRIDPNRMAVMGHSMGGGGALSAAANNTSLEAAIPL<br>QGWHTRKNWSSVRTPTLVVGAELDTIAPVSSNSEAFYNSLPSDLDKAYME

	LKGASHLVSNTPDTTTAKYSIAWLKRFVDDDLRYEQFLCPAPDDFAISEYRS TCPF
PHL7-C4	M^{1}ANPYERGPDPTESSIEAVRGPFAVAQTTVSRLQVDGFGGGTIYYPTDTS QGTFGAVAISPGFTAGQESIAWLGPRIASQGFVVITIDTITRLDQPDSRGRQL QAALDHLRTNSVVRNRIDPNRMAVMGHSMGGGGALSAAANNTSLEAAIPL QGWHTRKNWSSVRTPTLVVGAELDTIAPVSSNSEAFYNSLPSDLDKAYME LRGASHLVSITPDTTTAKYSIAWLKRFVDDDLRYEQFLCPAPDDFAISEYRS TCPF
PHL7-C5	M^{1}ANPYERGPDPTESSIEAVRGPFAVAQTTVSPLQADGFGGGTIYYPTDTS QGTFGAVAISPGFTAGQESIAWLGPRIASQGFVVITIDTITRLDQPDSRGRQL QAALDHLRTNSVVRNRIDPNRMAVMGHSMGGGGALSAAANNTSLEAAIPL QGWHTRKNWSSVRTPTLVVGAQLDTIAPVSSNSEAFYNSLPSDLDKAYME LRGASHLVSITPDTTTAKYSIAWLKRFVDDDLRYEQFLCPAPDDFAISEYRS TCPF
PHL7-C6	M^{1}ANPYERGPDPTESSIEAVRGPFAVAQTTVSRLQADGFGGGTIYYPTDTS QGTFGAVAISPGFSAGQESIAWLGPRIASQGFVVITIDTITRLDYPDSRGRQL QAALDHLRTNSVVRNRIDPNRMAVMGHSMGGGGALSAAANNTSLEAAIPL QGWHTRKNWSSVRTPTLVVGAQLDTIAPVSSNSEAFYNSLPSDLDKAYME LRGASHLVSITPDTTTAKYSIAWLKRFVDDDLRYEQFLCPAPDDFAISEYRS TCPF

Table S8: Residue level score contribution of residues at position 35 and neighboring position 38 in PHL7-WT, PHL7-Jemez, and PHL7-Jemez-V35A.

**Variant Name**	**Residue Identity (35/38)**	**Per Residue Score (REU)**	
		Residue 35	***Residue 38***
PHL7-WT	A/F	-2.92	-8.54
PHL7-Jemez	V/F	-0.28	-5.83
PHL7-Jemez-V35A	A/F	-2.85	-7.47

Table S9: Cartesian-ddG scores calculated for each of the mutations present in PHL7 variants.

**Variant Name**	**Wild-type amino acid / corresponding mutation**										
	R32	A35	T64	Q80	L93	Q95	R111	T112	Q175	H185	R205
PHL7-Jemez		V				Y		I	E	N	
PHL7-Santa Fe				H		Y			E	N	K
PHL7-Taos								A	E	N	
PHL7-Tusas	P		S				H		E	N	
PHL7-L93F/Q95Y					F	Y					

**Variant Name**	**Wild-type amino acid / mutation**										
	R32 P	A35 V	T64 S	Q80 H	L93F	Q95 Y	R111 H	T112I/ A	Q175 E	H185 N	R205 K
PHL7-Jemez		6.031				0.206		0.017	0.35	3.488	

PHL7-Santa Fe				2.173		-0.207			-0.038	3.098	1.119
PHL7-Taos								1.492	0.344	3.513	
PHL7-Tusas	0.886		0.669				-0.286		0.11	4.003	
PHL7-L93F/Q95Y					-0.117	0.162					

Table S10: Energetic parameters obtained by numerical analysis of the multiple scan rate differential scanning calorimetry (DSC) thermograms using a two-step, irreversible denaturation model.

**Parameter‡**	**Variant Name**	
	**PHL7-WT**	**PHL7-Jemez**
$\Delta H_{cal,1}$ (kJ/mol)	829.71 ± 5.42	794.31 ± 2.39
$\Delta E_{a,1}$ (kJ/mol)	355.84 ± 0.87	359.47 ± 0.51
T^*_1 (°C)	93.15 ± 0.04	91.76 ± 0.03
$\Delta H_{cal,2}$ (kJ/mol)	209.23 ± 5.32	92.26 ± 2.20
$\Delta E_{a,2}$ (kJ/mol)	641.64 ± 6.82	726.24 ± 9.92
T^*_2 (°C)	85.65 ± 0.10	84.21 ± 0.12

‡Parameters values (mean ± S.D.) were derived from the multiple thermograms (depicted in **Figure S21a,e**) using the program CalFitter v2.0.[8] The subscript 1 or 2 indicates either native-to-intermediate transition or the intermediate-to-denatured transition, respectively.

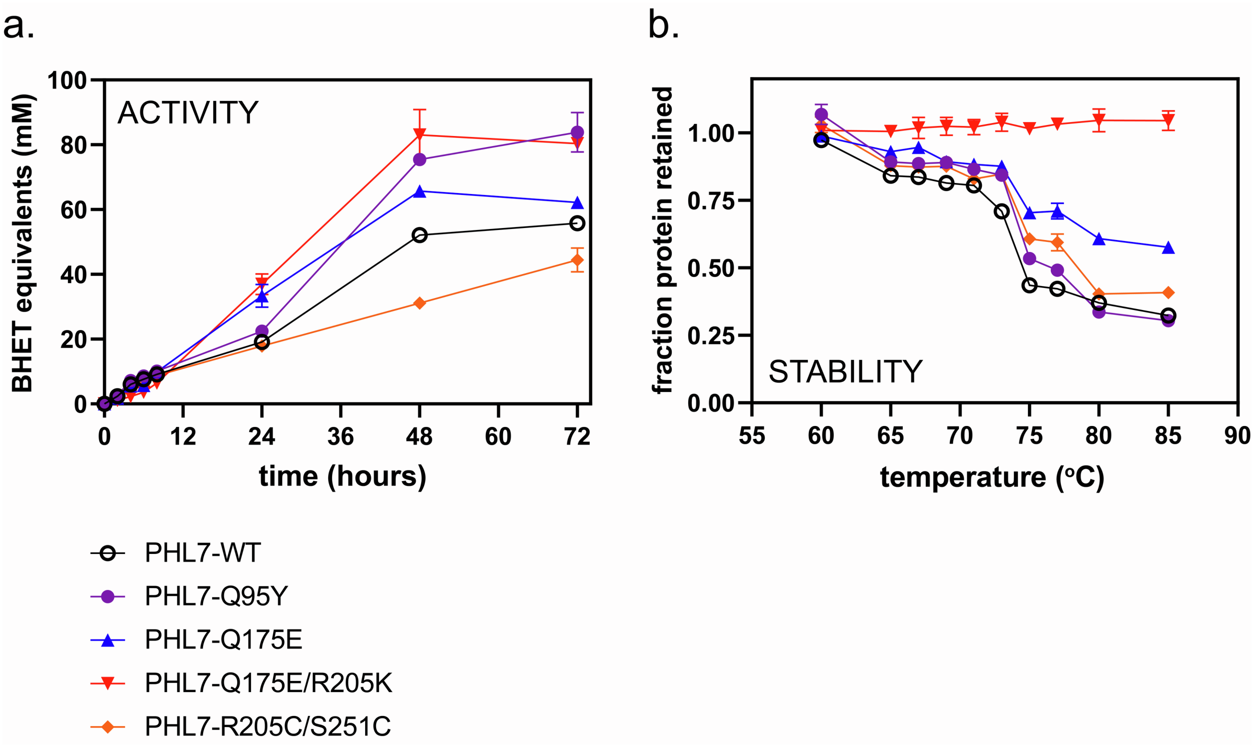

Figure S1: Activity and thermostability of rationally-designed PHL7 variants. Data is shown for PHL7-WT (white circles), active site variant PHL7-Q95Y (purple circles), salt-bridge variants PHL7-Q175E (blue triangles) and PHL7-Q175E/R205K (red inverted triangles), and disulfide bond variant PHL7-R205C/S251C (orange diamonds). **a.** Activity of the enzymes, measured by UV absorbance, expressed as equivalents of BHET, in reactions of cell lysates (containing enzymes) with PET. Reactions included 0.5 μM enzyme, 2.9% (w/v) PET coupons, 1 M potassium phosphate buffer, pH 8, and incubated at 70 °C for 72 h. **b.** Thermostability of the variants, measured by quantifying enzyme retained in solution pre- and post-heat treatment for 1 hour at varied temperatures. Fraction of protein retained is the ratio of final protein concentration, quantified by split-GFP complementation, to initial, 0.5 μM. Points display the average of n = 3 reactions, while error bars display ±1 S.D.

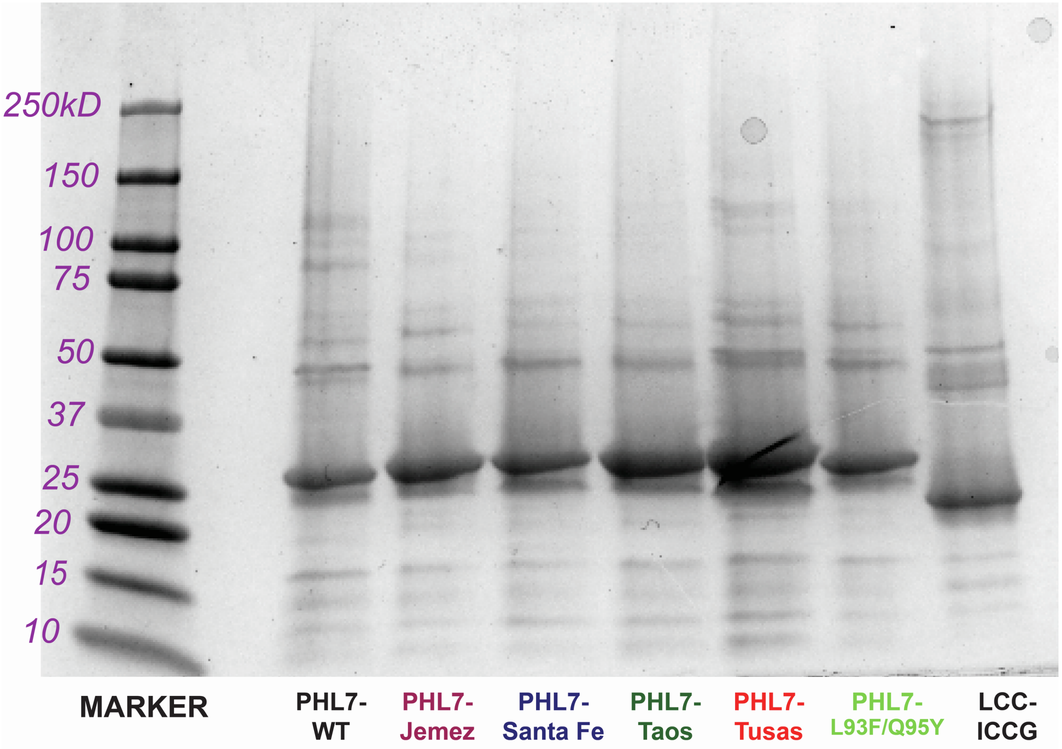

Figure S2: SDS PAGE gel of His6-tag purified proteins. Samples of His6-tag purified PHL7-WT (lane 3), PHL7-Jemez (lane 4), PHL7-Santa Fe (lane 5), PHL7-Taos (lane 6), PHL7-Tusas (lane 7), PHL7-L93F/Q95Y (lane 8), and LCC-ICCG (lane 9) proteins were run on SDS PAGE gels alongside marker (lane 1 – molecular weights denoted). Samples were boiled in Laemmli buffer for 1 hour at 100 °C prior to loading on the gel.

PHL7-WT	R32	A35	T64	Q80	L93	Q95	R111	T112	Q175	H185	R205
Mutations	P	V	S	H	F	Y	H	I/A	E	N	K
Frequency	0.004	0.468	0.004	0.232	0.176	0.012	0	0/0.052	0.584	0.008	0.036

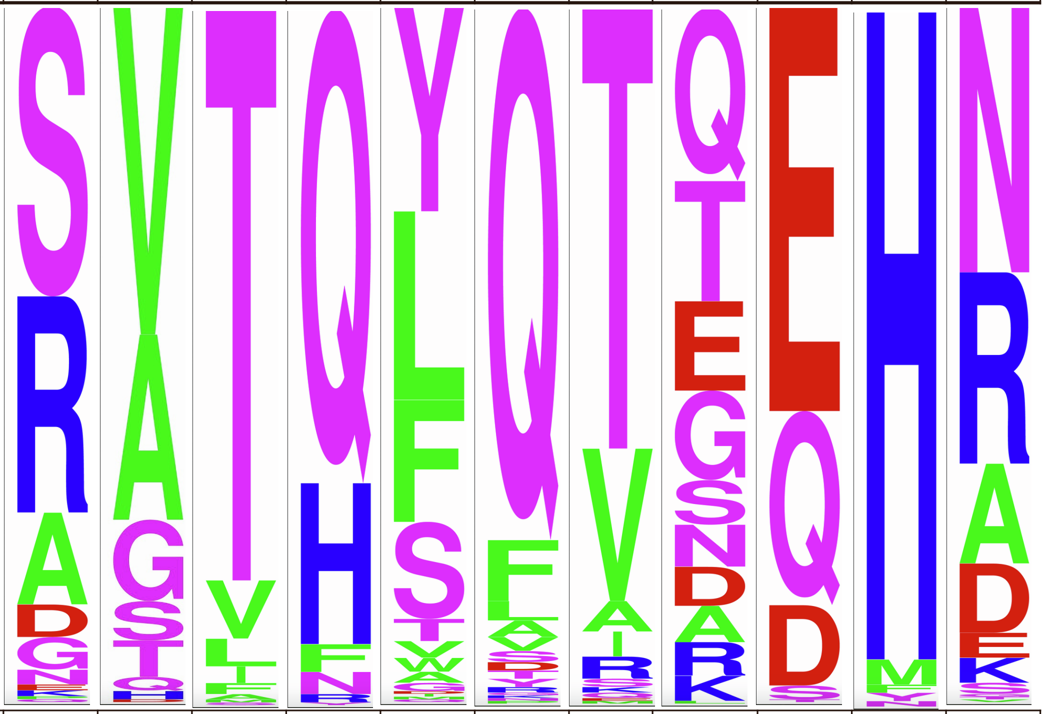

Figure S3: A graphical representation of amino acid identity at 11 mutated sites in the homologous PET hydrolase sequences of PHL7. Native amino acid identity and position in PHL7-WT are shown at the top, and the mutations found in the PHL7 variants are shown at the bottom. Out of 250 sequences, that were found from PSI-BLAST, frequency of the PHL7 mutation was calculated based on how many times that mutation appeared in the homologs.

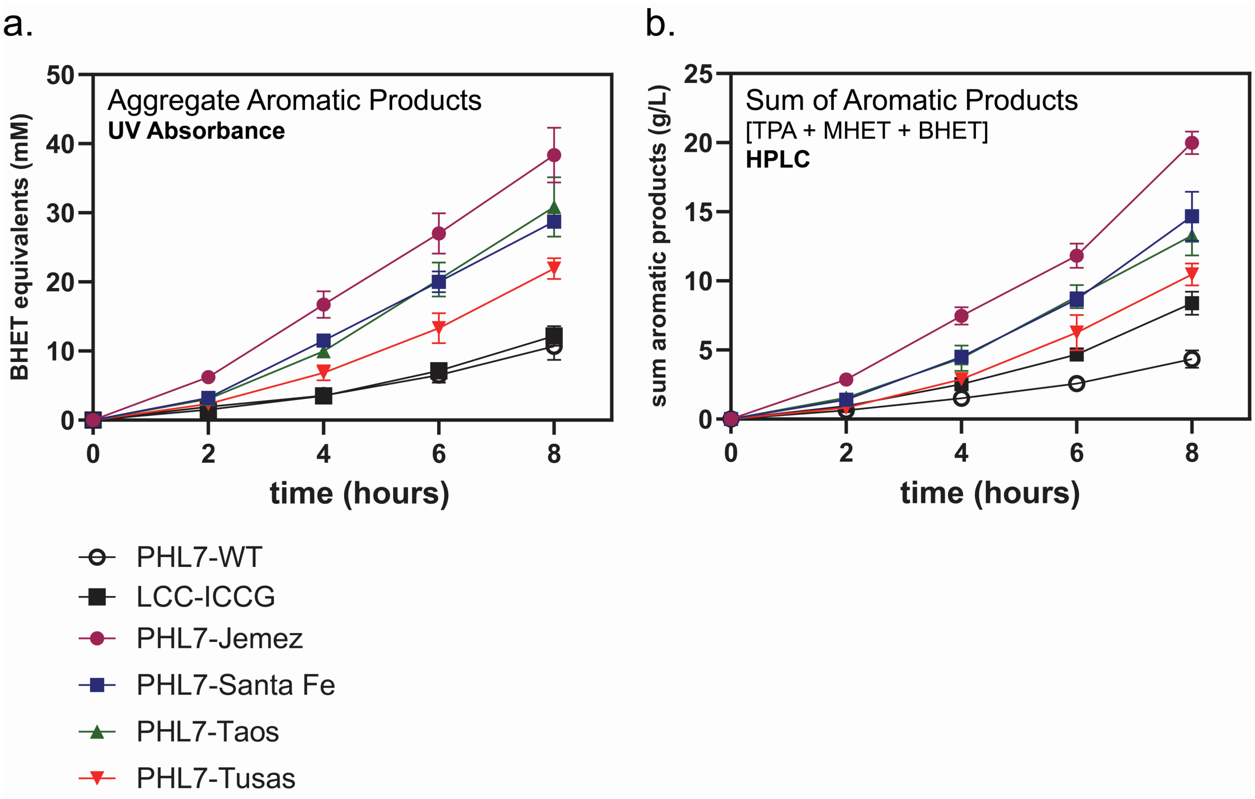

Figure S4: Comparison of UV absorbance and HPLC techniques for estimation of PET hydrolase activity. Data is shown for PHL7-WT (white circles), LCC-ICCG (black squares), and four engineered PHL7 variants (colored shapes). Reactions included 0.7 mg purified enzyme/g PET, 2.9% (w/v) PET coupons, at 70 °C and pH 8, over 8 h. **a.** Activity of the enzymes, measured by UV absorbance, expressed as equivalents of BHET. UV absorbance measures aggregate aromatic products. **b.** Activity of the enzymes, measured by HPLC. Individual monomers quantified were summed. The two analysis methods give similar results. Points display the average of n = 3 reactions, while error bars display ±1 S.D.

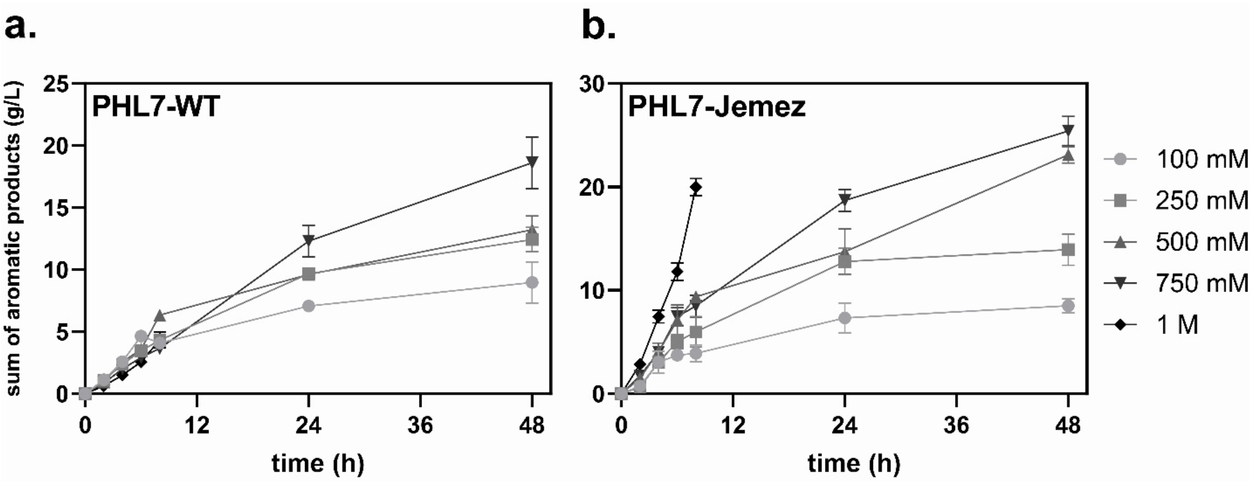

Figure S5: Comparing performance of PHL7-WT and PHL7-Jemez in different concentration buffers. Data shows activity of the variants at a gradient of potassium phosphate buffers (pH 8) at 100 to 1000 mM (see legend). Sums of aromatic products (from HPLC analysis) are shown for reactions. All experiments were conducted with 2.9% (w/v) amorphous PET film coupons at 70 °C with 0.7 mg enzyme / g PET. For the 1 M concentration, data only extends to 8 h because at 24 h time point, the PET substrate was entirely consumed by PHL7-Jemez and mostly consumed by PHL7-WT, so the reaction was stopped and no further measurement was made. Points display the average of n = 3 reactions, while error bars display ±1 S.D. **a.** Performance of PHL7-WT. **b.** Performance of PHL7-Jemez.

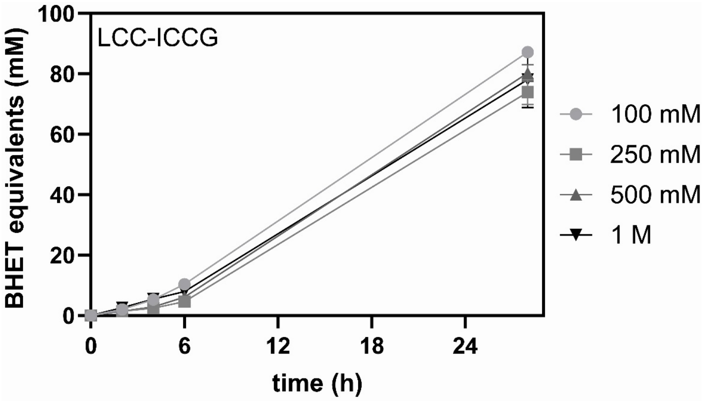

Figure S6: Comparing performance of LCC-ICCG in different phosphate buffer concentrations. Data shows activity of LCC-ICCG at a gradient of potassium phosphate buffers (pH 8) at 100 to 1000 mM (see legend) over time, to 28 h. Activity was measured using the UV absorbance assay, quantifying soluble aromatic products, and expressed as equivalents of BHET. All experiments were conducted with 2.9% (w/v) amorphous PET film coupons at 70 °C with 0.1 μM enzyme. Points display the average of n = 2 reactions, while error bars display ±1 S.D.

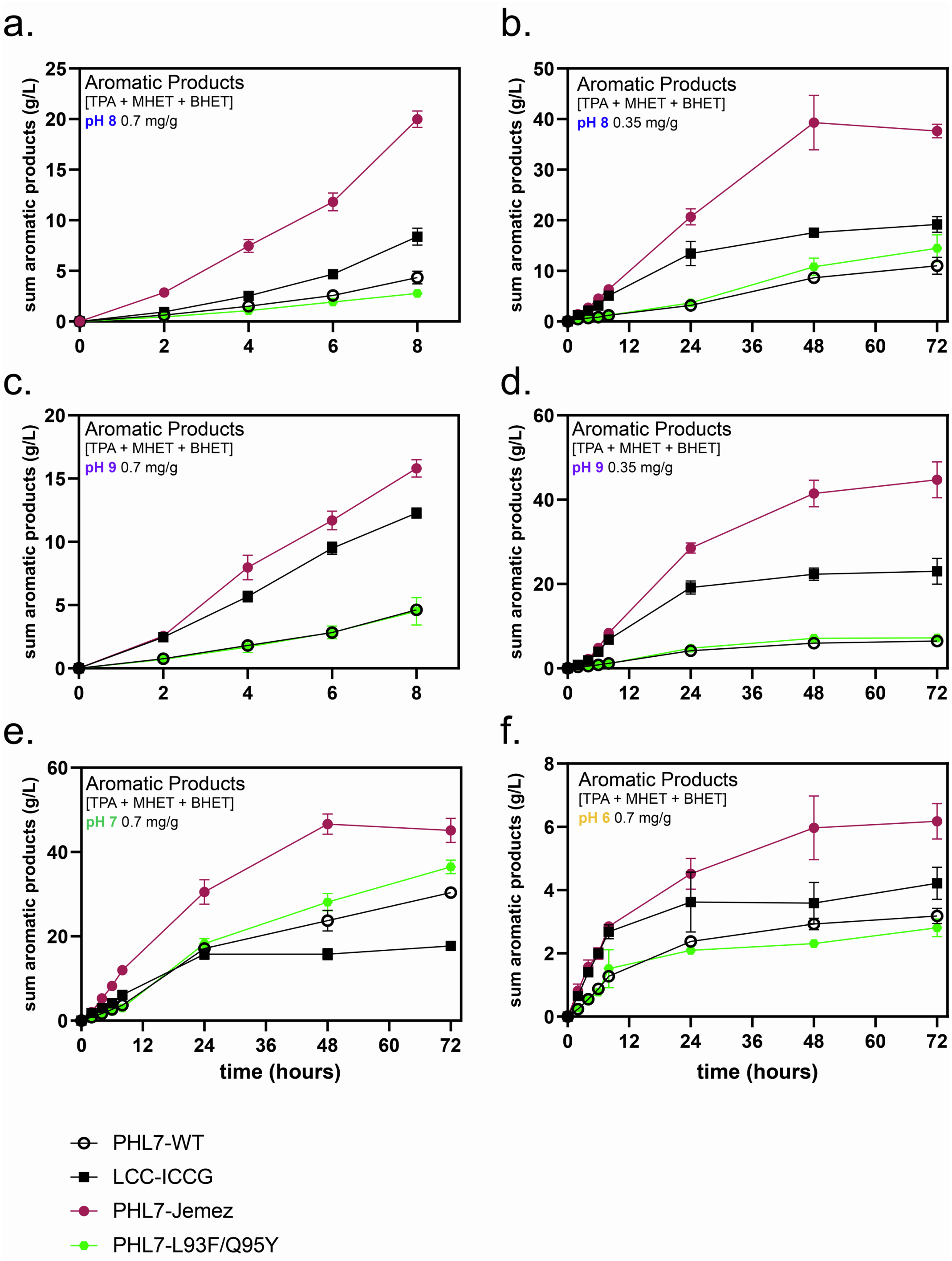

Figure S7: Comparing LCC-ICCG and PHL7 benchmarks. Data shows activity of benchmarks PHL7-WT (white circles), PHL7-L93F/Q95Y (green hexagons), and LCC-ICCG (black squares), along with top performing variant PHL7-Jemez (magenta circles). Sums of aromatic products (from HPLC analysis) are shown for reactions at varied pH. All experiments were conducted with 2.9% (w/v) amorphous PET film coupons at 70 °C. Points display the average of n = 3 reactions, while error bars display ±1 S.D. **a.** Initial rate, pH 8. **b.** Activity over time, pH 8. **c.** Initial rate, pH 9 **d.** Activity over time, pH 9. **e.** Activity over time, pH 7. **f.** Activity over time, pH 6.

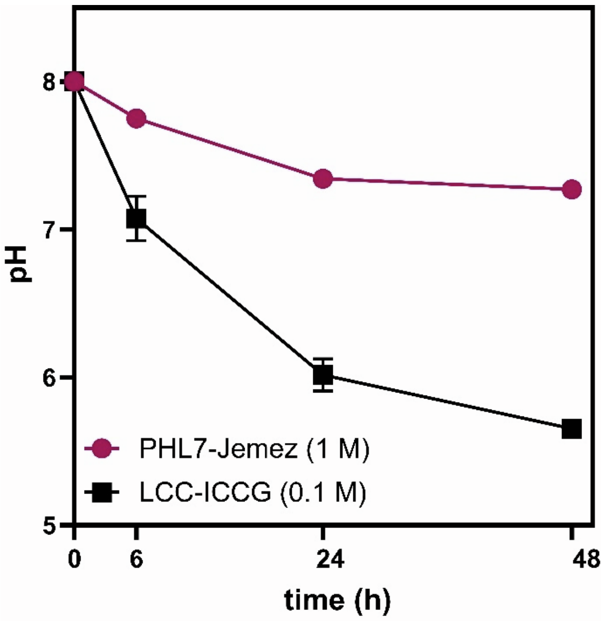

Figure S8: Change in pH from PET hydrolysis reaction. Reactions were conducted with 2.9% (w/v) amorphous PET film coupons at 70 °C with 0.7 mg enzyme / g PET, in either 1 M potassium phosphate buffer (PHL7-Jemez, magenta) or 100 mM potassium phosphate buffer (LCC-ICCG, black). Reactions were monitored over time for pH. Points display the average of n = 3 reactions, while error bars display ±1 S.D.

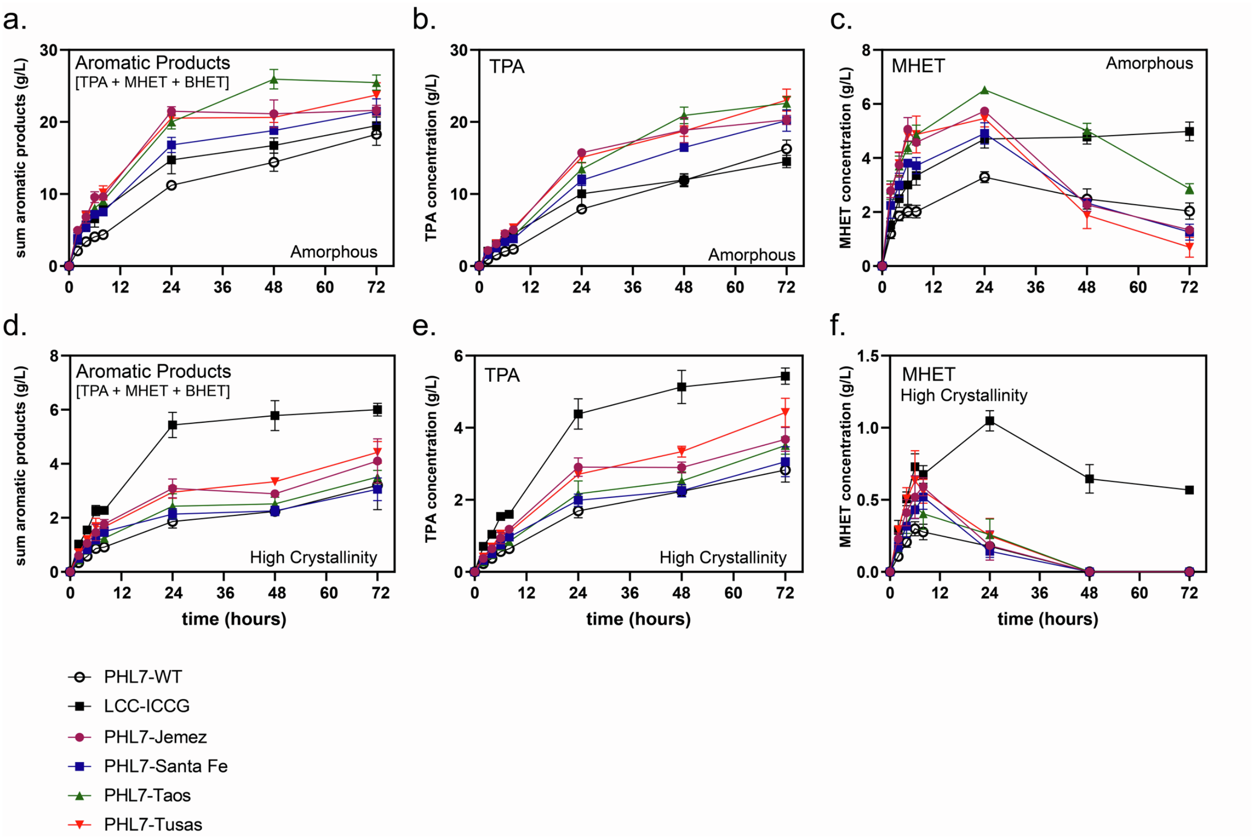

Figure S9: Activity of PET hydrolases with PET powder substrates. Data shows activity of PHL7-WT (white circles), LCC-ICCG (black squares), and four engineered PHL7 variants (colored shapes). Product concentrations (from HPLC analysis) are shown for reactions with powder (milled) PET substrates, either amorphous, **a-c**, or high-crystallinity, **d-f**, over 72 h. The substrate loading was 2.9% (w/v), the enzyme loading was 0.7 mg enzyme/g PET, and the temperature was 70 °C for all experiments. Points display the average of n = 3 reactions, while error bars display ±1 S.D. a. Activity as sum of aromatic products with amorphous PET powder. **b.** TPA concentration, with amorphous PET powder. **c.** MHET concentration, with amorphous PET powder. **d.** Activity as sum of aromatic products with high-crystallinity PET powder. **e.** TPA concentration, with high-crystallinity PET powder. **f.** MHET concentration, with high-crystallinity PET powder.

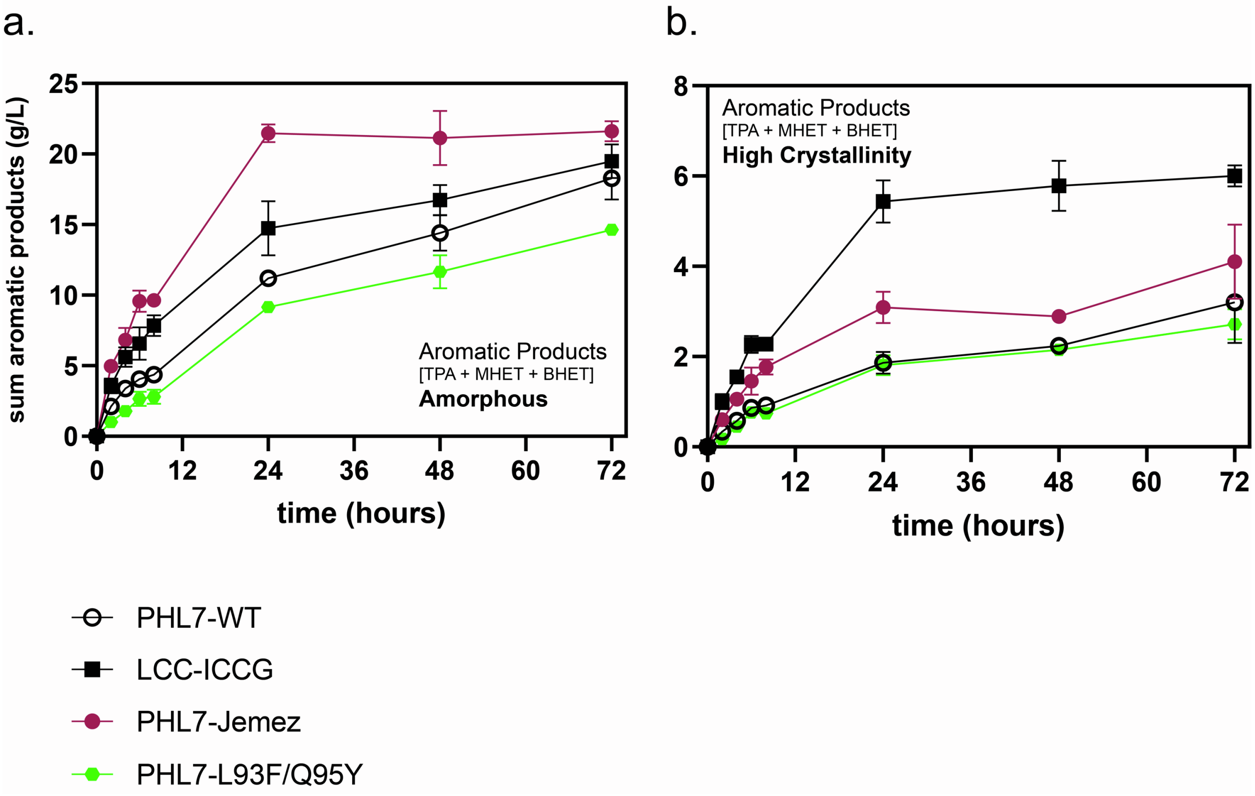

Figure S10: Comparing LCC and PHL7 benchmarks on PET powder substrates. Data shows activity of benchmarks PHL7-WT (white circles), PHL7-L93F/Q95Y (green hexagons), and LCC-ICCG (black squares), along with top variant PHL7-Jemez (magenta circles). Sums of aromatic products (from HPLC analysis) are shown for reactions on 2.9% (w/v) low- and high-crystallinity PET powder over 72 h at 70 °C and pH 8 with 0.69 µM enzyme. Points display the average of n = 3 reactions, while error bars display ±1 S.D. **a.** Sum of aromatic products, on amorphous PET powder. **b.** Sum of aromatic products, on high-crystallinity PET powder. While PHL7-Jemez outperformed all benchmarks up to 72 h on amorphous PET powder, LCC-ICCG had the highest activity on high-crystallinity PET powder.

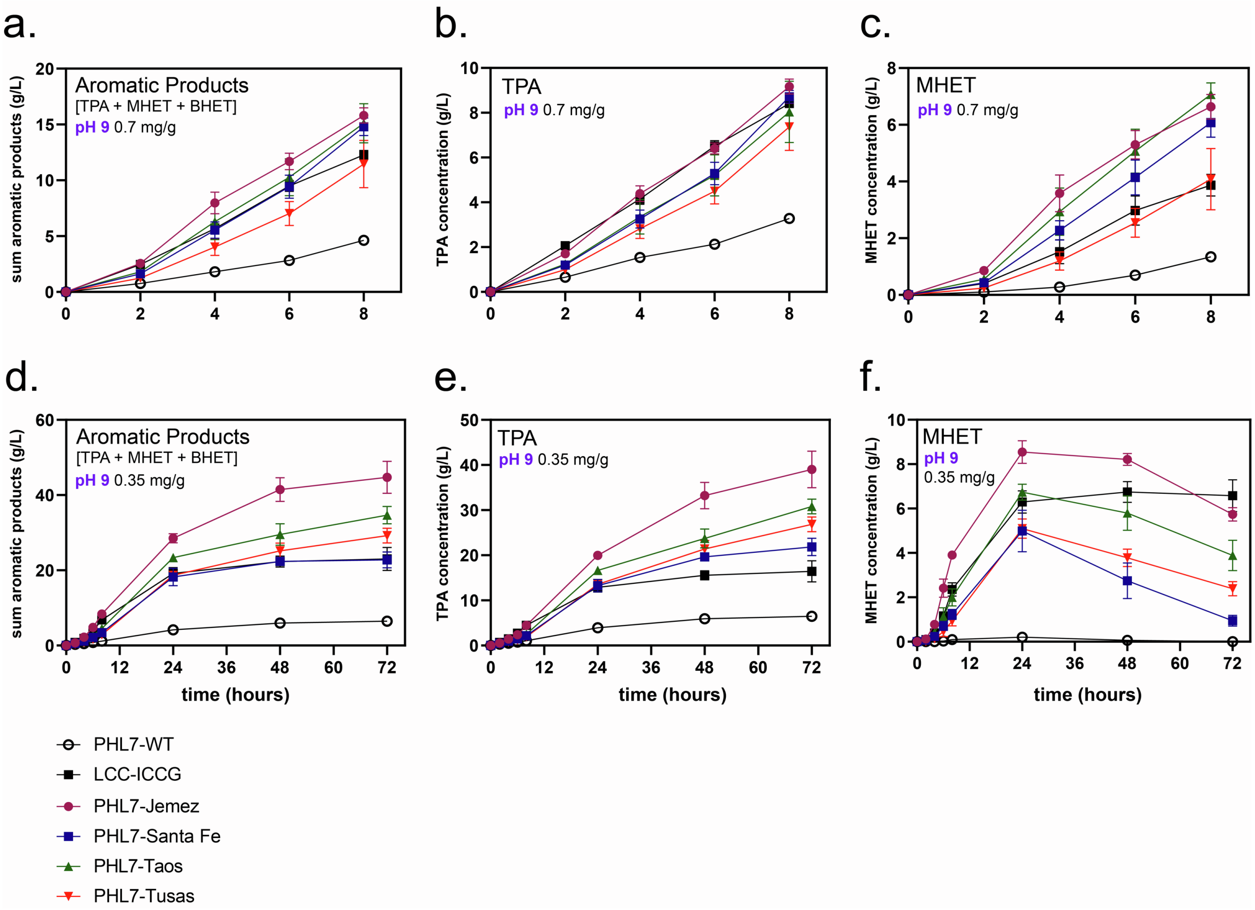

Figure S11: Enzyme activity at pH 9. Activity is shown over 8 h (0.7 mg enzyme/g PET loading) and 72 h (0.35 mg enzyme/g PET loading) for PHL7-WT (white circles), LCC-ICCG (black squares), and engineered enzyme variants (colored shapes), with reactions with 2.9% (w/v) PET coupons at 70 °C. Points display the average of n = 3 reactions, while error bars display ±1 S.D. **a.** Initial rate sum of aromatic products. **b.** Initial rate TPA concentration. **c.** Initial rate MHET concentration **d.** Sum of aromatic products, activity over time. **e.** TPA concentration, activity over time. **f.** MHET concentration, activity over time.

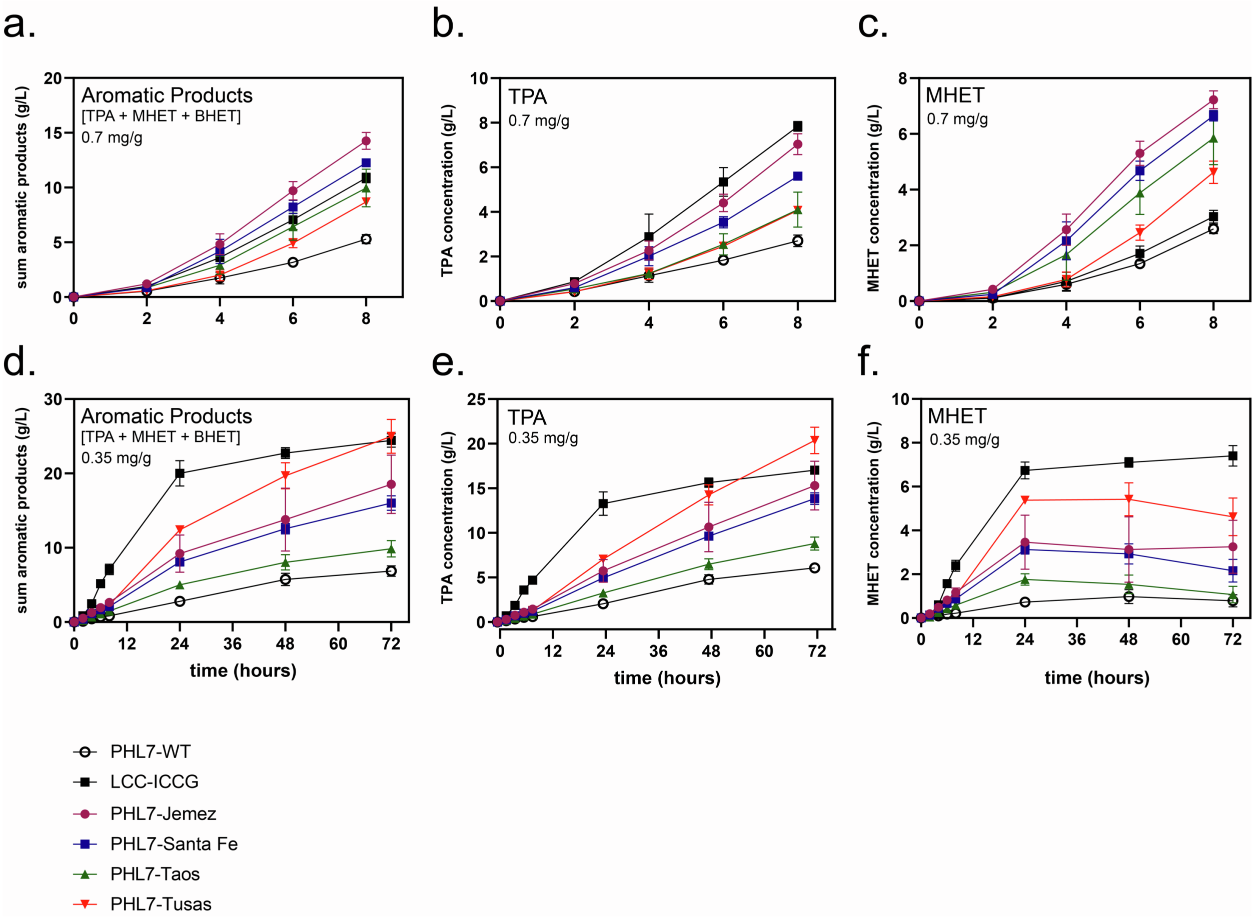

Figure S12: Enzyme activity at 65 °C. Activity is shown over 8 h (0.7 mg enzyme/g PET loading) and 72 h (0.35 mg enzyme/g PET loading) for PHL7-WT (white circles), LCC-ICCG (black squares), and engineered enzyme variants (colored shapes), with reactions with 2.9% (w/v) amorphous PET film coupons at 65 °C. **a.** Initial rate sum of aromatic products. **b.** Initial rate TPA concentration. **c.** Initial rate MHET concentration **d.** Sum of aromatic products, activity over time. **e.** TPA concentration, activity over time. **f.** MHET concentration, activity over time. Points display the average of n = 3 reactions, while error bars display ±1 S.D.

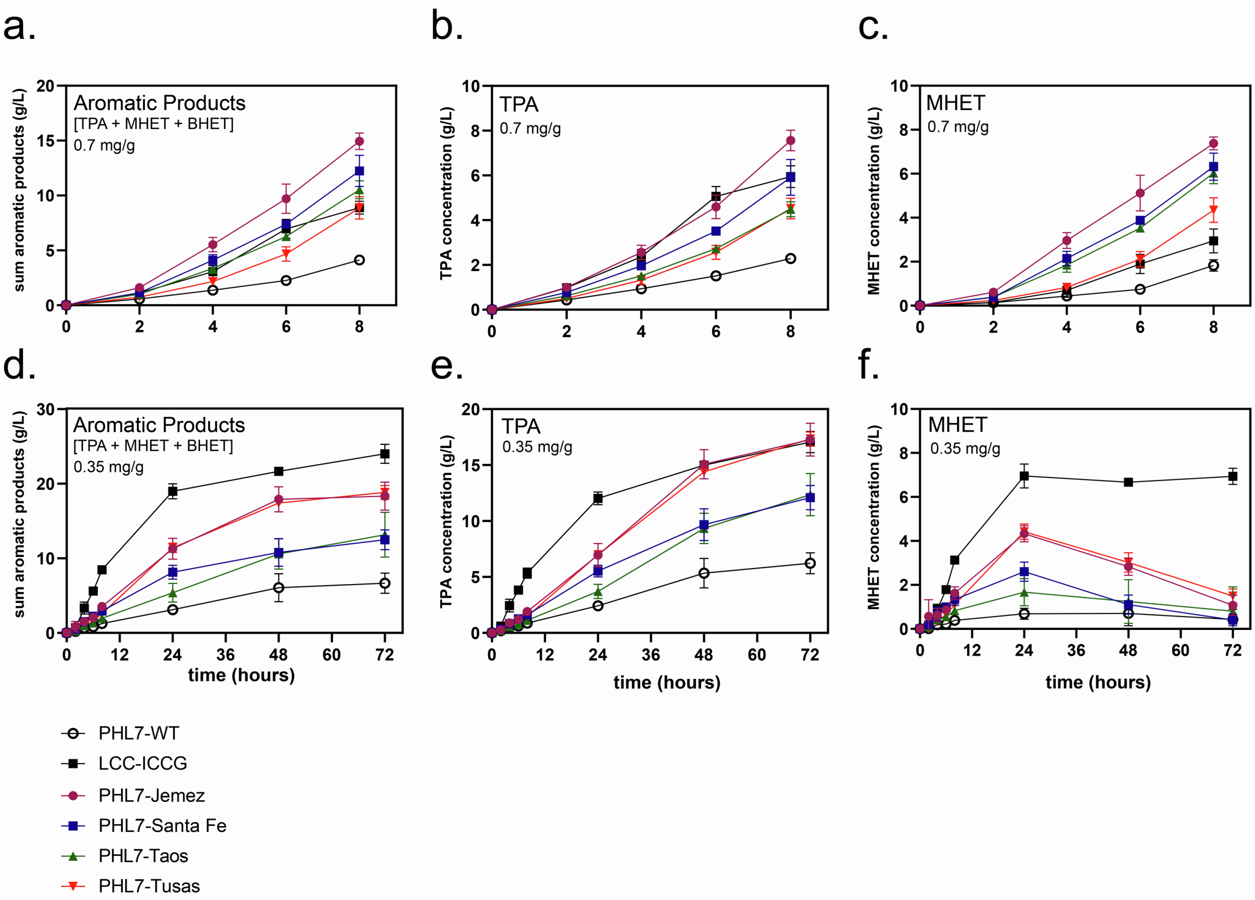

Figure S13: Enzyme activity at 68 °C. Activity is shown over 8 h (0.7 mg enzyme/g PET loading) and 72 h (0.35 mg enzyme/g PET loading) for PHL7-WT (white circles), LCC-ICCG (black squares), and engineered enzyme variants (colored shapes), with reactions with 2.9% (w/v) amorphous PET film coupons at 68 °C. **a.** Initial rate sum of aromatic products. **b.** Initial rate TPA concentration. **c.** Initial rate MHET concentration **d.** Sum of aromatic products, activity over time. **e.** TPA concentration, activity over time. **f.** MHET concentration, activity over time. Points display the average of n = 3 reactions, while error bars display ±1 S.D.

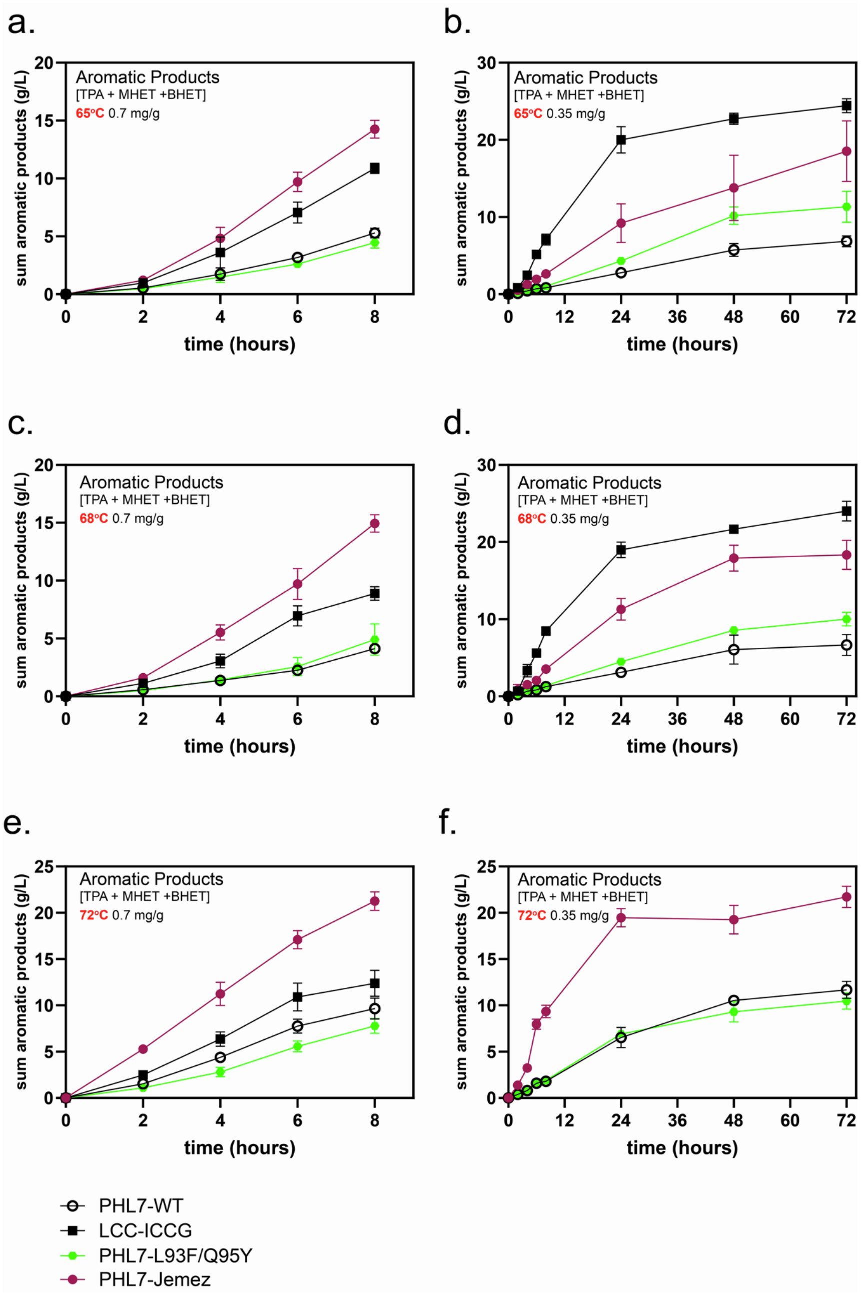

Figure S14: Comparing LCC and PHL7 benchmarks at varied temperatures. Data shows activity of benchmarks PHL7-WT (white circles), PHL7-L93F/Q95Y (green hexagons), and LCC-ICCG (black squares), along with top performing variant PHL7-Jemez (magenta circles). Sums of aromatic products (from HPLC analysis) are shown for reactions with 2.9% (w/v) PET coupons over 8 h (0.7 mg enzyme/g PET loading) and 72 h (0.35 mg enzyme/g PET loading) at varied temperatures. **a-b.** 65 °C. **c-d.** 68 °C. **e-f.** 72 °C. Points display the average of n = 3 reactions, while error bars display ±1 S.D. **a.** Initial rate, 65 °C. **b.** Activity over time, 65 °C. **c.** Initial rate, 68 °C. **d.** Activity over time, 68 °C. **e.** Initial rate, 72 °C. **f.** Activity over time, 72 °C.

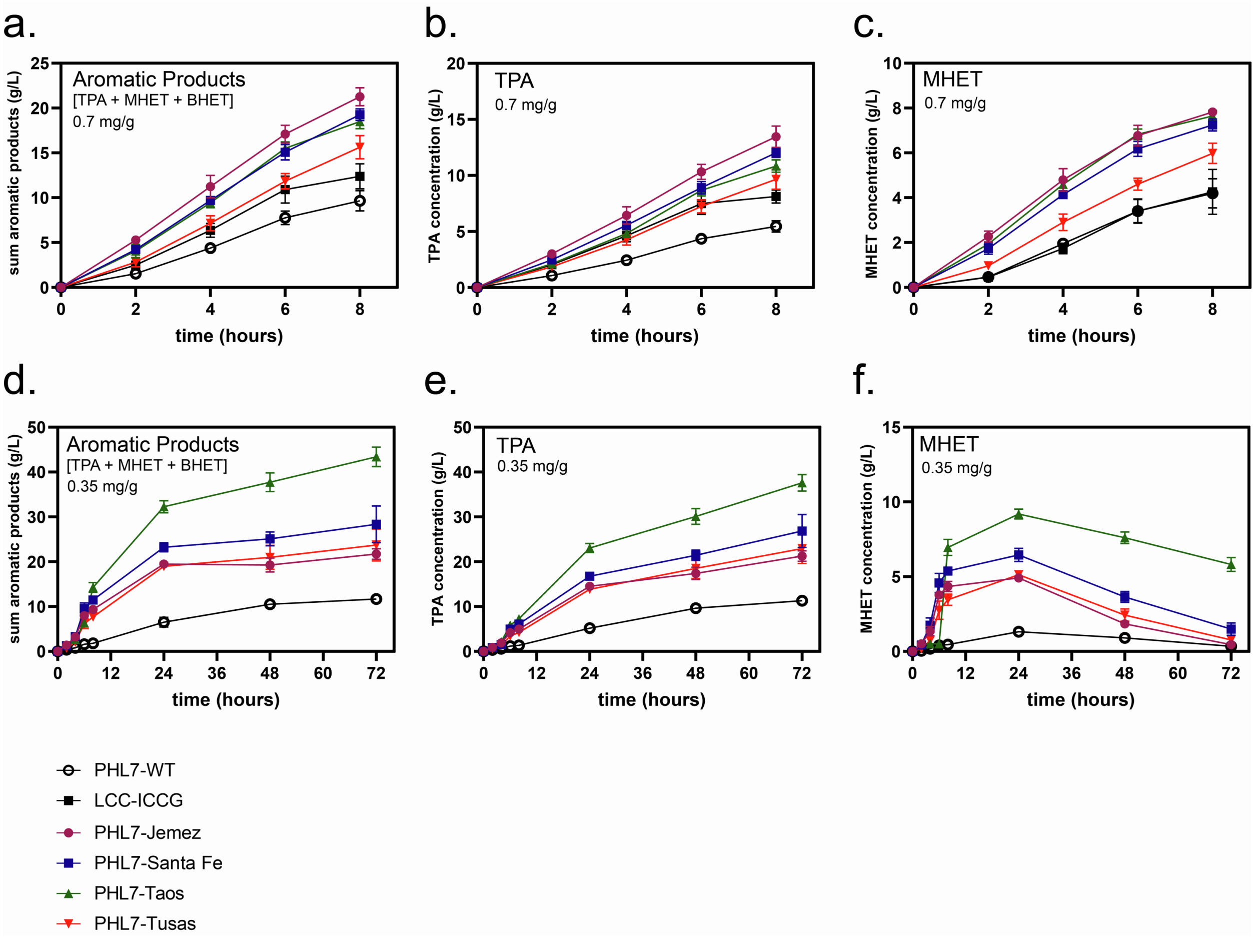

Figure S15: Enzyme activity at 72 °C. Activity is shown over 8 h (0.7 mg enzyme/g PET loading) for PHL7-WT (white circles), LCC-ICCG (black squares), and engineered enzyme variants (colored shapes), and over 72 h (0.35 mg enzyme/g PET loading) for PHL7 enzyme variants in reactions with 2.9% (w/v) PET coupons at 72 °C. **a.** Initial rate sum of aromatic products. **b.** Initial rate TPA concentration. **c.** Initial rate MHET concentration **d.** Sum of aromatic products, activity over time. **e.** TPA concentration, activity over time. **f.** MHET concentration, activity over time. Points display the average of n = 3 reactions, while error bars display ±1 S.D.

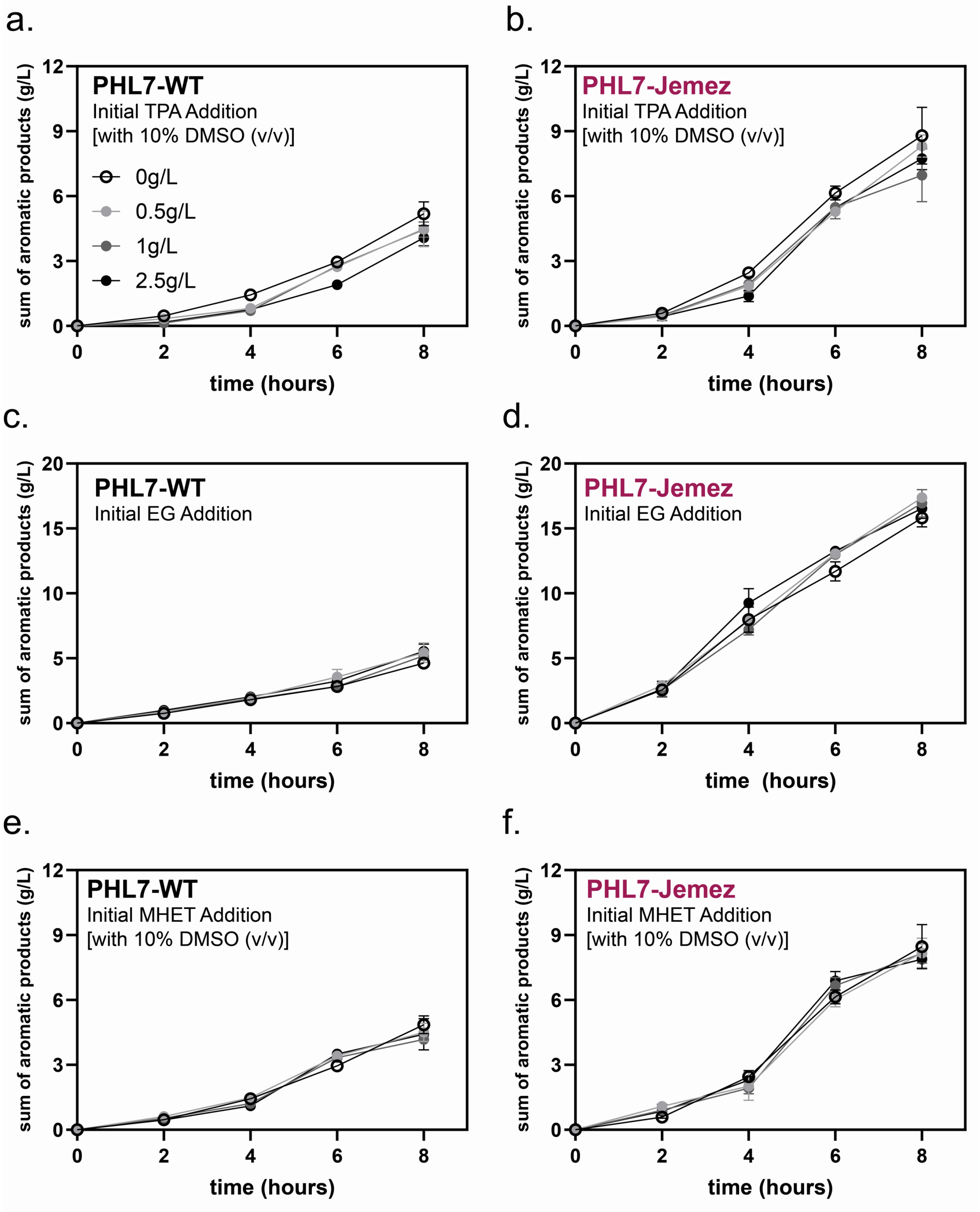

Figure S16: Product inhibition of PHL7-WT and PHL7-Jemez. Activity (from HPLC analysis) over 8 h as sum of aromatic products is shown for PHL7-WT and PHL7-Jemez with product monomers TPA, EG, and MHET initially added at varied concentrations 0 g/L (white circles), 0.5 g/L (light grey circles), 1 g/L (dark grey circles), and 2.5 g/L (black circles). Points display the average of n = 3 reactions, while error bars display ±1 S.D. DMSO [10% (v/v)] was added to TPA and MHET reactions as a consequence of solubilizing monomers. Reactions were at 70 °C, pH 8, with 0.7 mg enzyme/g PET, 2.9% (w/v) PET coupons. **a.** PHL7-WT with initial TPA. **b.** PHL7-Jemez with initial TPA. **c.** PHL7-WT with initial EG. **d.** PHL7-Jemez with initial EG. **e.** PHL7-WT with initial MHET. **f.** PHL7-Jemez with initial MHET.

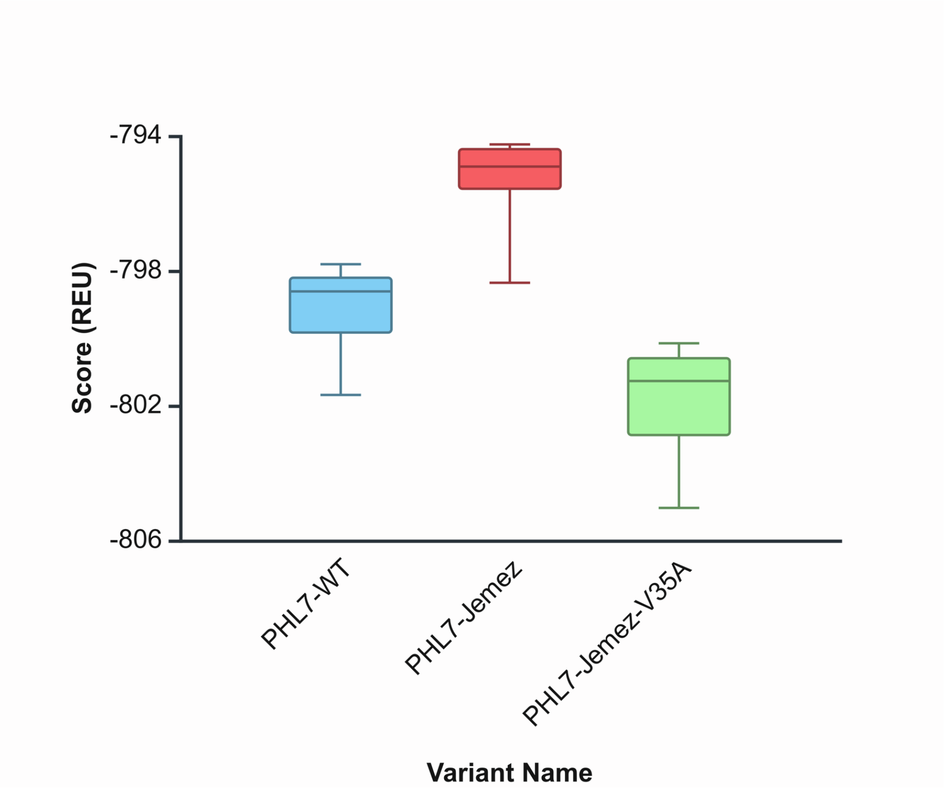

Figure S17: Box plot of ROSETTA FastDesign scores for PHL7 variants PHL7-WT, PHL7-Jemez, and PHL7-Jemez-V35A (i.e., PHL7-Jemez with the A35V mutation reverted to A35). The box plot shows the top 10% poses (26 poses for each sequence).

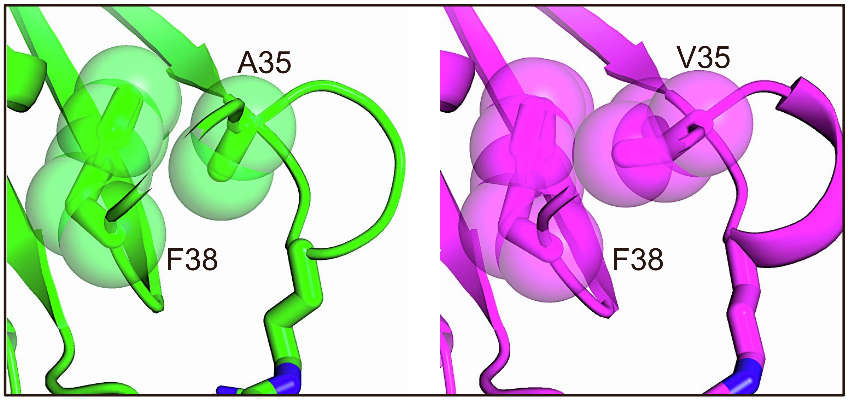

Figure S18: ROSETTA modeling of the PHL7-WT (A35) and PHL7-Jemez (V35) variants showing the region of residues 35 and 38. The modeling shows potential space filling mutation A35V improves packing with F38. PHL7-WT is shown *left*, in green and PHL7-Jemez is shown *right*, in magenta.

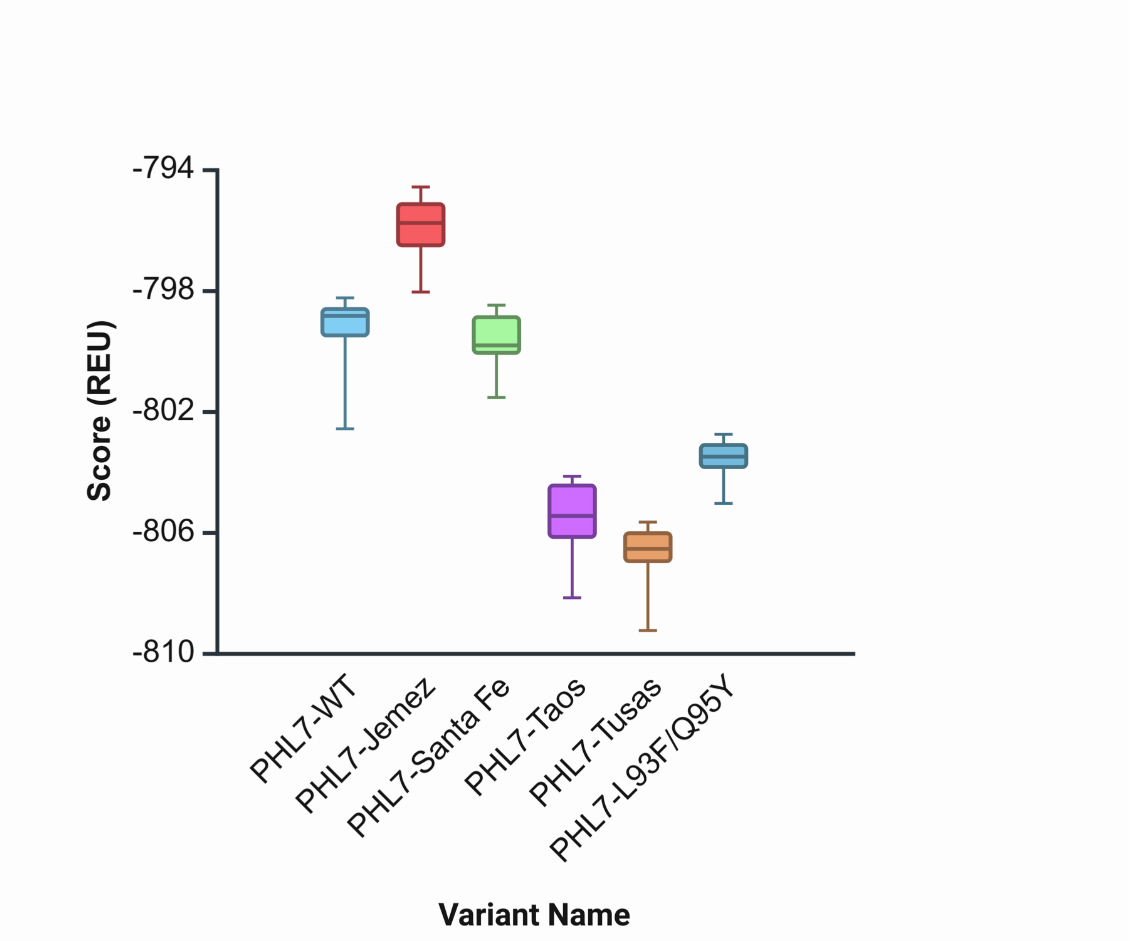

Figure S19: Box plot of ROSETTA FastDesign scores of PHL7 variants, with scores indicative of protein stability. Scores are shown for PHL7-WT, the 4 final PHL7 variants engineered in this study, and the benchmark PHL7-L93F/Q95Y.[6]

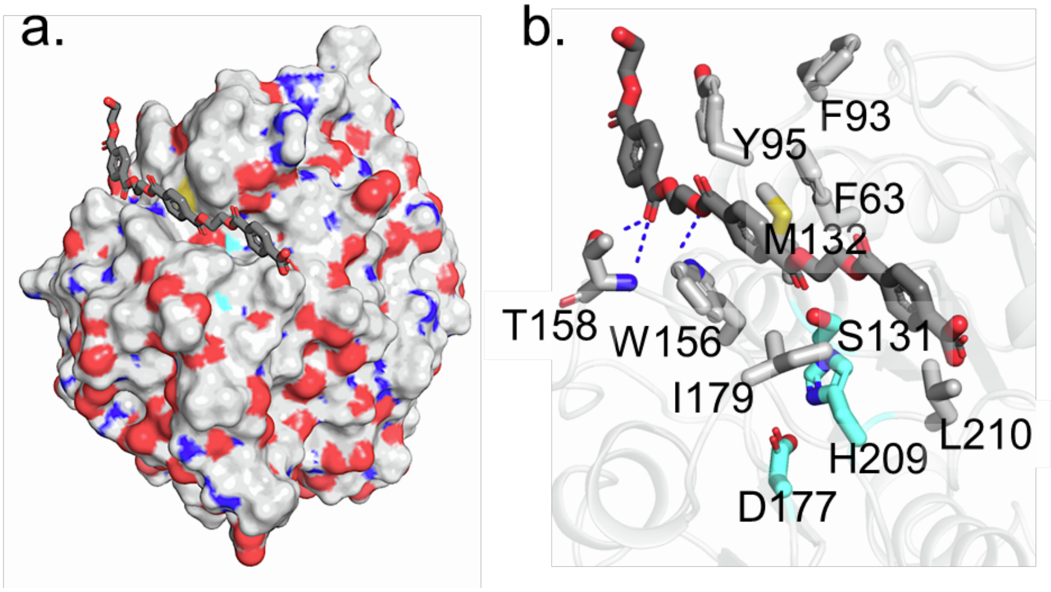

Figure S20: Computational docking of PET model substrate in the vicinity of the catalytic triad of PHL7-L93F/Q95Y**. a.** Surface representation of PHL7-L93F/Q95Y (light grey) with a bound PET model substrate (PET3mer). **b.** Key residues interacting with the PET3mer, that include L210/I179 packing against the leaving group (subsite -2), W156/F63 pi-stacking against subsite -1 and Y95 packing against subsite +1. T158 sidechain and backbone also show hydrogen bonding with the substrate. F93 fail to show any role in substrate recruitment. The catalytic triad consists of S131, D177 and H209 (cyan).

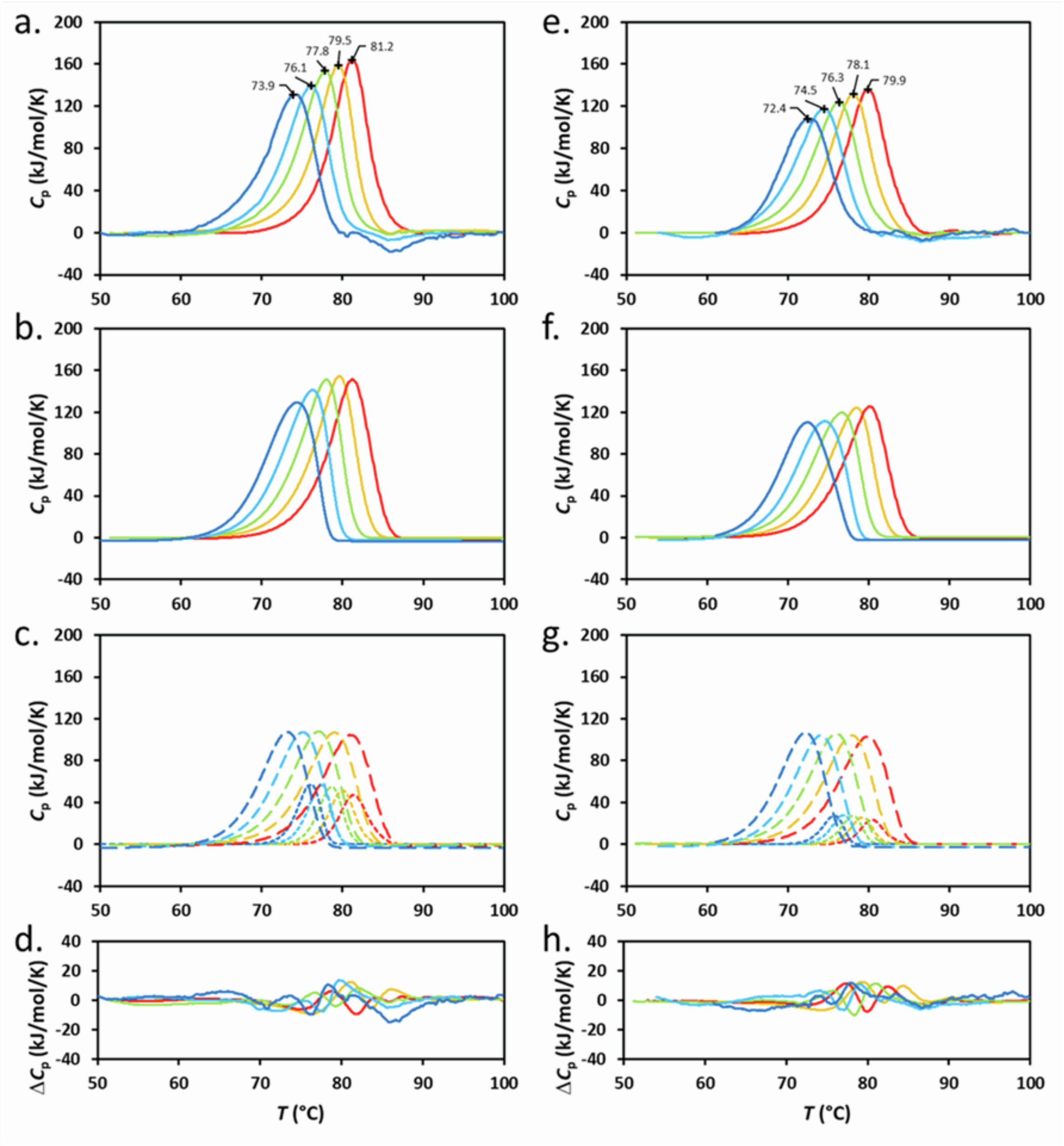

Figure S21: Multiple scan rate differential scanning calorimetry (DSC) analysis of PHL7 variants. DSC data and their analysis are depicted for **a**-**d** PHL7-WT, and **e**-**h** PHL7-Jemez. For each graph, the line color indicates the temperature scan rate in °C/min, i.e. 0.2 (dark blue), 0.4 (light blue), 0.8 (green), 1.6 (orange) and 3.2 (red). **a, e.** Overlaid experimental DSC thermograms of heat capacity (C_p) *vs.* temperature, with the apparent T_m values indicated above each. **b**, **f.** Best-fit theoretical thermograms using a two-step, irreversible denaturation model (i.e. native to intermediate to denatured). **c**, **g.** Deconvolution of the theoretical thermograms into the two transitions, native-to-intermediate (dashed line) and intermediate-to-denatured (dotted line). **d**, **h.** Difference in heat capacity (ΔC_p) between the experimental and theoretical thermograms.

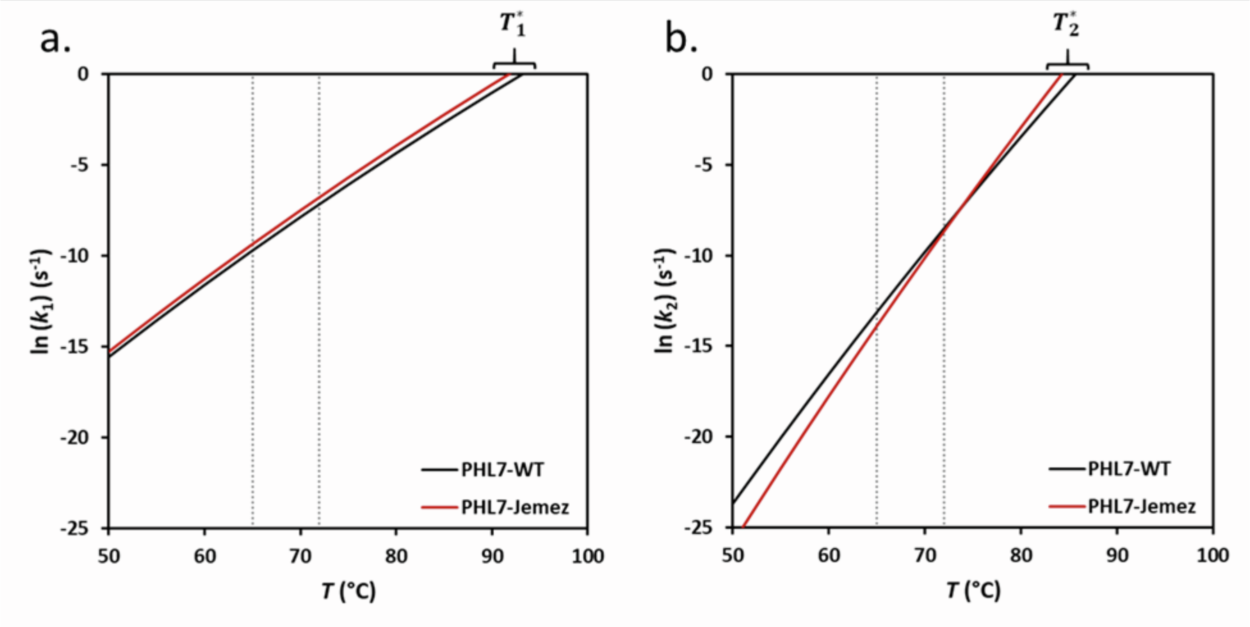

Figure S22: Temperature dependence of PHL7 variant denaturation kinetics. For PHL7-WT (black) and PHL7-Jemez (deep red), the rate constants (ln scale) are shown and a function of temperature for **a.** the native-to-intermediate transition, and **b.** the intermediate-to-denatured transition. For each temperature, the rate constants are derived from the E_a and T^* values (provided in **Table S10**), by the Arrhenius equation (see the **Methods** section in the main manuscript for details). As indicated on each graph, T^* is defined as the temperature at which $k = 1\ s^{-1}$, hence $\ln(k) = 0$. For ease of comparison, the same *y*-axis scale is used for both graphs, and the *x*-axis scale matches the thermograms in **Figure S21**. The vertical dotted grey lines indicate the upper (72 °C) and lower (65 °C) extent of the temperature range used for PET depolymerization assays.

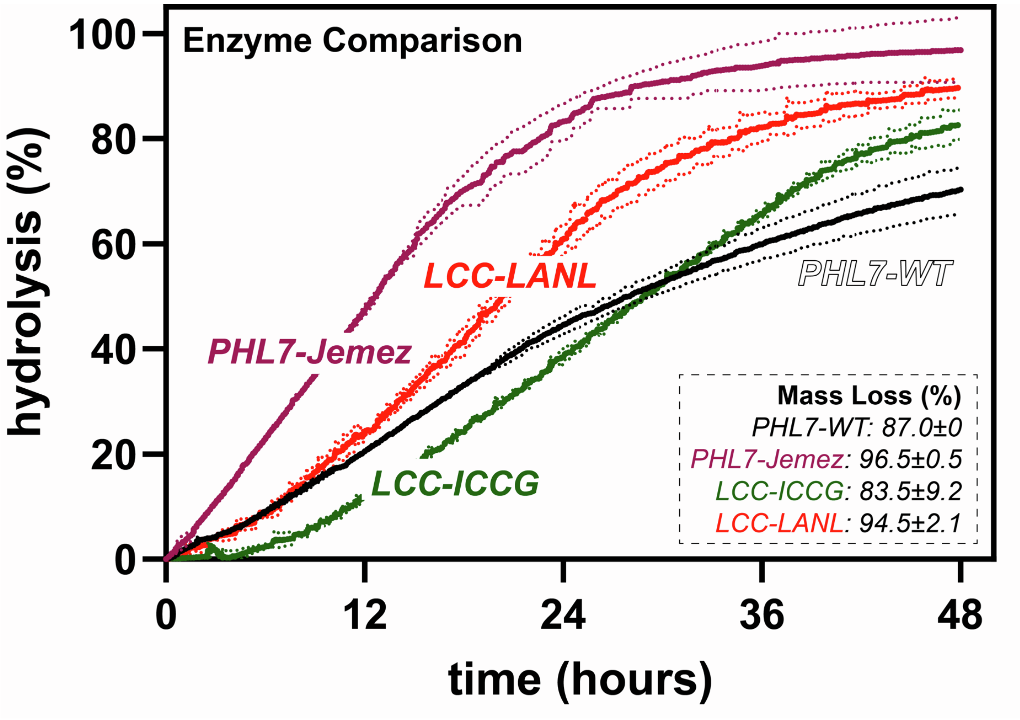

Figure S23: Comparing PET hydrolysis by benchmark enzymes in pH-controlled bioreactors. Enzymes PHL7-Jemez (magenta), LCC-LANL (red), LCC-ICCG (green), and PHL7-WT (black) were added to reactions of amorphous PET coupons [2.9% (w/v)] in bioreactors. Bioreactors were monitored for hydrolysis of amorphous PET coupons over a 48-hour reaction at 65 °C, with total mass loss observed after the end of the reaction. Data points show average of n = 2 bioreactors, while dotted lines represent ±1 S.D. Data for LCC-ICCG and LCC-LANL was obtained from our previous report.[9] Results were plotted for comparison at the conditions: pH 8, 65 °C and 2.9% (w/v) amorphous PET coupons. Note that LCC-LANL and LCC-ICCG reactions were in 100 mM sodium phosphate buffer, while PHL7-Jemez and PHL7-WT were in 1 M potassium phosphate buffer. The source of the data for LCC-ICCG and LCC-LANL is ref.[9] (at Figure 4) by Groseclose, et al. See https://pubs.acs.org/doi/10.1021/acscatal.4c04321 for more information. Further permissions related to the material excerpted should be directed to the American Chemical Society (ACS).

Supplemental References

1. Cuthbertson, A.A., Lincoln, C., Miscall, J., Stanley, L.M., Maurya, A.K., Asundi, A.S., Tassone, C.J., Rorrer, N.A., and Beckham, G.T. (2024). Characterization of polymer properties and identification of additives in commercially available research plastics. Green Chemistry *26*, 7067-7090.
2. Kawai, F., Kawabata, T., and Oda, M. (2019). Current knowledge on enzymatic PET degradation and its possible application to waste stream management and other fields. Applied Microbiology and Biotechnology *103*, 4253-4268. 10.1007/s00253-019-09717-y.
3. Tournier, V., Duquesne, S., Guillamot, F., Cramail, H., Taton, D., Marty, A., and André, I. (2023). Enzymes' Power for Plastics Degradation. Chemical Reviews *123*, 5612-5701. 10.1021/acs.chemrev.2c00644.
4. Kozlowski, L.P. (2016). IPC – Isoelectric Point Calculator. Biology Direct *11*, 55. 10.1186/s13062-016-0159-9.
5. Sonnendecker, C., Oeser, J., Richter, P.K., Hille, P., Zhao, Z., Fischer, C., Lippold, H., Blázquez-Sánchez, P., Engelberger, F., Ramírez-Sarmiento, C.A., et al. (2022). Low Carbon Footprint Recycling of Post-Consumer PET Plastic with a Metagenomic Polyester Hydrolase. ChemSusChem *15* (*e202101062*). https://doi.org/10.1002/cssc.202101062.

6. Pfaff, L., Gao, J., Li, Z., Jäckering, A., Weber, G., Mican, J., Chen, Y., Dong, W., Han, X., Feiler, C.G., et al. (2022). Multiple Substrate Binding Mode-Guided Engineering of a Thermophilic PET Hydrolase. ACS Catalysis *12*, 9790-9800. 10.1021/acscatal.2c02275.
7. Tournier, V., Topham, C.M., Gilles, A., David, B., Folgoas, C., Moya-Leclair, E., Kamionka, E., Desrousseaux, M.L., Texier, H., Gavalda, S., et al. (2020). An engineered PET depolymerase to break down and recycle plastic bottles. Nature *580*, 216-219. 10.1038/s41586-020-2149-4.
8. Kunka, A., Lacko, D., Stourac, J., Damborsky, J., Prokop, Z., and Mazurenko, S. (2022). CalFitter 2.0: Leveraging the power of singular value decomposition to analyse protein thermostability. Nucleic Acids Res *50*, W145-w151. 10.1093/nar/gkac378.
9. Groseclose, T.M., Kober, E.A., Clark, M., Moore, B., Banerjee, S., Bemmer, V., Beckham, G.T., Pickford, A.R., Dale, T.T., and Nguyen, H.B. (2024). A High-Throughput Screening Platform for Engineering Poly(ethylene Terephthalate) Hydrolases. ACS Catalysis, 14622-14638. 10.1021/acscatal.4c04321.
